# Outpatient paracentesis for the management of ovarian hyperstimulation syndrome: study protocol for the STOP-OHSS randomised controlled trial

**DOI:** 10.1136/bmjopen-2023-076434

**Published:** 2024-01-22

**Authors:** David Alexander White, Clare Pye, Katie Ridsdale, Munyaradzi Dimairo, Cara Mooney, Jessica Wright, Tracey Anne Young, Ying C Cheong, Andrew Drakeley, Raj Mathur, Alicia O'Cathain, Lauren Desoysa, Anya Sizer, Elizabeth Lumley, Robin Chatters, Mostafa Metwally

**Affiliations:** 1 Clinical Trials Research Unit, School of Health and Related Research, The University of Sheffield, Sheffield, UK; 2 Jessop Wing, Sheffield Teaching Hospitals NHS Foundation Trust, Sheffield, UK; 3 Sheffield Centre for Health and Related Research, The University of Sheffield, Sheffield, UK; 4 Obstetrics and Gynaecology, University of Southampton, Southampton, UK; 5 Hewitt Fertility Centre, Liverpool Women’s NHS Foundation Trust, Liverpool, UK; 6 Manchester Academic Health Sciences Centre, Manchester University NHS Foundation Trust, Manchester, UK; 7 Fertility Network, London, UK; 8 The University of Sheffield, Sheffield, UK; 9 Sheffield Teaching Hospitals NHS Foundation Trust, Sheffield, UK

**Keywords:** Reproductive medicine, GYNAECOLOGY

## Abstract

**Introduction:**

Ovarian hyperstimulation syndrome (OHSS) is the most significant short-term complication of pharmacological ovarian stimulation. Symptoms range from mild abdominal discomfort to rare complications such as renal failure, thromboembolism and respiratory distress syndrome.

Currently, clinical practice typically involves monitoring the patient until the condition becomes severe, at which point they are admitted to hospital, where drainage of ascitic fluid (paracentesis) may take place. Preliminary studies have indicated that earlier outpatient paracentesis may reduce the progression of OHSS and prevent hospitalisation in women.

**Methods and analysis:**

This UK, multicentre, pragmatic, two-arm, parallel-group, adaptive (group sequential with one interim analysis), open-label, superiority, confirmatory, group sequential, individually randomised controlled trial, with internal pilot will assess the clinical and cost-effectiveness and safety of outpatient paracentesis versus conservative management (usual care) for moderate or severe OHSS. 224 women from 20 National Health Service and private fertility units will be randomised (1:1) and followed up for up to 13.5 months. The primary outcome is the rate of OHSS related hospital admission of at least 24 hours within 28 days postrandomisation. The primary analysis will be an intention to treat with difference in hospitalisation rates as measure of treatment effect. Secondary outcomes include time to resolution of symptoms, patient satisfaction, adverse events and cost-effectiveness. A qualitative substudy will facilitate the feasibility of recruitment. Participant recruitment commenced in June 2022.

**Ethics and dissemination:**

London—Southeast Research Ethics Committee approved the protocol (reference: 22/LO/0015). Findings will be submitted to peer-reviewed journals and abstracts to relevant national and international conferences, as well as being disseminated to trial participants and patient groups.

**Trial registration number:**

ISRCTN71978064.

**STRENGTHS AND LIMITATIONS OF THIS STUDY**

- This is the largest randomised controlled trial to date which aims to establish whether outpatient paracentesis reduces the rate of ovarian hyperstimulation syndrome (OHSS)-related hospital admissions in those presenting with moderate or severe OHSS, compared with usual care.
- Due to the nature of the intervention, it is not possible to blind study participants or clinicians.
- Measures are taken to reduce the impact of this, including a secondary independent blinded adjudication of hospital admissions and blinding of trial statisticians.
- Patients with lived experience were involved in the design of the trial and will provide advice throughout delivery.

## Introduction

Ovarian hyperstimulation syndrome (OHSS) is the most significant short-term complications of pharmacological ovarian stimulation for assisted conception. It is most often seen in women with a high ovarian reserve, who are prone to an excessive response to gonadotropin stimulation.

There is no universally accepted classification of OHSS. In the UK, the Royal College of Obstetricians and Gynaecologists (RCOG) classification is widely used and includes mild, moderate, severe or critical categories.^1^ Moderate cases involve the build-up of fluid in the abdomen (called ascites), increased ovarian size, and abdominal distention and discomfort. In severe cases, fluid retention and dehydration can lead to changes to the constitution of the blood (haemoconcentration and hypoalbuminaemia); critical cases can lead to respiratory distress, thrombosis, disturbed renal and liver functions, and rarely death.^2^

OHSS can be further classified into early and late (table 1). Early OHSS is usually caused by the ovarian stimulation drugs given during treatment and usually occurs within 7 days after the final drug Human chorionic gonadotropin (hCG) is given. Late OHSS usually occurs 10 days or more after the administration of hCG and is caused by endogenous hCG of the resulting pregnancy. The late type is usually more difficult to control, runs a longer course and is more severe.^3^

**Table 1:** Categorisation of OHSS and its severity, based on the RCOG definitions^1^

**Categorisation of OHSS**	
Early OHSS	Caused by the ovarian stimulation drugs given during assisted reproductive technologies and occurs usually up to 7 days of the final trigger drug (hCG) being given.
Late OHSS	Usually occurs 10 or more days after the trigger drug is given.
Where a patient presents at day 8 or 9 clinical judgement will be used to classify the early or late OHSS.	
**Severity of OHSS**	
Mild OHSS	Abdominal bloating Mild abdominal pain Ovarian size is usually below 8 cm
Moderate OHSS	Patients do not meet the criteria of severe (described below) and have fluid accumulation in abdomen (ascites), confirmed by ultrasound scan. The following symptoms may also be present: Moderate abdominal pain Increased ovarian size of usually 8–12 cm Nausea with or without vomiting Some degree of clinical judgement may be needed to confirm the diagnosis of moderate OHSS.
Severe OHSS	Patients have fluid accumulation in abdomen (clinical ascites/clinically detectable fluids), confirmed by ultrasound, with or without hydrothorax The main distinguishing features are any of the following: Low urine output (oliguria) (<300 mL/day or <30 mL/hour) Haematocrit>0.45, (confirmed via full blood count (FBC) test) Ovarian size>12 cm.
Critical OHSS	Clinically obvious ascites, with one of the following features: the patient has a tense ascites or a large hydrothorax Haematocrit level >0.55 (confirmed via FBC test)* A white cell count of over 25 000 /mL (confirmed via FBC test) Anuria (very little/no urine) (<100 mL/day) Thromboembolism Acute respiratory distress syndrome

*Haematocrit level is the most important measurement.

hCG, human chorionic gonadotropin; OHSS, ovarian hyperstimulation syndrome; RCOG, Royal College of Obstetricians and Gynaecologists.

Clinical practice usually involves monitoring the patient until the condition becomes severe, when the patient is admitted to hospital, as recommended by the RCOG guideline.^1^ Inpatient management often includes drainage of ascitic fluid (paracentesis) which can result in a significant improvement of the condition and in renal blood fluid, urine output and reversal of the haematological abnormalities.^4–6^ Complications such as venous thromboembolism can have long-term health problems lasting well beyond the length of the pregnancy.

Although mild forms are fairly common (approximately one in three women undergoing IVF (in vitro fertilisation) treatment), more severe OHSS (up to 8% for combined moderate and severe OHSS)^1^ can have a significant impact on a woman’s health resulting in prolonged hospitalisation and posing a significant economic burden on both patient and National Health Service (NHS). With over 69 000 IVF cycles performed in the UK in 2019 alone,^7^ the burden of the problem becomes evident.^1^ It is, therefore, important to look into novel approaches to improve outcomes and the management of complications associated with IVF.^8^

Small retrospective studies^9 10^ and a larger uncontrolled study^11^ have suggested that transvaginal paracentesis can reduce the need for hospitalisation to between 2.9% and 14% of patients. Another small cohort study identified patients with moderate OHSS at risk of this progressing to severe OHSS and reduced this progression with only 1–3 paracentesis procedures.^12^ There is also some evidence to suggest transabdominal administration of paracentesis can prevent inpatient hospitalisation compared with those managed supportively.^13^ Furthermore, preliminary studies have safely and effectively managed patients in the outpatient setting by using a pigtail catheter to drain ascetic fluid.^14 15^ All of these studies have still achieved high pregnancy rates of 68%^13^ and 100%.^12^ Modelling also suggests that it would be more cost-effective to treat patients with moderate or severe OHSS with early outpatient paracentesis (OP), compared with less active management and inpatient admission.^16^

Preliminary results have been encouraging and suggest promising safety and effectiveness of OP.^9–13 15 17^ However, this needs to be evaluated in a robust and adequately powered randomised controlled trial (RCT) taking into consideration variabilities in practice across fertility units.

This study aims to establish the clinical and cost-effectiveness, safety and acceptability of OP as an active management for women with moderate or severe OHSS. The primary objective is to establish whether OP reduces the rate of OHSS related hospital admissions in those presenting with moderate or severe OHSS compared with usual care (UC). Secondary objectives are to:

1. Establish whether OP prevents the escalation of OHSS severity.
2. Establish whether OP reduces the time taken for OHSS symptoms to resolve.
3. Establish the safety of OP as an active intervention for moderate or severe OHSS.
4. Explore whether OP would improve patient satisfaction and quality of life.
5. Establish whether OP is cost-effective by examining healthcare resource use and patient costs.
6. Facilitate the feasibility of conducting the RCT by identifying problems with the conduct of the RCT during an internal pilot.

Pretrial development work involved qualitative interviews with clinicians and patients exploring the feasibility and acceptability of earlier active management protocols. These results fed into a consensus event to confirm the treatment protocol used in the RCT. We also undertook a comprehensive audit of proposed RCT centres to inform the parameters used to design the RCT.

## Method and analysis

Full detailed methods of the STOP OHSS trial are included in the trial protocol, available via the trial registry (https://www.isrctn.com/ISRCTN71978064).

**Figure 1:**
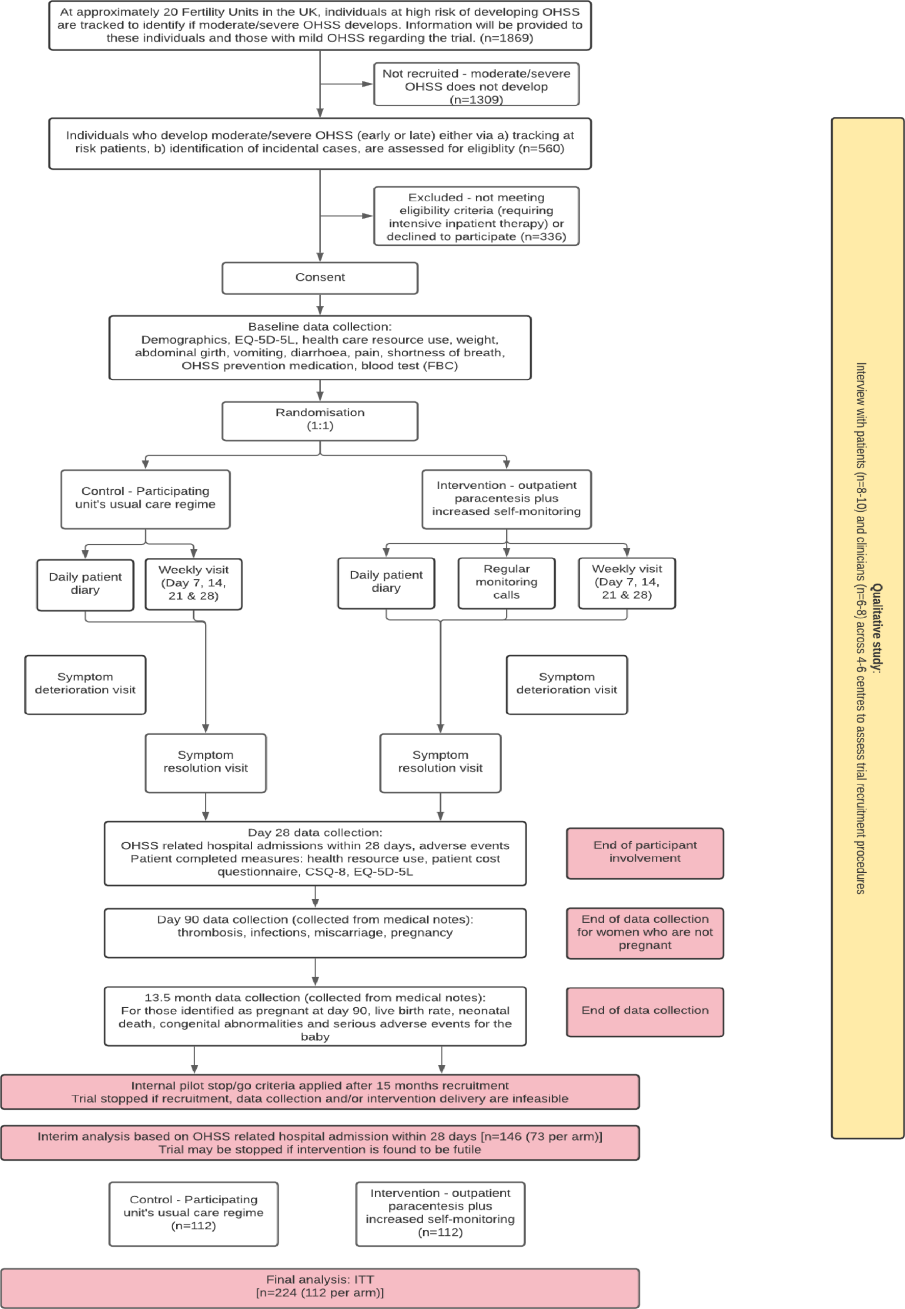
Study flow diagram. CSQ-8, Client Satisfaction Questionnaire 8; FBC, full blood count; OHSS, ovarian hyperstimulation syndrome.

STOP-OHSS is a pragmatic, parallel open-label, multicentre, superiority, adaptive, group sequential, confirmatory RCT with an internal pilot (after 15 of 31 months recruitment) to assess feasibility aspects (figure 1). Eligible participants will be individually randomised (1:1) to receive either routine treatment or OP plus daily diary monitoring.

The trial is a collaboration between The Jessop Wing, Sheffield Teaching Hospital NHS Foundation Trust and the University of Sheffield—Clinical Trials Research Unit (CTRU) who are responsible for the conduct of the trial. It is funded by the National Institute of Health and Care Research Health Technology Assessment, who had no involvement in the study design or conduct; or the decision to submit the protocol for publication. The trial will be conducted in compliance with the protocol, Good Clinical Practice and regulatory requirements. Substantial protocol amendments will be communicated to all relevant bodies. Main trial recruitment commenced June 2022 and is ongoing.

### Internal pilot

An internal pilot will use the same procedures as the main trial and will assess the following after 15 months of a planned 31-month recruitment period:

1. Participant recruitment (both arms).
2. Retention of randomised participants (both arms).
3. Delivery of the OP treatment (only OP arm).

At the end of the pilot phase, the trial steering committee (TSC) will report to the funder on whether criteria have been met to continue.

### Recruitment

The trial will recruit women attending approximately 20 NHS or private UK Fertility Units, undergoing assisted reproductive technologies (including IVF, intracytoplasmic sperm injection and intrauterine insemination) and who experience moderate or severe, early or late OHSS symptoms. Participation is voluntary and choosing not to participate will not negatively influence the woman’s treatment. Consent can be withdrawn at any stage. Women will be identified by the following recruitment strategies.

Monitoring cases—women who are considered at risk of developing moderate or severe OHSS due to various risk factors such as a previous episode of OHSS, high response to stimulation with increased follicular activity, >15 eggs with or without symptoms of OHSS, polycystic ovary syndrome, high levels of anti-Müllerian hormone (greater than 30 pmol/L, younger women (<30 years) who have a good ovarian reserve and are, therefore, known to respond well to treatment, low body mass index or any symptoms of OHSS such as bloating and nausea. Incident cases—women with moderate or severe OHSS who were not previously identified as at risk.

Potentially eligible participants will be approached as soon as possible after the diagnosis of moderate or severe OHSS and, where possible, initial eligibility screening has taken place. Women may also be alerted to the trial via the trial website or posters displayed at the fertility unit.

Research staff will provide the patient with the participant information sheet and/or link to a trial animation video outlining the study as soon as they become aware the woman is potentially eligible. Prior to randomisation, full written informed consent (see online supplemental file 1) will be obtained by a suitably trained clinician researcher at the clinic visit. Standard consent includes a provision for anonymised data to be shared with other researchers. Baseline data will be collected at this visit and participants will be randomised.

10.1136/bmjopen-2023-076434.supp1 Supplementary data

Women will be considered suitable if they meet the following eligibility criteria.

Inclusion criteria:

1. Women presenting with moderate or severe, early or late OHSS.
2. Patients able and willing to attend weekly follow-up appointments in person or remotely, daily remote appointments/phone calls and able to undertake self-monitoring at home.

Exclusion criteria:

1. OHSS-related exclusion criteria:
  - Significant pain or vomiting requiring hospitalisation.
  - Pulmonary embolism.
  - When in the judgement of the clinician, the patient’s condition is severe enough to warrant admission to a high-dependency care unit (such as critical OHSS as defined in the RCOG green-top guidelines), and therefore, not suitable for outpatient management.
2. Non-OHSS-related medical conditions: a concurrent medical condition requiring immediate inpatient management.
3. Patients who have been previously randomised but later present with moderate or severe OHSS symptoms in subsequent cycles after their initial trial involvement.
4. Participation in other trials involving ovarian stimulation or ovarian response.

### Centralised research nursing delivery team

Local research teams will be supported in recruiting participants and ongoing management of the trial protocol procedures from the central research nursing delivery team based at the Jessop Wing, Sheffield Teaching Hospitals NHS Foundation Trust. This team will support local discussions with potential participants about trial participation, randomisation procedures and monitoring and routine follow-up visits in both trial arms.

### Randomisation and concealment

Patients will be randomised using a centralised web-based system (SCRAM) hosted by Sheffield CTRU, which has user-restricted functionalities granting access rights to specific areas as appropriate. The sequence will be computer generated using permuted block randomisation stratified by recruiting site and severity of OHSS. Block sizes will be disclosed during dissemination of findings and only the randomisation (trial) statistician(s) not involved in the recruitment process will know the block sizes during the trial. The trial statistician will generate the randomisation sequence; however, they will not have access to the generated sequence. Research staff at recruiting centres will use a web-based computer system with restricted access rights to enter participant details; randomisation outcome will then be revealed. Re-randomisation will not be permitted.

### Intervention and control

Following randomisation, women in the intervention arm will have OP performed in the outpatient setting as soon as clinically possible. Sites will follow their own local procedures for conducting paracentesis either abdominally or vaginally by a suitably trained doctor, advanced nurse practitioner or radiologist in the fertility, gynaecology or radiology unit and while the trial does not dictate how to perform the intervention, guidance will be offered.

If rehydration is required via intravenous methods, this will be at the discretion of the attending clinician. If a large volume of fluid is removed, then intravenous colloid therapy should be considered. If this administration of fluid results in overnight inpatient hospitalisation it will not be viewed as meeting the trials primary outcome relating to hospitalisation. Some patients may require multiple drainage which can be performed if the participant has a reaccumulation of ascites.

Women in the UC group will not have the paracentesis performed in an OP setting and will be monitored by their clinical team as per UC. If UC at the site involves administering paracentesis on an outpatient basis for treatment of OHSS then the site will be requested to stop routinely providing this for participants in the UC arm.

Both groups of women will be provided with access to a diary (either in paper format or sent electronically using Research Electronic Data Capture (REDCap)^18^ hosted at The University of Sheffield for the purpose of daily self-report of weight, abdominal girth, diarrhoea, vomiting, pain, shortness of breath, fluid input and urine output. This information will be used by the clinical team in the intervention arm on a daily basis to inform further management and whether to perform further OP. The UC group will also perform daily self-monitoring, but these data will be sent directly to CTRU for input into the database and will not be available to be reviewed by site staff.

Some fertility units undertake other preventative measures (eg, dopamine agonists, gonadotropin-releasing hormone (GnRH) antagonists, cessation of IVF cycle) to reduce the risk of OHSS and details of these measures will be collected during the trial, along with information on the provision of care within the conservative management arm. Rarely, patients may be prescribed GnRH antagonists as treatment for established moderate or severe OHSS. This will not be prohibited for trial participants in both arms and data will be collected on GnRH use within the trial.

Participants may wish to stop study treatment, or there may be a clinical need to stop treatment. Participants randomised to the intervention arm, who withdraw from receiving the intervention will continue to be followed-up and will remain in the trial, unless they request to be withdrawn.

### Outcomes

Data collection points are outlined in table 2.

**Table 2:** Data collection time points

	Baseline/randomisation (day 0)	Daily until symptom resolution	Weekly (days 7, 14, 21, 28)	Symptom resolution	Symptom deterioration*	During hospitalisation	Day 90	13.5 months
Data collected by site staff								
Demographics and medical history (including medication to prevent OHSS)	OPt							
Symptoms: Fluid balance including fluid drunk, urine output‡ Diarrhoea and vomiting Nausea§ Abdominal girth Pain Shortness of breath Weight	OPt	SR	T/OP†	OPt	OPt	IP/MN		
Blood tests (FBC)—if a face-to-face appointment	OPt		T/OP†	OPt	OPt	IP/MN		
Training and provision of consumables, and record details	OPt							
EQ-5D-5L questionnaire	OPt	SR				SR/IP		
OHSS-related hospital admission within 28 days (primary outcome) and reasons for hospitalisation			T/OPt	OPt	OPt			
Intervention delivery (volume of ascites removed, number of paracentesis)			MN	OPt		MN		
Conservative management arm—details of usual care monitoring			MN	OPt				
Use of GnRH antagonist for treatment of OHSS			OPt	OPt	OPt	IP/MN		
Confirmation of symptom resolution				OPt		MN		
Adverse events (patient)		T¶	OPt / MN	OPt	OPt	IP		
Pregnancy outcome (eg, miscarriage) and ongoing pregnancies							MN	
Incidence of thrombosis or embolism or significant infection							MN	
Live birth information and pregnancy outcome (eg, miscarriage)								MN
Neonatal death and SAEs related to the baby								MN
Data collected by the central study team								
Health resource use questionnaire			Q (day 28 only)					
CSQ-8 questionnaire			Q (day 28 only)					
Patient cost questionnaire			Q (day 28 only)					
EQ-5D-5L questionnaire			Q (day 28 only)					

*If symptoms deteriorate to the point where hospital admission is thought to be required.

†If OHSS symptoms have not resolved by this time point.

‡Fluid balance will be self report throughout.

§Nausea will not be collected as part of the daily patient diary.

¶This will only be possible for participants who are being contacted daily. When they are not being contacted daily it will be asked at every contact.

CSQ-8, Client Satisfaction Questionnaire 8; FBC, full blood count; IP, inpatient; MN, medical notes; OHSS, ovarian hyperstimulation syndrome; Opt, outpatient; Q, data collected via a questionnaire; SAE, serious adverse event; SR, self-reported by the patient; T, data collected over the telephone by a research nurse.

Primary outcome is any:

- OHSS-related hospitalisation for at least 24 hours within 28 days of randomisation.

Secondary outcomes:

- Need for OHSS-related hospitalisation within 28 days—independent blinded central assessment.
- Time to resolution of OHSS assessed within 28 days of randomisation.
- Progression of OHSS severity within 28 days of randomisation.
- Cumulative length of OHSS-related hospitalisation.
- Live birth, pregnancy outcomes, neonatal death and serious adverse events (SAEs) including congenital abnormalities in the new-born within 13.5 months of randomisation.
- The occurrence of thrombosis, embolism and significant infections requiring antibiotic treatment or hospitalisation within 90 days of randomisation.
- AEs within 28 days of randomisation.
- Patient satisfaction assessed using the Client Satisfaction Questionnaire 8^19^ based on total scores at 28 days postrandomisation.
- EQ-5D-5L participant quality of life daily and at 28 days postrandomisation.
- Health resource use and patient costs at 28 days postrandomisation.

### Follow-up

Data will be collected as follows:

- Participant diary data, self-completed daily until symptom resolution.
- Monitoring visits, either face to face or remotely, at days 7, 14, 21 and 28 or until symptoms have resolved.
- Symptom resolution visit, face to face (unless symptoms resolve during hospitalisation and the data can be collected from medical notes or the participant).
- A symptom deterioration visit, to assess if hospitalisation may be required.
- A participant self-completed questionnaire at 28 days.
- Remote follow-up at day 90, to collect ongoing pregnancies/pregnancy outcome (eg, miscarriage), thrombosis/embolism and significant infections. Participants not pregnant at this time point will be considered complete and will not require further data collection.
- For participants identified as pregnant at 90 days, live birth and pregnancy outcome (eg, miscarriage) as well as neonatal death, congenital abnormalities and SAEs for the baby at 13.5 months.

### Safety considerations, safety monitoring and AE reporting

All AEs and SAEs will be recorded at each participating fertility unit. All AEs/SAEs will be followed up until satisfactory resolution or until the treating clinician and the principal investigator deems the event to be chronic or the participant to be stable. Clinical/research staff will ask participants for any details of AEs at each daily contact in the intervention arm and then in both groups at all time points.

### Blinding

It will not be possible to blind participants or clinicians to treatment allocation. The trial statistician and TSC will be blinded. To assess the potential impact of outcome assessment bias, an additional analysis of the primary endpoint will be performed, using an independent blinded adjudicated primary outcome concerning whether trial participants needed OHSS-related hospitalisation within 28 days.

### Trial monitoring and oversight

The trial will be overseen by a TSC and data monitoring and ethics committee (DMEC). Membership of both will consist of independent experts in the field and the TSC includes a patient representative. The DMEC may advise the chair of the TSC at any time if, in their view, the trial should be stopped for ethical or adaptive reasons.

Day-to-day running of the trial will be coordinated by the trial management group (TMG), consisting of the grant coapplicants, plus members of the Jessop Wing Fertility Unit, Sheffield CTRU and patient representatives.

### Sample size and interim analyses

Under current conservative management, the mean OHSS-related hospitalisation rate is approximately 41.2% (26/63) based on a retrospective audit of medical records of IVF patients across six IVF fertility units over a year (95% CI 29.0% to 54.4%). Earlier active management interventions based on uncontrolled small previous studies have resulted in low hospitalisation rates of around 0%–8%.^10 12 13 20^ As such, this trial is targeting a 20% absolute reduction in hospitalisation for OP to be considered superior to UC. This reduction is believed to be realistic as previous uncontrolled studies have observed similar or greater effects and, if observed, is more convincing to change clinical practice. A 20% absolute reduction translates to an OR of 0.3825 or relative risk/risk ratio (RR) of 0.512 assuming a 41% UC event rate. Thus, the targeted effect in terms of OR or RR is a function of the UC event rate whereas the targeted 20% absolute reduction is consistent across the plausible UC event rates. An updated preliminary health economics model (trial protocol, via ISRCTN71978064) has indicated that OP needs to achieve at least 4.7%–5.7% reduction in hospitalisation to become cost saving under several assumptions about the ratio of early to late OHSS and underlying UC hospitalisation rates assuming a 20% targeted reduction.

The aim of the trial is to gather convincing evidence to influence the effects of the early OP that is most likely to change practice regardless of the direction of results and so a group sequential design with an option for early stopping for futility (lack of benefit) is used.

The trial will require a maximum total sample size of ~224 (112 per arm) with an interim analysis when ~146 participants (73 per arm) have accrued primary outcome. This assumes a 90% power, one-sided 2.5% type 1 error, 41% UC hospitalisation rate, 0% drop-out rate, a 20% reduction in hospitalisation rate and a futility threshold of −4.5% (difference in hospitalisation rate). In this case, the trial will be stopped early for futility if the observed reduction in hospitalisation rate at an interim analysis is less than 4.5%. There is only a 1.9% chance of stopping the trial early for futility in error when OP is truly beneficial. Finally, there is a 72.2% probability of stopping early when the effect of OP is the same as UC (ie, 41% hospitalisation rate) (see online supplemental file 2).

10.1136/bmjopen-2023-076434.supp2 Supplementary data

### Statistical analysis

Detailed of all analysis will be described in an open-access and prespecified SAP to be developed and signed off before accessing unblinded data.

The primary analysis will be an intention to treat, including all eligible participants randomised with informed consent. Baseline data will be summarised at the interim and final analyses by treatment group. The absolute difference in hospitalisation rates between arms is the primary summary measure of the treatment effect. RR and OR (estimated using a simple logistic regression model) will also be presented. At an interim analysis, the unadjusted difference in hospitalisation rates between arms will be calculated to inform the interim decision on whether to stop early for futility. For sensitivity analysis, a mixed effects logistic regression model adjusted for stratification factors (site as a random effect and OHSS severity as a fixed effect) and adjusted difference in hospitalisation rates will be obtained using the delta method via margins.^21^ For the final analysis of the primary outcome, the unadjusted difference in hospitalisation rates with CI obtained via normal approximation will be reported as well as the p value from a χ^2^ test. Stagewise ordering will be used for sensitivity analysis to obtain the median unbiased estimate of the difference in hospitalisation rates between arms with the associated 95% CI to be presented alongside the maximum likelihood estimate if the trial progressed beyond interim analysis. The details of additional sensitivity analyses to be performed adjusting for baseline covariates are found in the SAP. Reporting will adhere to the adaptive designs CONSORT (Consolidated Standards of Reporting Trials) Extension guidance.^22^

For progression of OHSS severity, a mixed effects logistic regression model adjusted for stratification factors (site as a random effect and OHSS severity as a fixed effect) will be used and an adjusted difference in hospitalisation rates will be obtained using the delta method via margins.^21^ Treatment effects will be presented as adjusted OR, adjusted risk difference and adjusted relative risk/RR with 95% CIs.

For time to resolution of OHSS assessed within 28 days postrandomisation, Kaplan-Meier curves will be used to visualise the resolution curves between treatment arms and differences qualified using a log-rank test. Median time to resolution by treatment arm with 95% CI will be calculated and reported as summary measures of within-group effects. Participants who fail to achieve resolution of symptoms within 28 days postrandomisation will be censored. Sensitivity analysis and alternative approaches when the proportional hazard assumption is not met will be detailed in the SAP.

For cumulative length of OHSS hospital stay, bootstrapping resampling procedure (accounting for stratification) will be used to obtain the median difference with 95% CI and associated p value. For patient satisfaction at 28 days, a total satisfaction score will be analysed using a mixed effects linear regression model adjusted for site (as a random effect) and OHSS severity (as a fixed effect), and the adjusted mean difference between treatment arms with 95% CI and associated p value will be presented.

Analysis of safety outcomes (eg, AEs and SAEs) will be based on descriptive statistics using the safety analysis population defined based on treatment-as-received population. Details of additional analysis accounting for repeated events and differential follow-up is found in the SAP.

Subgroup analyse will be performed to explore whether there is heterogeneity in treatment effect on the primary outcome across the following prespecified subgroups:

1. Baseline severity of OHSS (moderate or severe).
2. Whether a participant is taking a preventative drug at randomisation (yes or no).
3. Whether the participant has early or late OHSS at randomisation.
4. Whether the OP procedure was done vaginally or abdominally. For participants who received multiple procedure (which may happen in few cases), this classification will be based on the first performed procedure.

A mixed effects logistic regression model that includes an interaction effect between preplanned subgroup and treatment group adjusted for site (as a random effect) and severity of OHSS (as a fixed effect) only if it is not a subgroup factor of interest will be used.

### Health economics

Analyses will be conducted in conjunction with a health economic analysis plan (HEAP). Two HEAPs will be produced, one in relation to the interim analysis, and one relating to the end-of-trial analysis.

The primary cost-effectiveness analysis will present cost per hospitalisation avoided. The feasibility of conducting a cost–utility analysis will be explored in the feasibility study, and if viable, the results will also be expressed as incremental cost per quality-adjusted life-years (QALY) gained. An interim health economic analysis will be performed when 65% of the maximum sample size has accrued. The aims of the interim analysis will be to update the pretrial model and to examine the cost-effectiveness of OP compared with UC. The QALY will be calculated using the EQ-5D-5L questionnaire^23^ administered daily until resolution of symptoms and then at 28 days postrandomisation, when women will also be asked to complete resource use questionnaires and a patient cost questionnaire about their last visit to their fertility doctor. Further resource use data will be collected on training of staff and participants on the trial and consumables given; details of monitoring given to participants; outpatient treatment delivery; hospital admission information and treatment received. Unit costs will be derived from appropriate national sources including NHS reference costs and personal social service research unit costs.^24 25^

Analyses will be undertaken from the NHS and personal social service perspective as recommended by the National Institute for Health and Care Excellence^26^ and will follow recommended methods and good practice guides.^27 28^ The primary analysis for both subprotocols within the trial will be a within-trial analysis using data from the trial. A secondary analysis will consider a decision tree model, similar to those used by Casals *et al*, and Csokmay *et al*,^16 29^ that extends costs and outcomes over a 12-month time horizon. Incremental differences between costs and effectiveness/QALYs between those receiving OP and those receiving UC will be described and the incremental cost-effectiveness ratio will be calculated. Sensitivity analyses and subgroup analyses will also be undertaken.

### Qualitative study to aid recruitment

A qualitative study will facilitate the feasibility of conducting the RCT by identifying potential problems with recruitment to the RCT, so that solutions can be instigated rapidly. We will:

1. Identify optimal and suboptimal practice for recruitment to this trial using audio recording of recruitment sessions.
2. Gather information about numbers approached and numbers who consent by assessment of anonymised recruitment logs and other trial documentation.
3. Identify problems with the recruitment process by interviewing recruiting healthcare professionals.
4. Explore the experiences of women being recruited by interviewing both women who consented, and women who declined, to participate in the trial.

### Data management and monitoring

Data management, confidentiality and access, and monitoring were undertaken in line with Sheffield CTRU SOPs. Details can be found in the full study protocol, accessible via the study webpage: (https://www.sheffield.ac.uk/scharr/research/centres/ctru/stop-ohss).

Data access requests will be reviewed by a subcommittee of the TMG while the trial is ongoing and by the Sheffield CTRU in conjunction with project collaborators after the trial has ended.

### Ethics and dissemination

The study is registered on the ISRCTN database (reference: 71978064) and has been approved by the London—Southeast Research Ethics Committee (reference: 22/LO/0015). The findings of this trial will be submitted to peer-reviewed journals and abstracts to national and international conferences. Other stakeholder-specific outputs in relevant formats will be produced for trial participants, commissioners, IVF practitioners, third sector and user advocacy organisations.

### Patient and public involvement

People with experience of OHSS were involved in the design and development of this project. Patient participation was incorporated in the delivery of the project through representation in the TMG and TSC. The Jessop Wing Reproductive Health Public Advisory Panel contributed to the development of the protocol and study materials.

## Supplementary Material

Reviewer comments

Author's manuscript
