# Supplementary material for: Outpatient paracentesis for the management of ovarian hyperstimulation syndrome: study protocol for the STOP-OHSS randomised controlled trial
Source: BMJ Open. 2024 Jan 22;14(1):e076434. doi: 10.1136/bmjopen-2023-076434 (PMC10806818; doi:10.1136/bmjopen-2023-076434)
Supplement: Supplementary data [file bmjopen-2023-076434supp001.pdf]

Research site NHS logo

STOP OHSS

STOP-OHSS (Shaping and Trialling Outpatient Protocols for Ovarian HyperStimulation Syndrome):

A randomised controlled trial to assess the clinical and cost-effectiveness of active management of Ovarian HyperStimulation Syndrome.

PATIENT INFORMATION SHEET

Protocol Number: STH20532
ISRCTN Number: 71978064
Sponsor: Sheffield Teaching Hospitals NHS Foundation Trust
Clinical Research Office Sheffield
Royal Hallamshire Hospital
D Floor
Glossop Road
Sheffield, S10 2JF
Chief Investigator: Mostafa Metwally

Local Principal Investigator: *[insert name]*

Research Nurse: *[insert name]*
Telephone: *[insert]*
Email: *[insert]*

Introduction

We would like to invite you to take part in our research trial. Before you make a decision, it is important for you to understand why the research is being done and what it will involve. Please take time to read the following information carefully and discuss it with friends and family if you wish to do so. Ask us if there is anything that is not clear or if you would like more information. Take time to decide whether or not you wish to take part. Thank you for reading this.

White DA, *et al. BMJ Open* 2024; 14:e076434. doi: 10.1136/bmjopen-2023-076434

The University Of Sheffield.

Research site NHS logo

What is our research about?

Ovarian hyperstimulation syndrome (OHSS) is a condition that can affect women having fertility treatment.

OHSS can be caused by the medication taken to increase egg production during ovulation induction or In Vitro Fertilisation (IVF). The medication known as Follicle-stimulating hormone (FSH) is a hormone which can cause too many eggs to develop, and your ovaries can become large and painful. It can affect women who are still recovering from the effects of this medication given during their fertility treatment and also those who have become pregnant after treatment.

If OHSS occurs up to 7 days after receiving the medication, it is classed as '**Early** OHSS' and if it occurs after 10 or more days, in patients who have become pregnant after treatment, due to their body naturally producing more of the same hormone which can cause the ovaries to become large, is classed as '**Late** OHSS'.

OHSS can range in severity, and can be categorised in the following ways:

- **Mild OHSS** – mild abdominal swelling, discomfort and nausea.
- **Moderate OHSS** – symptoms of mild OHSS, with increased swelling because fluid builds up in the abdomen. This can cause abdominal pain and vomiting.
- **Severe OHSS** – symptoms of moderate OHSS with extreme thirst and dehydration. You may only pass small amounts of urine which is dark in colour and/or you may experience difficulty breathing because of a build-up of fluid in your abdomen. A serious, but rare, complication is a blood clot (thrombosis) forming in the legs or lungs.

Mild symptoms are common and usually resolve quickly, but for some women who experience moderate or severe OHSS these symptoms can worsen. Severe OHSS can lead to serious health complications needing treatment in hospital for several days or weeks, and in a small number of women can be life threatening.

Currently, fertility teams monitor symptoms of OHSS and intervene if the symptoms get worse and the woman needs to be admitted to hospital for intensive monitoring or

White DA, *et al. BMJ Open* 2024; 14:e076434. doi: 10.1136/bmjopen-2023-076434

treatment. This treatment can include a procedure call paracentesis and is normally performed as an inpatient.

The aim of this trial is to understand whether taking action earlier by carrying out the same paracentesis procedure but as an **outpatient** and including additional monitoring can stop symptoms getting worse and prevent women having to be admitted to hospital.

About the procedure

Paracentesis is a procedure where a needle is inserted through the vagina or abdomen to drain the fluid that has collected to relieve the OHSS symptoms and is carried out in a similar way to egg collection. It is generally a safe procedure and will not affect your pregnancy if you are already pregnant. Paracentesis is already regularly carried out at many hospitals for patients who are admitted for treatment. Potential complications include feeling tired, dizzy or lightheaded, increased pain in your tummy, low blood pressure or bleeding from the drainage site but these are short term symptoms and will be managed by your clinical team during the procedure. There is also the possibility of infection and injury to major blood vessels or bowel, but these are extremely rare. We will collect data about these side effects, and they will be monitored by the research team. In this study the procedure will be performed by your doctor/nurse/radiologist in an **outpatient** setting to prevent the OHSS symptoms getting worse. It can feel uncomfortable like egg collection, and you will be given pain relief.

What will happen if I take part in the trial?

Women taking part in the trial will be randomly allocated (randomised) by a computer to participate in one of two groups:

Group A) the intervention group will have the procedure called paracentesis (drainage of fluid) which will take place in an **outpatient** setting. They will have extra monitoring from their fertility team which will include filling in a daily patient diary.

Group B) the control group will not have the paracentesis procedure as an outpatient and will be monitored by their fertility team in the usual way, but will be asked to fill in a daily patient diary.

If you decide to take part this means that neither you nor your doctors can decide which treatment you will have and there is an equal chance of being placed in treatment group A or B.

What happens to you if you are randomised to the intervention group?

Your doctor will perform the paracentesis procedure as an **outpatient**. The procedure involves putting a thin needle or tube into your abdomen or vagina, depending on how your hospital usually does this, to remove the fluid which has built up. You may be offered a local anaesthetic for this procedure and we will ask you to stay in clinic for a few hours after the procedure to make sure you feel well enough to go home. Depending on how much fluid is in your abdomen, your doctor will drain the fluid then take out the needle or insert a tube which will be attached to a small drainage bag to collect the fluid from your abdomen. The bag can then be attached to your leg and hidden discreetly under a skirt, dress or loose fitting trousers and, your team will explain how to empty it once you get home. It will be taken out when no more fluid is draining which can take about 2 weeks, but your doctor will discuss which is best for you before carrying out the procedure.

We will ask you to fill in an electronic daily diary recording any OHSS symptoms you have. This will include your weight, how much fluid you have been drinking, how much urine you have passed, whether or not you have any pain or breathlessness and we will also ask you to measure your abdominal girth (the width of your tummy). We will give you a tape measure, weighing scales and measuring jug to do this. A member of the clinical team from your hospital will call you and discuss this diary information with you until you have recovered from the OHSS. This will be daily for the first few days at least but may then be less often. Depending on your symptoms you may be asked to visit the hospital for review by your doctor to give a blood sample or to have more fluid drained by the same **outpatient** paracentesis procedure.

The trial is designed to monitor your symptoms from home and only bring you into the hospital if necessary. For safety reasons we may ask you to visit the hospital weekly, if necessary, during the first 28 days that you are taking part in the trial or if your symptoms start to get worse or have got better, but again this will be discussed with you.

White DA, *et al. BMJ Open* 2024; 14:e076434. doi: 10.1136/bmjopen-2023-076434

The University Of Sheffield. *Research site NHS logo*

What happens to you if you are randomised to the control group?

Exactly the same will happen as described above but you will not have the paracentesis procedure as an outpatient. However, you may be contacted less frequently by the clinical team, in line with the usual care provided at your centre, this may be every few days, but this will be discussed with you. If your doctor decides based on the information you have given during these calls that you need treatment for your OHSS symptoms, then you will be admitted to hospital as an inpatient. You might still have paracentesis performed but it will be as an inpatient on the ward and not as an outpatient.

Travel expenses for visits to the hospital for trial purposes will be reimbursed (e.g., parking, public transport).

Who is carrying out the research?

This trial is being sponsored (led) by the Sheffield Teaching Hospitals NHS Foundations Trust. *[insert participating site name]* is taking part in the trial as one of the research centres.

Why have I been chosen and what are the possible benefits?

You have been invited to take part in this trial because you have developed moderate or severe OHSS. Taking part in this trial may or may not reduce your chances of going into hospital because of OHSS, but information from this trial may help doctors understand whether it would be helpful to offer this **outpatient** procedure to women who have moderate or severe OHSS in the future.

We plan to recruit 224 women who are suffering from moderate or severe OHSS over 3 years. It will take place in about 20 fertility units in the UK. Half of these women will receive the paracentesis procedure as an **outpatient** and have increased monitoring, and half will not.

Do I have to take part?

No. It is up to you to decide whether or not to take part. If you do decide to take part you will be given this information sheet to keep and will be asked to sign a consent form. If you decide to take part you can still decide to withdraw at any time and without giving a reason.

If you decide to withdraw at any time, or not to take part, this will not affect the standard of care you receive. If you withdraw from the trial, any anonymised information we have

White DA, *et al. BMJ Open* 2024; 14:e076434. doi: 10.1136/bmjopen-2023-076434

already collected about you will be included in the final data analysis, but we will not collect further information without your consent.

Providing information and data as part of the trial

All women, regardless of which group you are in, will be followed up to see how long it takes for your symptoms to get better and whether hospital admission is needed. We will also compare the safety, acceptability and cost of each treatment. We will ask you to complete questionnaires about your health, how you felt about the service you received and any health care you needed 28 days after you gave consent to take part in the trial. These can be completed by post or online. We will also ask you to complete a daily diary as described in the above section "**What happens to you if you are randomised to the intervention group?**".

You will be contacted by telephone by a research nurse/midwife or DR, or asked to come into hospital for the following reasons:

- A weekly visit (up to 28 days or until your symptoms have got better)
- If your symptoms start to get worse
- Once your symptoms have resolved

During these visits, the doctor/ nurse will ask you some questions about your symptoms. Information will also be collected from your medical notes about your symptoms and details of any time spent in hospital because of OHSS.

If you are taken into hospital during the trial at any other NHS Trust, this information may also be requested from them. We will ask your permission to do this.

We would also like to review your medical notes around 3 months (90 days) after you agree to take part in the trial to collect any information about any infections or blood clots (thrombosis) that you may have experienced. At this point we will also collect any information about whether you are pregnant or any pregnancy outcome. If you are not pregnant we will not collect any further data from your notes. If, however, we find that you are pregnant we will review your notes again around 10 months later (13.5 months after you first joined the trial) to collect information about the outcome of your pregnancy.

The University Of Sheffield.

Research site NHS logo

The research team will compare how many people were admitted to hospital from both groups at the end of the trial. This will help us to work out whether early paracentesis as an **outpatient** reduces the chance of women suffering from moderate or severe OHSS being taken into hospital.

White DA, *et al. BMJ Open* 2024; 14:e076434. doi: 10.1136/bmjopen-2023-076434

BMJ Publishing Group Limited (BMJ) disclaims all liability and responsibility arising from any reliance placed on this supplemental material which has been supplied by the author(s)

The University Of Sheffield.

Research site NHS logo

Summary of what will happen if you take part in this trial

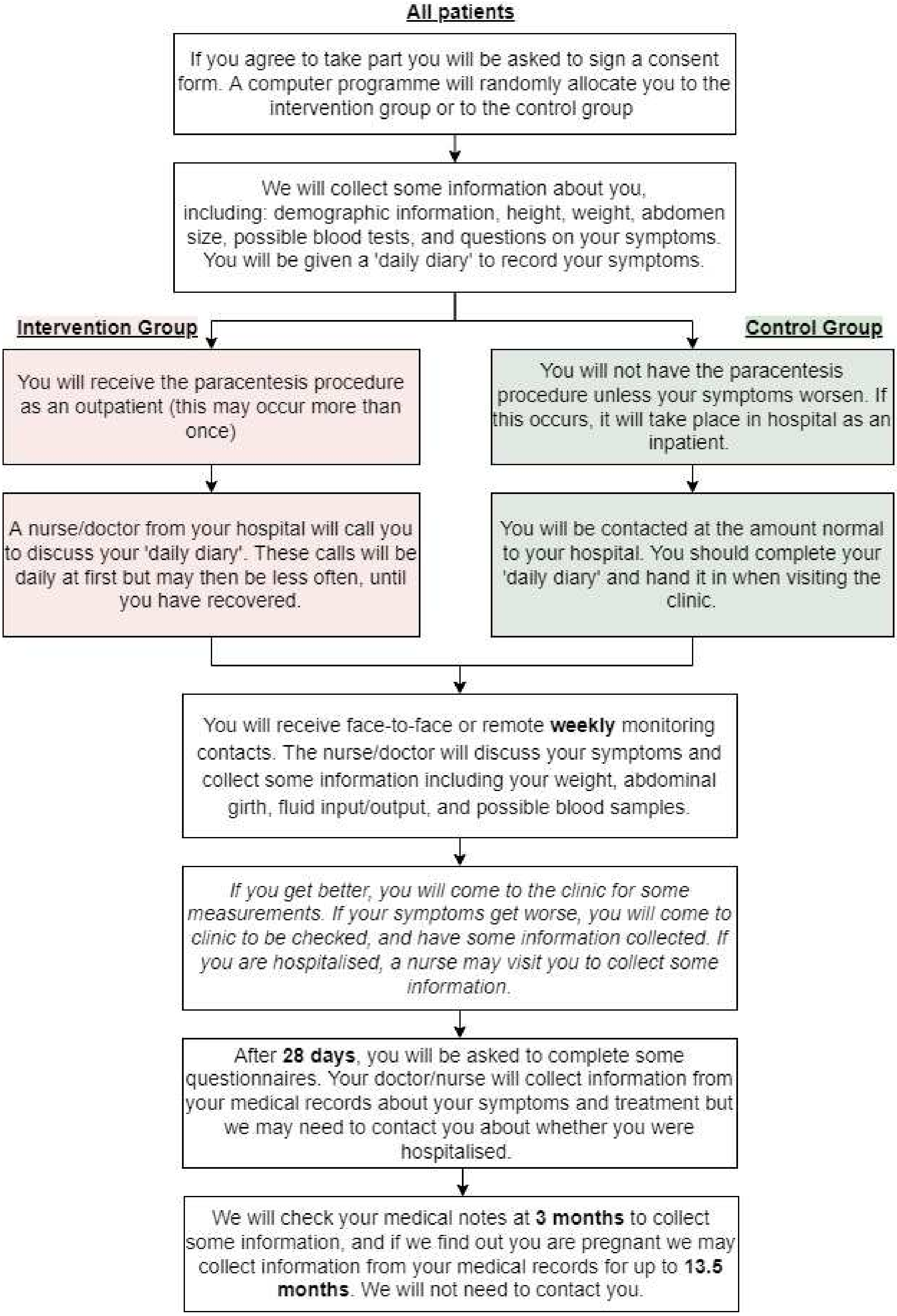

White DA, *et al. BMJ Open* 2024; 14:e076434. doi: 10.1136/bmjopen-2023-076434

The University Of Sheffield.

Research site NHS logo

What are the possible disadvantages and risks of taking part?

Paracentesis is a relatively safe procedure, but there are some risks, which have been described in the section '**About the procedure'**, and these should be discussed with your doctor/ nurse.

Additionally, completing the patient diary daily for 28 days may be an inconvenience to you. This includes measurements of weight, abdominal girth, fluid intake and urine output. For patients in the intervention group, we will use this information to monitor your condition as best as possible. The data that both groups provide in these diaries will help us to answer our research question. We will explain how you should collect the data and provide you with any equipment needed.

How will we use information about you?

Sheffield Teaching Hospitals NHS Foundation Trust (STH NHSFT) is the sponsor that is leading this trial and will act as the data controller. This means that they are responsible for looking after your information and using it properly. The trial is managed by the Clinical Trials Research Unit (CTRU) in the School of Health and Related Research at The University of Sheffield.

Together STH NHSFT and the CTRU will need to use information from you and your medical records for this trial. This information will include your consent to take part in the trial, initials, NHS number (or CHI number if in Scotland), name, contact details and date of birth. STH NHSFT and the CTRU will use this information to do the research or to check your records to make sure that the research is being done properly. Members of the research team who do not need to know who you are will not be able to see your name or contact details. Your data will have a code number instead.

We will keep all information about you safe and secure.

STH NHSFT, the CTRU and *[insert site name]* will keep your data securely for 15 years after the end of the trial.

No one would be able to work out that you took part in the trial from the reports we write about it.

White DA, *et al. BMJ Open* 2024; 14:e076434. doi: 10.1136/bmjopen-2023-076434

The University Of Sheffield.

Research site NHS logo

What are your choices about how your information is used?

You can stop being part of the trial at any time, without giving a reason, but we will keep information about you that we already have, as it may affect our results.

- If you choose to stop taking part in the trial, we would like to continue collecting information about your health from your hospital records. If you do not want this to happen, please let us know and we will stop.
- We need to manage the information that we collect in specific ways for the research to be reliable. This means that we won't be able to let you see or change the data we hold about you.
- If you agree to take part in this trial, you may have the option to take part in future research using your data saved from this trial.
- The anonymised data may be used to support other research in the future, and may be shared with other researchers for comparison studies

Where can you find out more about how your information is used?

You can find out more about how we use your information here www.sheffieldclinicalresearch.org/for-patients-public/how-is-yourinformation-handled-in-research/

What will happen to the results of the research trial?

The results of this trial will be published in scientific journals and presented at scientific meetings, as well as shared directly with NHS Trusts, professional bodies and patient groups. You will not be identified in any report or publication. If you would like to receive a summary of the trial results and a copy of the research report, we will send this to you.

Who is sponsoring and funding the research?

This trial is funded by the National Institute for Health Research (NIHR) Health Technology Assessment (HTA) and sponsored by Sheffield Teaching Hospitals NHS Foundation Trust.

It is organised, managed and coordinated by the University of Sheffield, and data will be collected and stored by this institution.

The University Of Sheffield.

Research site NHS logo

Additionally, the trial will be supervised on a regular basis by a Data Monitoring and Ethics Committee (DMEC) and a Trial Steering Committee (TSC). The primary function of the DMEC is to ensure the absolute safety of all participants.

Who has reviewed the trial?

Research in the NHS is looked at by an independent group of people called a Research Ethics Committee to protect your interests. This trial has been reviewed and given a favourable opinion by the London - South East Research Ethics Committee.

The trial has also been reviewed by the Reproductive Health Research Public Advisory Panel at the Jessop Wing, Sheffield. This is a group of lay members whose main purpose is to ensure that research carried out is patient focused. The role of the panel is to give a patients' perspective on the research that is being carried out.

What if I wish to complain about the way in which this trial has been conducted?

If you have a concern about any aspect of this trial you should ask to speak with the researchers who will do their best to answer your questions. If you remain unhappy and wish to complain formally, you can do this through the NHS complaints procedure, by contacting the Patient Liaison Service (PALs).

Patient Advice and Liaison Service:

Telephone: 0151 702 4353

Email: PALS@lwh.nhs.uk

Harm

Your participation in this trial is to help us find out if performing this procedure in women suffering from moderate or severe OHSS reduces the chance of being admitted to hospital. In the event that something does go wrong, and you are harmed during the research, and this is due to someone's negligence then you may have grounds for a legal action for compensation, but you may have to pay your legal costs. The normal National Health Service complaints procedure will still be available to you.

Further information and contact details for local research team

You are free to ask questions about this information sheet or the trial now or at any time

White DA, *et al. BMJ Open* 2024; 14:e076434. doi: 10.1136/bmjopen-2023-076434

during the trial. If you experience any side effects, or if you have any questions about this research during this trial you may contact: kirsty.mckendrick@sheffield.ac.uk

The trial website contains information that may be helpful. The website is located at www.sheffield.ac.uk/scharr/research/centres/ctru/stop-ohss. If you agree to participate in this trial, you will receive a signed and dated copy of the consent form and this patient information sheet for your records. Whether or not you wish to participate in our trial, we would like to thank you for taking the time to read this information sheet.

The University Of Sheffield.

Research site NHS logo

STOP OHSS

STOP-OHSS (Shaping and Trialling Outpatient Protocols for Ovarian HyperStimulation Syndrome):

A randomized controlled trial to assess the clinical and cost-effectiveness of active management of Ovarian HyperStimulation Syndrome.

CONSENT FORM

Local Principal Investigator: *[insert name]*

Centre Number/ID:

Participant Identification Number:

Please initial

1. I can confirm that I have read and understood the participant information sheet version 3.0 dated 12Dec22 for the STOP-OHSS trial and have had the opportunity to ask questions and these have been answered satisfactorily. ☐ (Your initials)

2. I understand that my participation in the trial is voluntary and I am free to withdraw at any time without my treatment or legal rights being affected. ☐ (Your initials)

3. I understand that my local research team and the study organisers at the University of Sheffield (Clinical Trials Research Unit) will each have access to personal information about my progress and may receive a copy of my consent form for monitoring purposes. Transfer of this information between organisations will be done in confidence via secure email or a secure access controlled database. ☐ (Your initials)

4. I understand that the information collected will be used for medical research only and that I will not be identified in any way in the analysis and reporting of the results. ☐ (Your initials)

5. I understand that even if I withdraw from the trial, information already collected about me may be included in the final analysis after being anonymised. ☐ (Your initials)

White DA, *et al. BMJ Open* 2024; 14:e076434. doi: 10.1136/bmjopen-2023-076434

The University Of Sheffield.

Research site NHS logo

6. I understand that relevant sections of my medical notes and data collected during the trial may be looked at by members of the research team (at Sheffield Teaching Hospitals NHSFT or the Clinical Trials Research Unit at the University of Sheffield), or my participating trial centre, or by individuals from statutory regulators. I give permission for these individuals to have access to my records. (Your initials)

7. I understand that if I am hospitalised during my time in the trial at an NHS Trust or a private centre other than Sheffield Teaching Hospitals NHSFT then the research team will seek to obtain data about the hospitalisation from the other organisation for the purpose of this study. I give my permission for this. (Your initials)

8. I understand that information held by the NHS may be used to keep in touch with me to follow up on my health and pregnancy status for the purpose of the trial. (Your initials)

9. I understand that if I become pregnant during the course of the trial that information about the outcome of my pregnancy including relevant sections of my child's medical notes (if applicable) may be looked at by members of the research team (at Sheffield Teaching Hospitals NHSFT or the Clinical Trials Research Unit at the University of Sheffield) or Sheffield Teaching Hospitals participating trial centre, or by individuals from statutory regulators. I give permission for these individuals to have access to my child's records. (Your initials)

10. I understand that by agreeing to take part in this study I also agree to completing trial visits and follow-up questionnaires. The Sheffield CTRU will be given my contact details in order to send these. (Your initials)

11. I understand that data collected about me without personal identifiers may be used to support other research in the future, and may be shared with other researchers at other institutions to enable them to undertake studies for which my data may be useful; and I give permission for this. (Your initials)

12. I understand the information that I have been given about the above study and I agree to take part. (Your initials)

The following are optional and additional, answering "No" to any will not affect your ability to take part in the study.

The University Of Sheffield.

Research site NHS logo

		Yes		No
13.	I understand that if I choose to stop taking part in the study, the research team would like to continue collecting information about my health from my medical notes, and if applicable those of my child, and I give permission for them to do this.	☐ (Your initials)	OR	☐
14.	I understand I may be contacted in the future to give my consent to take part in future studies similar to this project.	☐ (Your initials)	OR	☐ No
15.	I am happy for a member of the research team to contact me about taking part in an interview about my experience in this trial.	☐ (Your initials)	OR	☐ No

___________________ __________ _______________

Name of participant: Date: Signature:

___________________ __________ _______________

Name of person taking consent: Date: Signature:

When completed: 1 for participant; 1 for researcher site file; 1 to be kept in medical notes

White DA, *et al. BMJ Open* 2024; 14:e076434. doi: 10.1136/bmjopen-2023-076434
