# Supplementary material for: Outpatient paracentesis for the management of ovarian hyperstimulation syndrome: study protocol for the STOP-OHSS randomised controlled trial
Source: BMJ Open. 2024 Jan 22;14(1):e076434. doi: 10.1136/bmjopen-2023-076434 (PMC10806818; doi:10.1136/bmjopen-2023-076434)
Supplement: Supplementary data [file bmjopen-2023-076434supp002.pdf]

STOP-OHSS Simulation Report Final Version 2.0

STOP-OHSS Trial Simulation Report: Summary report on sample size calculation and operating characteristics of the group sequential trial

Munya Dimairo [a], Laura Sutton [b], Tracey Young [c], Ellen Lee [a], Cara Mooney [a], and David White [a]

[a] *Clinical Trial Research Unit (CTRU); Design, Trials and Statistics (DTS), School of Health and Related Research (ScHARR); University of Sheffield*

[b] *DTS, ScHARR, University of Sheffield*

[c] *Health Economics and Decision Science (HEDS), ScHARR, University of Sheffield*

The contribution of the authors is described in Section 10.

White DA, *et al. BMJ Open* 2024; 14:e076434. doi: 10.1136/bmjopen-2023-076434

STOP-OHSS Simulation Report Final Version 2.0

Contents

1 Background 3
2 Findings from a retrospective audit study 3
2.1 Characterisation of the eligible patient population 3
2.2 Estimates of hospitalisation rate in the eligible population 3
2.3 Summary 4
3 Design, trial adaptations and interim decision-making criteria 4
3.1 The design 4
3.2 Trial adaptations and interim analysis aspects 4
3.3 Interim decision-making criteria 5
4 Sample size calculation 5
5 Statistical simulations 6
5.1 Objectives 6
5.2 Approach to simulations 6
6 Results 6
6.1 Summary of sample sizes 6
6.2 Impact of futility thresholds and uncertainty in usual care event rate 7
6.2.1 Impact on the probability of declaring efficacy correctly or in error. 8
6.2.2 Impact on the probability of stopping early for futility correctly or in error. 9
7 Summary 11
8 Limitations 11
9 Impact of early stopping on study duration and sample size saving 12
10 Contributions 12
11 Acknowledgements 12
12 References 12

White DA, *et al. BMJ Open* 2024; 14:e076434. doi: 10.1136/bmjopen-2023-076434

STOP-OHSS Simulation Report Final Version 2.0

1 Background

This report summarises findings from a retrospective review of medical records of patients undergoing in-vitro fertilisation (IVF) treatments that inform the redesign of the STOP-OHSS trial. In addition, the design features of the STOP-OHSS trial, sample size estimates, and the statistical behaviour of the design in addressing the research questions under different assumptions are presented. Finally, the limitations of the design and implications of early stopping on trial duration are also discussed.

2 Findings from a retrospective audit study

This study was undertaken to get a better understanding of the pool of potentially eligible patients with early and late ovarian hyperstimulation syndrome (OHSS) to assess the feasibility of the initially proposed design and recruitment. Importantly, this was also to estimate the OHSS-related hospitalisation rate in the usual care to validate the assumptions made during the design of the original design or use this to modify the design accordingly. Finally, we wanted to understand how the usual care hospitalisation rate differs by proposed study populations (early versus late OHSS) and severity of OHSS (moderate versus severe).

2.1 Characterisation of the eligible patient population

Across five IVF sites, a total of 134 cycles from 134 patients were returned from searches performed over a year (1st March 2019 to 29th February 2020), of which, 63 would have been eligible to be recruited into this trial Of these 63 eligible patients, 25 (39.7%) had early OHSS and 38 (60.3%) had late OHSS. In addition, most patients 73.0% (46/63) were moderate OHSS cases (95% confidence interval, CI: 60.3% to 83.4%) and only 27.0% (17/63) were severe OHSS cases (95% CI: 16.6% to 39.7%).

2.2 Estimates of hospitalisation rate in the eligible population

Of the 63 eligible patients, 26 were hospitalised relating to OHSS, which translated to a 41.2% mean hospitalisation rate across five sites (95% CI: 29.0% to 54.4%).

Only 11 of the 25 eligible patients with early OHSS were hospitalised, representing a mean hospitalisation rate of 44.0% (95% CI: 24.4% to 65.1%). In the late OHSS population, 15 of the 38 eligible patients were hospitalised, translating to a mean hospitalisation rate of 39.5% (95 %CI: 24.0% to 56.6%).

Of the 46 eligible patients with moderate OHSS, 16 (34.8%) were hospitalised (95% CI: 21.4% to 50.2%). In 17 eligible patients with severe OHSS, there was a 58.8% (10/17) hospitalisation rate (95% CI: 32.9% to 81.6%).

White DA, et al. BMJ Open 2024; 14:e076434. doi: 10.1136/bmjopen-2023-076434

2.3 Summary

These estimates are based on OHSS patients from only 5 sites of the expected 20 sites so results should be interpreted with existing uncertainties in mind. However, the estimates give us some insights into eligible patients in the early and late OHSS populations, hospitalisation rates and how this varies by subpopulation and severity of OHSS. Specifically, cases of late OHSS appear to be higher than early OHSS, which is the opposite of what was expected. This strongly suggests that it is unlikely to be feasible to conduct a three-arm definitive trial in the early OHSS population as originally designed to investigate the effectiveness of early outpatient management interventions using paracentesis or a gonadotrophin-releasing hormone (GnRH) antagonist drug compared to usual care. Similarly, the cases of late OHSS seem to be much higher than expected so there is scope for a trial that is more than just exploratory as originally envisaged.

As a result, the Trial Management Group agreed to make changes to the original design by dropping one of the proposed study treatments in the early OHSS population (GnRH antagonist drug) and combining the early and late OHSS populations. Response to treatment in the early and late OHSS population is expected to be similar although the underlying hospitalisation rate may differ as shown in Section 2.2.

3 Design, trial adaptations and interim decision-making criteria

3.1 The design

In an open-label, pragmatic, multi-centre, superiority, parallel-group, group sequential, randomised controlled trial; participants with either early or late OHSS will be individually randomised (1:1) to receive early outpatient paracentesis (OP) or usual care (which excludes early OP). The primary outcome is any OHSS-related hospitalisation for at least 24 hours within 28 days from randomisation.

3.2 Trial adaptations and interim analysis aspects

The goal of the trial is to gather convincing evidence about the effects of the early OP that is most likely to change practice regardless of the direction of results. The recruitment to this trial is expected to be quite challenging so there is a need to pursue the trial only when the study treatment is viewed as promising to use research resources, study patients, and time efficiently. This motivated the use of a group sequential design with an option for early stopping for futility (lack of benefit). The decision to stop for futility is non-binding in the sense that it is advisory so it can be ignored or overruled for some reasons without affecting the statistical behaviour of the design (i.e., it does not increase the type I error beyond the budgeted 2.5% for a one-sided test). One interim analysis will be performed when 65% of the maximum sample size had accrued primary outcome data on hospitalisation within 28 days of randomisation. This delayed timing of interim analysis has been chosen to allow sufficient data to be gathered on hospitalisation rates in both arms to inform reliable interim decisions.

Of note, the trial does not allow for early stopping for overwhelming benefit (efficacy). In addition, the continuation of the trial after interim analysis does not necessarily imply that the study treatment is effective but it only indicates that early OP is promising and worth pursuing further to assess if it is truly effective at the end.

3.3 Interim decision-making criteria

Decision rules at an interim analysis should be fit for purpose and decisions made should be reliable, sensible, and yield convincing and interpretable results. If early OP is found to be effective, the goal is to reduce the hospitalisation rate thus reducing the burden on the NHS, patients, and society, which is cost-saving. To claim the efficacy of early OP, the research team has targeted a realistic and worthwhile 20% reduction in hospitalisation rate that is viewed to be important to influence practice. To inform early stopping, the minimum reduction in hospitalisation below which early OP is viewed as futile needs to be predefined. In addition, a good stopping rule should balance the need to reduce the risk of stopping early for futility of a treatment that is potentially cost-saving and/or effective with the desire to increase the chances of early stopping when treatment is unlikely to be cost-saving and clinically effective.

To help achieve this, a preliminary health economic model which was developed at the grant development stage was updated based on estimated costs associated with early OP and usual care and results from a retrospective audit study (Section 2). This was done under several assumptions about the ratio of early to late OHSS and underlying hospitalisation rates assuming a 20% targeted reduction (if early OP is effective). **Results showed that early OP needs to achieve at least between 4.7% and 5.7% reduction in hospitalisation to become cost-saving** (see appendix found in protocol https://www.isrctn.com/ISRCTN71978064). As such, a sensible futility stopping rule needs to use a value less than 4.7%, which is only 23.5% of the targeted effect of 20% reduction. To further guide the choice of the futility threshold, we explored the statistical behaviour of the design through simulations using futility thresholds (hospital reduction) of 0, -2.5%, -3.0%, -3.5%, -4.0%, and -4.5% (see Section 6.2).

4 Sample size calculation

Sample sizes were estimated using the *rpact* [1] R package without continuity correction under the following assumptions:

- 1:1 allocation ratio, 90% power and 2.5% one-sided type I error rate;
- the best estimate of the usual care hospitalisation rate of 41% (Section 2.2);
- 20% targeted difference in hospitalisation rates between treatment arms;
- one interim analysis at 65% of the maximum total sample size;
- 0% dropout rate;
- futility stopping thresholds of 0, -2.5%, -3.0%, -3.5%, -4.0%, and -4.5%.

STOP-OHSS Simulation Report Final Version 2.0

5 Statistical simulations

5.1 Objectives

Simulations were undertaken to:

1) explore the impact of possible futility thresholds such as on the probability of stopping the trial under a specific assumption about the known treatment effect and the decision-making errors (e.g. incorrectly stopping for futility when the early OP is actually effective);
2) help the research team select an appropriate stopping threshold to use;
3) explore the impact of existing uncertainty in the usual care hospitalisation rate (which can vary from 29% to 54%) on study power.

5.2 Approach to simulations

Simulations were performed as follows in that order:

1. use the calculated 224 maximum total sample size (112 per arm) corresponding to a futility threshold of -4.5% (reduction in hospitalisation);
2. generate pseudo-trial data with the number of patients calculated as in item 1 using a binomial distribution assuming a certain treatment effect (differences in proportions of 0%, 5%, 10%, 15%, and 20% were used) and underlying usual care event rate (hospitalisation rate of 29% to 50% with an increment of 3% were used);
3. for each scenario, repeat the process to generate 100 000 trials sufficient to produce accurate results;
4. for each simulated pseudo-trial, analyse interim data from the first 65% of participants and decide whether the trial should have been stopped at this point using a selected futility threshold. This was repeated for futility thresholds of 0, -2.5%, -3.0%, -3.5%, -4.0%, and -4.5% (hospitalisation reduction);
5. for each simulated pseudo-trial, what the overall decision about clinical efficacy would have been if it was not stopped early was noted;
6. using data from all 100 000 simulated pseudo-trials, the proportions of trials that would have been stopped for futility and those that indicated efficacy if they were not stopped early were calculated.

All simulations were performed in Stata v16.1 using a bespoke program and results are presented in Section 6.2.

6 Results

6.1 Summary of sample sizes

Table 1 summarises the total maximum sample sizes and total sample sizes required for interim analysis under different assumptions. For example (row marked in green), the trial

will require a maximum total sample size of ~224 (112 per arm), rounded upwards to the nearest even number. An interim analysis will be performed when ~146 participants (73 per arm) had accrued primary outcome data on hospitalisation within 28 days of randomisation. This assumes a 90% power, one-sided 2.5% type 1 error rate, 41% hospitalisation rate in the usual care arm (see Section 2.2), a 20% worthwhile and realistic reduction in hospitalisation rate (as per original design), and a futility threshold of -4.5% (difference in hospitalisation rate). In this case, a trial will be stopped early for futility if the observed reduction in hospitalisation rate at an interim analysis is less than 4.5% (equivalent to a one-sided p-value above 2.9 or critical value below 0.553). There is only a **1.9% chance** of stopping the trial early for futility in error for a beneficial treatment which is very low as expected.

As noted in Table 1, the total sample size is very similar across futility thresholds (0% to 4.5%). However, the influence of the size of the futility threshold on the chances of stopping early is apparent in Figure 2.

Table 1. Estimated total sample sizes assuming 90% power and 2.5% one-sided type I error.

Usual care event rate	A targeted difference in hospitalisation rates	Futility stopping boundaries at interim analysis			Efficacy boundaries at:		Sample sizes across groups		Probability of early stopping when paracentesis is efficacious
		Critical value	Difference in proportions (paracentesis – usual care)	One-sided p-value	An interim analysis	The final analysis	Interim analysis (65% of the total)	In total	
41%	-20%	0	0	0.50	N/A	-0.126	144	221	0.0045
41%	-20%	0.305	-0.025	0.38	N/A	-0.125	144	222	0.0104
41%	-20%	0.366	-0.030	0.357	N/A	-0.125	145	222	0.0121
41%	-20%	0.429	-0.035	0.334	N/A	-0.125	145	222	0.0142
41%	-20%	0.491	-0.040	0.312	N/A	-0.125	145	223	0.0165
41%	-20%	0.553	-0.045	0.290	N/A	-0.125	145	223	0.0191

Assumptions: 1:1 randomisation; 0% dropout rate; interim analysis to be performed when 65% of the maximum total sample size had accrued outcome data; NA, not applicable.

6.2 Impact of futility thresholds and uncertainty in usual care event rate

A good trial design should facilitate reliable decisions with high probability both at interim analysis and at the end if it progressed beyond interim analysis. For example, if we simulate many pseudo trials assuming that a treatment is effective with a targeted treatment effect we wish to see (e.g., 20%), a very high proportion of these trials should progress after interim analysis and the treatment should be deemed efficacious in the end. On the contrary, if we simulate assuming that a treatment does not work (0% effect say), we should expect most trials to indicate early stopping and only a negligible fraction to indicate efficacy in the end (i.e., making erroneous conclusion claiming efficacy, type I error).

White DA, et al. BMJ Open 2024; 14:e076434. doi: 10.1136/bmjopen-2023-076434

6.2.1 Impact on the probability of declaring efficacy correctly or in error.

As evident in Figure 1, across all presented scenarios, the choice of futility threshold between -4.5% and 0 has a negligible effect on the chances of declaring efficacy either correctly or incorrectly.

Figure 1 (**A**) shows the power of the study as the usual care hospitalisation rate changes from 29% to 50%. If the underlying hospital reduction is as assumed (20%), the study will still have more than 87% power even if the usual care hospitalisation rate is as high as 50%. That is, regardless of the usual care hospitalisation rate, the study will have sufficient power (above or close to the assumed 90%) to find a 20% hospital reduction if the treatment is truly effective with this effect.

On the contrary, in Figure 1 (**B**), if the observed hospital reduction is 15% (rather than the assumed 20%), the power of the study will range from 62% to 79% to find a 15% hospital reduction if early OP is effective with this effect as the usual care hospitalisation rate decreases from 50% to 29%, respectively. Figure 1 (**C**) shows that the study will be severely underpowered to find a 10% hospital reduction across all scenarios of the usual care hospitalisation rate even though this effect is likely to be cost-saving based on the updated preliminary health economic model.

Figure 1 (**E**) shows the chances of erroneously claiming that early OP is effective when its hospitalisation rate is the same as in the usual care (i.e., 0% hospital reduction). This error is very small as expected and ranges from 2.4% to 2.6% across (accounting for Monte Carlo error) all scenarios compared to the targeted 2.5% for a one-sided test.

In summary, if the underlying treatment effect is at least 20% hospital reduction, the study will be sufficiently powered across all realistic scenarios of the usual care hospitalisation rate and this is not affected by the choice of the futility threshold of between -4.5% and 0%. In addition, the chance of incorrectly declaring efficacy when treatment is ineffective is very small and as expected. However, the study will be severely underpowered for effects that are far less than 20% but likely to be cost-saving (e.g., 5% to 15%).

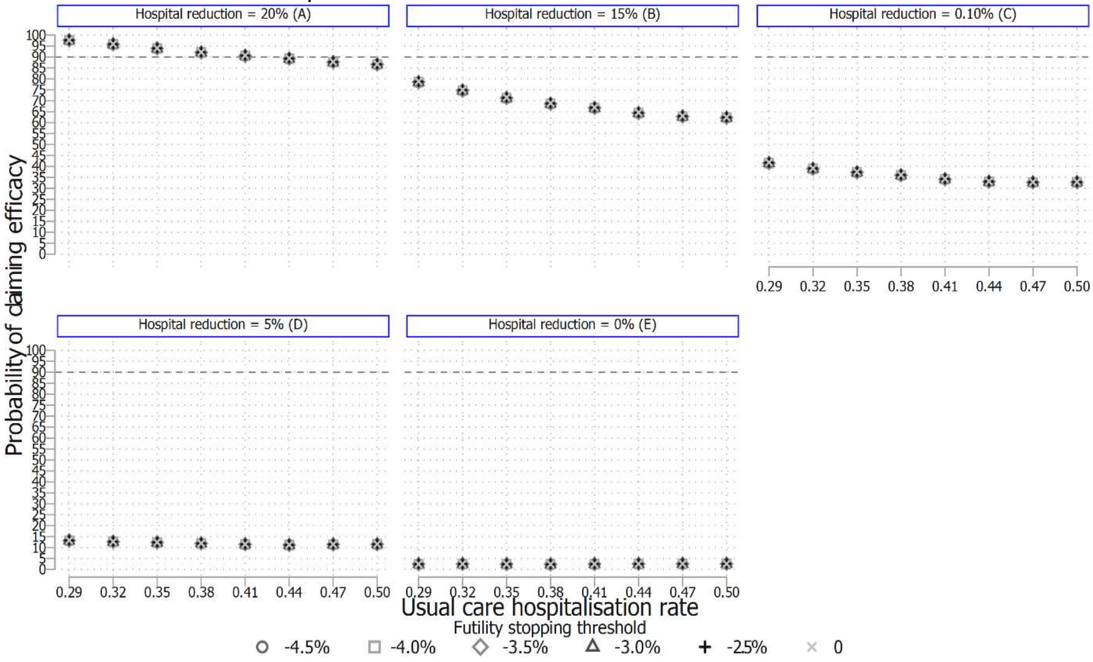

Figure 1. Impact on the chances of claiming efficacy at the end of the trial.

6.2.2 Impact on the probability of stopping early for futility correctly or in error.

Figure 2 presents the chances of stopping early for futility under different assumptions of the underlying treatment effect, usual care hospitalisation rate, and futility stopping threshold. As evident, the impact of futility stopping threshold becomes more apparent as the underlying hospital reduction changes from 20% to 0%. In principle, we want a design that:

1) allows early stopping with high probability when the underlying treatment effect is in support of the null hypothesis of no difference and,
2) erroneously leads to trial early stopping with very low or negligible probability when it is known that the treatment is beneficial.

In Figure 2 (**A**), if early OP is effective, the chances of stopping early range from 0% to 2.9% as the futility stopping threshold decreases from 0 to -4.5% and the usual care hospitalisation rate increases from 29% to 50%, respectively. For example, if the underlying treatment effect is 20% as hoped and the usual care event rate is 41% (current best estimate), there is only approximately a 2.2% chance of stopping early for futility if a stopping threshold of -4.5% is used.

Figure 2 **(E)** is a scenario when it is known that the treatment effect is **0%** and a good design should trigger early stopping with high probability. As evident, this is achieved across all scenarios when a futility stopping threshold of -4.5% is chosen. For example, this probability is 72.2% when the usual care hospital rate is 41% (current best estimate).

Figure 2 (subfigures **B-D**) are interpreted in the same manner when the underlying treatment effect is 15%, 10%, and 5% respectively.

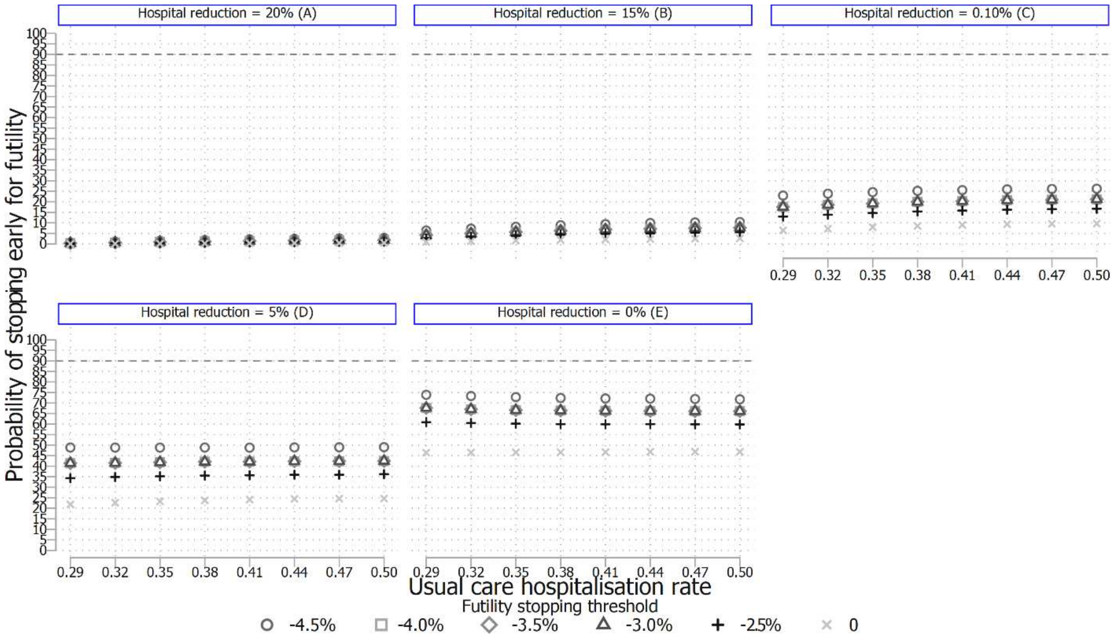

Figure 2. Impact on the chances of stopping early correctly or in error.

In summary, when choosing a futility stopping threshold, there is a trade-off between increasing the chances of early stopping for an ineffective treatment (scenario **E**) and reducing the risks of stopping a trial early for futility erroneously (scenario **A**).

7 Summary

The trial will require a maximum total of 224 participants (112 per arm) to maintain a power of 90% for a 2.5% one-sided assuming a 41% hospitalisation rate in the usual care and a large targeted reduction of 20%. One interim analysis will be performed to inform whether the trial should be stopped early for futility when 65% of the maximum total sample size had accrued primary outcome data. It has been shown that the trial will be sufficiently powered across realistic event rates (e.g., 29% to 50%) as long as the targeted effect of a 20% reduction is realistic. Key limitations of the design are highlighted in Section 8.

In this trial, it has been shown in Figure 1 that the choice of futility stopping threshold had a negligible effect on the power and type I error rate of the study across the usual care event rate considered. However, as evident in Figure 2, the choice of futility stopping threshold has a significant effect on the chances of correctly stopping for futility and a very small effect on the probability of erroneously stopping early for futility.

The choice of a **4.5% hospital reduction as a futility stopping threshold appears very reasonable** for four specific reasons. First, it is below the lower bound of hospital reduction likely to result in cost savings. Second, it gives a higher chance of stopping the trial early when then the treatment is known to be ineffective which is the motivation for futility early stopping. Third, the risk of erroneously stopping the trial early for futility when the treatment is known to be effective is very small. Finally, it does not affect the power of the study.

The final decision on which futility stopping threshold to use lies with the research team guided by the results presented and the levels of risk they are willing to tolerate. Notably, in this design, the chosen futility stopping threshold is advisory so it can be overruled or ignored by the independent data monitoring committee and research team without affecting the design negatively.

Finally, the group sequential design considered here is feasible to implement and the primary outcome is observed within 28 days of randomisation and recruitment in this population is expected to be very slow.

8 Limitations

One major limitation of the design is that it is based on a large treatment effect of 20% hospital reduction which is viewed as realistic (based on data from observational studies) and worthwhile to change practice. However, as noted from the updated health economic model, hospital reductions of about 4.7% to 5.7% are also likely to result in cost savings. As a result, there is a region where the treatment effect is likely to be cost-saving (e.g., 10% to 15%) but the trial will be severely underpowered for such small to moderate treatment effects. This will complicate the interpretation of results if such effects turn out to be true. Finally, the treatment effect of 20% becomes unrealistic and impossible to achieve as the usual care event rate gets closer to 20%, however, the hospitalisation rate in the usual care is likely to be much higher than 20% based on the current best estimate presented in Section 2.2.

9 Impact of early stopping on study duration and sample size saving

An audit review of medical records suggested that the average recruitment across 20 sites will be around 4.6 patients per month. Stopping the trial early for futility at an interim analysis implies that an additional total of 78 participants would no longer be recruited. The trial will not be paused during interim analysis and interim decision-making process as the recruitment rate is expected to be very slow. As such, even if the trial is stopped early, we would expect negligible participants to have been recruited during interim analysis (e.g., at most 2 participants per arm). At this point, if we assume a constant average recruitment rate of 4.6 patients per month across all sites and staggered opening of sites to recruitment starting with 2 followed by 5, 8, 11, 15, 18, and 20 in consecutive months, then 10 months of further recruitment will no longer be required. Finally, potential cost savings can be extrapolated under different scenarios.

10 Contributions

Munya Dimairo designed the simulation study, wrote the simulation code, conducted simulations, and drafted the simulation report.

Laura Sutton performed statistical quality control in R relating to results presented in Table 1.

Ellen Lee performed statistical quality control in Stata relating to the simulation code.

Tracey Young contributed to the preliminary health economic model that informed the futlity stopping threshold.

David White and Cara Mooney reviewed the report and contributed to Section 9.

11 Acknowledgements

We are very grateful for the invaluable feedback from the Trial Steering Committee independent statistician (Babak Choodari-Oskooei, Medical Research Council (MRC) Clinical Trial Unit (CTU) at University College London (UCL)). Finally, we would like to thank the Trial Management Group (TMG) responsible for the day-to-day conduct of the trial.

12 References

1. Wassmer, G. & Pahlke, F. RPACT Package Overview | RPACT.
