# Supplementary material for: Outpatient paracentesis for the management of ovarian hyperstimulation syndrome: study protocol for the STOP-OHSS randomised controlled trial
Source: BMJ Open. 2024 Jan 22;14(1):e076434. doi: 10.1136/bmjopen-2023-076434 (PMC10806818; doi:10.1136/bmjopen-2023-076434)
Supplement: Author's manuscript [file bmjopen-2023-076434.draft_revisions.pdf]

BMJ Open

BMJ Open is committed to open peer review. As part of this commitment we make the peer review history of every article we publish publicly available.

When an article is published we post the peer reviewers' comments and the authors' responses online. We also post the versions of the paper that were used during peer review. These are the versions that the peer review comments apply to.

The versions of the paper that follow are the versions that were submitted during the peer review process. They are not the versions of record or the final published versions. They should not be cited or distributed as the published version of this manuscript.

BMJ Open is an open access journal and the full, final, typeset and author-corrected version of record of the manuscript is available on our site with no access controls, subscription charges or pay-per-view fees (http://bmjopen.bmj.com).

If you have any questions on BMJ Open's open peer review process please email info.bmjopen@bmj.com

BMJ Open

Outpatient paracentesis for the management of Ovarian HyperStimulation Syndrome: Study protocol for the STOP-OHSS randomised controlled trial

Journal:	*BMJ Open*
Manuscript ID	bmjopen-2023-076434
Article Type:	Protocol
Date Submitted by the Author:	07-Jun-2023
Complete List of Authors:	White, David; The University of Sheffield, Clinical Trials Research Unit, School of Health and Related Research<br>Pye, Clare; Sheffield Teaching Hospitals NHS Foundation Trust, Jessop Wing<br>Ridsdale, Katie; The University of Sheffield, Clinical Trials Research Unit, School of Health and Related Research<br>Dimairo, Munyaradzi; The University of Sheffield, Clinical Trials Research Unit, School of Health and Related Research<br>Mooney, Cara; The University of Sheffield, Clinical Trials Research Unit, School of Health and Related Research<br>Wright, Jesssica; The University of Sheffield, Clinical Trials Research Unit, School of Health and Related Research<br>Young, Tracey; The University of Sheffield, School of Health and Related Research<br>Cheong, Ying; University of Southampton, Obstetrics and Gynaecology<br>Drakeley, Andrew; Liverpool Women's NHS Foundation Trust, Hewitt Fertility Centre<br>Mathur, Raj; Manchester University NHS Foundation Trust, Manchester Academic Health Sciences Centre<br>O'Cathain, Alicia; University of Sheffield, School of Health and Related Research<br>Desoysa, Lauren; The University of Sheffield, Clinical Trials Research Unit, School of Health and Related Research<br>Sizer, Anya; Fertility Network UK<br>Lumley, Elizabeth; The University of Sheffield<br>Chatters, Robin; The University of Sheffield, Clinical Trials Research Unit, School of Health and Related Research<br>Metwally, Mostafa; Sheffield Teaching Hospitals NHS Foundation Trust; The University of Sheffield
Keywords:	Reproductive medicine < GYNAECOLOGY, GYNAECOLOGY, REPRODUCTIVE MEDICINE

SCHOLARONE™
Manuscripts

Title:

Outpatient paracentesis for the management of Ovarian HyperStimulation Syndrome: Study protocol for the STOP-OHSS randomised controlled trial

David White[a], Clare Pye[b], Katie Ridsdale[a], Munya Dimairo[a], Cara Mooney[a], Jesssica Wright[a], Tracey Young[c], Ying Cheong[d], Andrew Drakeley[e], Raj Mathur[f], Alicia O'Cathain[c], Lauren Desoysa[a], Anya Sizer[g], Elizabeth Lumley[c], Robin Chatters[a], Mostafa Metwally[b,c]

a – Clinical Trials Research Unit, School of Health and Related Research, The University of Sheffield
b – Jessop Fertility, Sheffield Teaching Hospitals NHS Foundation Trust
c – School of Health and Related Research, The University of Sheffield
d – Complete Fertility Southampton, Princess Anne Hospital, Human Development and Health, Faculty of Medicine, University of Southampton
e – Hewitt Fertility Centre, Liverpool Women's NHS Foundation Trust
f – Manchester Academic Health Sciences Centre, Manchester Foundation Trust
g - Fertility Network UK

Corresponding author

Mr David White
Clinical Trials Research Unit (CTRU)
School of Health and Related Research (ScHARR)
The University of Sheffield
Email: d.a.white@sheffield.ac.uk

Sponsor

Sheffield Teaching Hospital NHS Foundation Trust
The University of Sheffield
D Floor, Royal Hallamshire Hospital
Glossop Road
Sheffield
S10 2JF

Key words

Assisted conception
Randomised Controlled trial
Ovarian HyperStimulation Syndrome
Paracentesis
In Vitro Fertilisation

Protocol Version 4 Dated 12 January 2023

Word count: 4347

For peer review only - http://bmjopen.bmj.com/site/about/guidelines.xhtml

Abstract

Introduction

Ovarian hyperstimulation syndrome (OHSS) is the most significant short-term complication of pharmacological ovarian stimulation. Symptoms range from mild abdominal discomfort to rare complications such as renal failure, thromboembolism, and respiratory distress syndrome.

Currently, clinical practice typically involves monitoring the patient until the condition becomes severe, at which point they are admitted to hospital, where drainage of ascitic fluid (paracentesis) may take place. Preliminary studies have indicated that earlier outpatient paracentesis may reduce the progression of OHSS and prevent hospitalisation in women.

Methods and analysis

This United Kingdom, multi-centre, pragmatic, two-arm, parallel-group, adaptive (group sequential with one interim analysis), open-label, superiority, confirmatory, group sequential, individually randomised controlled trial, with internal pilot will assess the clinical and cost-effectiveness and safety of outpatient paracentesis versus conservative management (usual care) for moderate or severe OHSS. 224 women from 20 National Health Service & Private fertility units will be randomised (1:1) and followed up for up to 13.5 months. The primary outcome is the rate of OHSS related hospital admission of at least 24 hours within 28 days post-randomisation. The primary analysis will be intention to treat with difference in hospitalisation rates as measure of treatment effect. Secondary outcomes include time to resolution of symptoms, patient satisfaction, adverse events and cost-effectiveness. A qualitative sub-study will facilitate the feasibility of recruitment. Participant recruitment commenced in June 2022.

Ethics & Dissemination

London - Southeast Research Ethics Committee approved the protocol (reference: 22/LO/0015). Findings will be submitted to peer- reviewed journals and abstracts to

For peer review only - http://bmjopen.bmj.com/site/about/guidelines.xhtml

relevant national and international conferences, as well as being disseminated to trial participants and patient groups.

Trial Registration number: ISRCTN - 71978064.

Strengths and Limitations of this study

- This is the largest randomised controlled trial to date which aims to establish whether outpatient paracentesis reduces the rate of OHSS related hospital admissions in those presenting with moderate or severe OHSS, compared to usual care.
- Due to the nature of the intervention, it is not possible to blind study participants or clinicians.
- Measures are taken to reduce the impact of this, including a secondary independent blinded adjudication of hospital admissions and blinding of trial statisticians
- Patients with lived experience were involved in the design of the trial and will provide advice throughout delivery

For peer review only

Introduction

Ovarian hyperstimulation syndrome (OHSS) is the most significant short-term complications of pharmacological ovarian stimulation for assisted conception. It is most often seen in women with a high ovarian reserve, who are prone to an excessive response to gonadotropin stimulation.

There is no universally accepted classification of OHSS. In the UK, the Royal College of Obstetricians and Gynaecologists (RCOG) classification is widely used and includes mild, moderate, severe, or critical categories[3]. Moderate cases involve the build-up of fluid in the abdomen (called ascites), increased ovarian size, and abdominal distention and discomfort. In severe cases, fluid retention and dehydration can lead to changes to the constitution of the blood (haemoconcentration and hypoalbuminemia); critical cases can lead to respiratory distress, thrombosis, disturbed renal and liver functions, and rarely death [1].

For peer review only - http://bmjopen.bmj.com/site/about/guidelines.xhtml

OHSS can be further classified into early and late (Table 1). Early OHSS is usually caused by the ovarian stimulation drugs given during treatment and usually occurs within 7 days after the final drug Human chorionic gonadotropin (hCG) is given. Late OHSS usually occurs 10 days or more after the administration of hCG and is caused by endogenous hCG of the resulting pregnancy. The late type is usually more difficult to control, runs a longer course and is more severe [2].

Table 1. Categorisation of OHSS and its severity, based on the RCOG definitions[3]

**Categorisation of OHSS**	
Early OHSS	Caused by the ovarian stimulation drugs given during Assisted Reproductive Technologies and occurs usually up to 7 days of the final trigger drug (hCG) being given.
Late OHSS	Usually occurs 10 or more days after the trigger drug is given.
Where a patient presents at day 8 or 9 clinical judgement will be used to classify the early or late OHSS.	

**Severity of OHSS**	
Mild OHSS	• Abdominal bloating<br>• Mild abdominal pain<br>• Ovarian size is usually below 8cm
Moderate OHSS	Patients do not meet the criteria of severe (described below) and have fluid accumulation in abdomen (ascites), confirmed by ultrasound scan. The following symptoms may also be present:<br>• Moderate abdominal pain<br>• Increased ovarian size of usually 8-12cm<br>• Nausea with or without vomiting<br><br>*Some degree of clinical judgement may be needed to confirm the diagnosis of moderate OHSS.*
Severe OHSS	Patients have fluid accumulation in abdomen (clinical ascites/clinically detectable fluids), confirmed by ultrasound, with or without hydrothorax<br>The main distinguishing features are any of the following:<br>• Low urine output (oliguria)(< 300 ml/day or < 30 ml/hour)<br>• Haematocrit >0.45, (confirmed via full blood count (FBC) test)<br>• Ovarian size >12cm.
Critical OHSS	Clinically obvious ascites, with one of the following features:<br>the patient has a tense ascites or a large hydrothorax<br>• Haematocrit level >0.55 (confirmed via FBC test)**<br>• A white cell count of over 25000/ml (confirmed via FBC test)<br>• Anuria (very little/no urine) (< 100 ml/day)<br>• Thromboembolism<br>• Acute respiratory distress syndrome<br><br>*** haematocrit level is the most important measurement*

For peer review only

Clinical practice usually involves monitoring the patient until the condition becomes severe, when the patient is admitted to hospital, as recommended by the RCOG guideline [3]. Inpatient management often includes drainage of ascitic fluid (paracentesis) which can result in a

significant improvement of the condition and in renal blood fluid, urine output, and reversal of the haematological abnormalities [4–6]. Complications such as venous thromboembolism can have long-term health problems lasting well beyond the length of the pregnancy.

Although mild forms are fairly common (approximately 1 in 3 women undergoing IVF (In Vitro Fertilisation) treatment), more severe OHSS (up to 8% for combined moderate and severe OHSS) [3] can have a significant impact on a woman's health resulting in prolonged hospitalisation and posing a significant economic burden on both patient and National Health Service (NHS). With over 69,000 IVF cycles performed in the United Kingdom (UK) in 2019 alone[7], the burden of the problem becomes evident [3]. It is therefore important to look into novel approaches to improve outcomes and the management of complications associated with IVF [8].

Small retrospective studies [9,10] and a larger uncontrolled study [11] have suggested that transvaginal paracentesis can reduce the need for hospitalisation to between 2.9% and 14% of patients. Another small cohort study identified patients with moderate OHSS at risk of this progressing to severe OHSS and reduced this progression with only 1 to 3 paracentesis procedures [12]. There is also some evidence to suggest transabdominal administration of paracentesis can prevent inpatient hospitalisation compared to those managed supportively [13]. Furthermore, preliminary studies have safely and effectively managed patients in the outpatient setting by using a pigtail catheter to drain ascetic fluid [14,15]. All of these studies have still achieved high pregnancy rates of 68% [13] and 100% [12]. Modelling also suggests that it would be more cost-effective to treat patients with moderate or severe OHSS with early outpatient paracentesis (OP), compared to less active management and inpatient admission [16].

Preliminary results have been encouraging and suggest promising safety and effectiveness of OP [9–13,15,17]. However, this needs to be evaluated in a robust and adequately powered randomised controlled trial (RCT) taking into consideration variabilities in practice across fertility units.

For peer review only - http://bmjopen.bmj.com/site/about/guidelines.xhtml

This study aims to establish the clinical and cost-effectiveness, safety, and acceptability of OP as an active management for women with moderate or severe OHSS. The primary objective is to establish whether OP reduces the rate of OHSS related hospital admissions in those presenting with moderate or severe OHSS compared to usual care. Secondary objectives are to:

1) Establish whether OP prevents the escalation of OHSS severity;
2) Establish whether OP reduces the time taken for OHSS symptoms to resolve;
3) Establish the safety of OP as an active intervention for moderate or severe OHSS;
4) Explore whether OP would improve patient satisfaction and quality of life;
5) Establish whether OP is cost-effective by examining healthcare resource use and patient costs;
6) Facilitate the feasibility of conducting the RCT by identifying problems with the conduct of the RCT during an internal pilot.

Pre-trial development work involved qualitative interviews with clinicians and patients exploring the feasibility and acceptability of earlier active management protocols. These results fed into a consensus event to confirm the treatment protocol used in the RCT. We also undertook a comprehensive audit of proposed RCT centres to inform the parameters used to design the RCT.

Method and analysis

Full detailed methods of the STOP OHSS trial are included in the trial protocol, available via the trial registry (https://www.isrctn.com/ISRCTN71978064).

STOP-OHSS is a pragmatic, parallel open-label, multi-centre, superiority, adaptive, group sequential, confirmatory RCT with an internal pilot (after 15 of 31 months recruitment) to assess feasibility aspects (Figure 1). Eligible participants will be individually randomised (1:1) to receive either routine treatment or OP plus daily diary monitoring.

The trial is a collaboration between The Jessop Wing, Sheffield Teaching Hospital NHS Foundation Trust & the University of Sheffield - Clinical Trials Research Unit (CTRU) who are responsible for the conduct of the trial. It is funded by the National Institute of Health and

For peer review only - http://bmjopen.bmj.com/site/about/guidelines.xhtml

Care Research (NIHR) Health Technology Assessment (HTA), who had no involvement in the study design or conduct; or the decision to submit the protocol for publication. The trial will be conducted in compliance with the protocol, Good Clinical Practice and regulatory requirements. Substantial protocol amendments will be communicated to all relevant bodies. Main trial recruitment commenced June 2022 and is ongoing.

Internal pilot

An internal pilot will use the same procedures as the main trial and will assess the following after 15 months of a planned 31-month recruitment period:

1) Participant recruitment (both arms),
2) Retention of randomised participants (both arms),
3) Delivery of the OP treatment (only OP arm).

At the end of the pilot phase, the Trial Steering Committee (TSC) will report to the funder on whether criteria have been met to continue.

Recruitment

The trial will recruit women attending approximately 20 NHS or private UK Fertility Units, undergoing Assisted Reproductive Technologies (including IVF, IntraCytoplasmic Sperm Injection and Intrauterine Insemination) and who experience moderate or severe, early, or late OHSS symptoms. Participation is voluntary and choosing not to participate will not negatively influence the woman's treatment. Consent can be withdrawn at any stage. Women will be identified by the following recruitment strategies.

Monitoring cases – women who are considered at risk of developing moderate or severe OHSS due to various risk factors such as a previous episode of OHSS, high response to stimulation with increased follicular activity, >15 eggs with or without symptoms of OHSS, polycystic ovary syndrome, high levels of Anti-Müllerian hormone (greater than 30 pmol/l, younger women (<30yrs) who have a good ovarian reserve and are therefore known to respond well to treatment, low body mass index or any symptoms of OHSS like bloating and nausea. Incident cases – women with moderate or severe OHSS who were not previously identified as at risk.

For peer review only - http://bmjopen.bmj.com/site/about/guidelines.xhtml

Potentially eligible participants will be approached as soon as possible after the diagnosis of moderate or severe OHSS and, where possible, initial eligibility screening has taken place. Women may also be alerted to the trial via the trial website or posters displayed at the fertility unit.

Research staff will provide the patient with the participant information sheet and/or link to a trial animation video outlining the study as soon as they become aware the woman is potentially eligible. Prior to randomisation, full written informed consent (see supplementary material, v3 dated 12th Dec 2022) will be obtained by a suitably trained clinician researcher at the clinic visit. Standard consent includes a provision for anonymised data to be shared with other researchers. Baseline data will be collected at this visit and participants will be randomised.

Women will be considered suitable if they meet the following eligibility criteria.
Inclusion criteria:

1. Women presenting with moderate or severe, early or late OHSS,
2. Patients able and willing to attend weekly follow-up appointments in person or remotely, daily remote appointments/phone calls, and able to undertake self-monitoring at home.

Exclusion criteria:

1. OHSS-related exclusion criteria:
    a. Significant pain or vomiting requiring hospitalisation,
    b. Pulmonary embolism,
    c. When in the judgment of the clinician, the patient's condition is severe enough to warrant admission to a High Dependency Care Unit (such as Critical OHSS as defined in the RCOG green-top guidelines) and therefore not suitable for outpatient management.
2. Non-OHSS related medical conditions: a concurrent medical condition requiring immediate inpatient management,

For peer review only - http://bmjopen.bmj.com/site/about/guidelines.xhtml

3. Patients who have been previously randomised but later present with moderate or severe OHSS symptoms in subsequent cycles after their initial trial involvement,
4. Participation in other trials involving ovarian stimulation or ovarian response.

Centralised Research Nursing Delivery Team

Local research teams will be supported in recruiting participants and ongoing management of the trial protocol procedures from the Central Research Nursing Delivery team based at the Jessop Wing, Sheffield Teaching Hospitals NHSFT. This team will support local discussions with potential participants about trial participation, randomisation procedures, and monitoring and routine follow up visits in both trial arms.

Randomisation and concealment

Patients will be randomised using a centralised web-based system (SCRAM) hosted by Sheffield CTRU, which has user-restricted functionalities granting access rights to specific areas as appropriate. The sequence will be computer generated using permuted block randomisation stratified by recruiting site and severity of OHSS. Block sizes will be disclosed during dissemination of findings and only the randomisation (trial) statistician(s) not involved in the recruitment process will know the block sizes during the trial. The trial statistician will generate the randomisation sequence; however, they will not have access to the generated sequence. Research staff at recruiting centres will use a web-based computer system with restricted access rights to enter participant details; randomisation outcome will then be revealed. Re-randomisation will not be permitted.

Intervention and control

Following randomisation, women in the intervention arm will have OP performed in the outpatient setting as soon as clinically possible. Sites will follow their own local procedures for conducting paracentesis either abdominally or vaginally by a suitably trained Doctor, Advanced Nurse Practitioner or Radiologist in the fertility, gynaecology, or radiology unit and while the trial does not dictate how to perform the intervention, guidance will be offered.

If re-hydration is required via intravenous methods this will be at the discretion of the attending clinician. If a large volume of fluid is removed, then intravenous colloid therapy should be considered. If this administration of fluid results in overnight inpatient

For peer review only - http://bmjopen.bmj.com/site/about/guidelines.xhtml

hospitalisation it will not be viewed as meeting the trials primary outcome relating to hospitalisation. Some patients may require multiple drainage which can be performed if the participant has a re-accumulation of ascites.

Women in the usual care (UC) group will not have the paracentesis performed in an OP setting and will be monitored by their clinical team as per usual care. If usual care at the site involves administering paracentesis on an outpatient basis for treatment of OHSS then the site will be requested to stop routinely providing this for participants in the usual care arm.

Both groups of women will be provided with access to a diary (either in paper format or sent electronically using Research Electronic Data Capture (REDCap)[18] hosted at The University of Sheffield for the purpose of daily self-report of weight, abdominal girth, diarrhoea, vomiting, pain, shortness of breath, fluid input, and urine output. This information will be used by the clinical team in the intervention arm on a daily basis to inform further management and whether to perform further OP. The UC group will also perform daily self-monitoring, but this data will be sent directly to CTRU for input into the database and will not be available to be reviewed by site staff.

Some fertility units undertake other preventative measures (e.g., dopamine agonists, Gonadotropin-releasing hormone (GnRH) antagonists, cessation of IVF cycle) to reduce the risk of OHSS and details of these measures will be collected during the trial, along with information on the provision of care within the conservative management arm. Rarely, patients may be prescribed GnRH antagonists as treatment for established moderate or severe OHSS. This will not be prohibited for trial participants in both arms and data will be collected on GnRH use within the trial.

Participants may wish to stop study treatment, or there may be a clinical need to stop treatment. Participants randomised to the intervention arm, who withdraw from receiving the intervention will continue to be followed-up and will remain in the trial, unless they request to be withdrawn.

For peer review only - http://bmjopen.bmj.com/site/about/guidelines.xhtml

Outcomes

Data collection points are outlined in Table 2.

Primary outcome is any:

- OHSS-related hospitalisation for at least 24 hours within 28 days of randomisation.

Secondary outcomes:

- Need for OHSS related hospitalisation within 28 days – independent blinded central assessment,
- Time to resolution of OHSS assessed within 28 days of randomisation,
- Progression of OHSS severity within 28 days of randomisation,
- Cumulative length of OHSS related hospitalisation,
- Live birth, pregnancy outcomes, neonatal death and serious adverse events (SAEs) including congenital abnormalities in the new-born within 13.5 months of randomisation,
- The occurrence of thrombosis, embolism and significant infections requiring antibiotic treatment or hospitalisation within 90 days of randomisation,
- Adverse events (AEs) within 28 days of randomisation,
- Patient satisfaction assessed using the Client Satisfaction Questionnaire 8 (CSQ-8)[19] based on total scores at 28 days post randomisation,
- EQ-5D-5L participant quality of life daily and at 28 days post randomisation,
- Health resource use and patient costs at 28 days post randomisation.

For peer review only - http://bmjopen.bmj.com/site/about/guidelines.xhtml

Table 2. Data collection time-points

	Baseline/ randomisation (Day 0)	Daily until symptom resolution	Weekly (Days 7, 14, 21, 28)	Symptom Resolution	Symptom Deterioration[#]	During hospitalisation
**Data collected by site staff**						
Demographics and medical history (including medication to prevent OHSS)	OPt					
Symptoms:<br>- Fluid balance including fluid drunk, urine output*<br>- Diarrhoea and vomiting<br>- Nausea**<br>- Abdominal girth<br>- Pain<br>- Shortness of breath<br>- Weight	OPt	SR	T/OP^	OPt	OPt	IP / MN
Blood tests (FBC) – if a face-to-face appointment	OPt		T/OP^	OPt	OPt	IP / MN
Training and provision of consumables, and record details	OPt					
EQ-5D-5L questionnaire	OPt	SR				SR / IP
OHSS related hospital admission within 28 days (primary outcome) and reasons for hospitalisation			T/OPt	OPt	OPt	
Intervention delivery (volume of ascites removed, number of paracentesis)			MN	OPt		MN
Conservative management arm – details of usual care monitoring			MN	OPt		
Use of GnRH antagonist for treatment of OHSS			OPt	OPt	OPt	IP / MN
Confirmation of symptom resolution				OPt		MN
Adverse events (patient)		T***	OPt	OPt	OPt	IP
Pregnancy outcome (e.g miscarriage) and ongoing pregnancies						
Incidence of thrombosis or embolism or significant infection						
Live birth information and pregnancy outcome (e.g. miscarriage)						
Neonatal death, and SAEs related to the baby						
**Data collected by the central study team**						
Health resource use questionnaire						
CSQ-8 questionnaire						
Patient cost questionnaire						
EQ-5D-5L questionnaire						

OPt = Outpatient

IP = Inpatient

If symptoms deteriorate to the point where h

to be required

For peer review only - http://bmjopen.bmj.com/site/about/guidelines.xhtml

T = Data collected over the telephone by a research nurse

SR = Self-reported by the patient

MN = Medical notes

^ If OHSS symptoms have not resolved by this t

Q = data collected via a questionnaire

Follow up

Data will be collected as follows:

- Participant diary data, self-completed daily until symptom resolution.
- Monitoring visits, either face-to-face or remotely, at days 7, 14, 21 and 28 or until symptoms have resolved.
- Symptom resolution visit, face-to-face (unless symptoms resolve during hospitalisation and the data can be collected from medical notes or the participant)
- A symptom deterioration visit, to assess if hospitalisation may be required.
- A participant self-completed questionnaire at 28 days
- Remote follow up at day 90, to collect ongoing pregnancies/pregnancy outcome (e.g., miscarriage), thrombosis/embolism and significant infections. Participants not pregnant at this timepoint will be considered complete and will not require further data collection.
- For participants identified as pregnant at 90 days, live birth and pregnancy outcome (e.g., miscarriage) as well as neonatal death, congenital abnormalities, and serious adverse events for the baby at 13.5 months.

Safety considerations, safety monitoring and adverse event reporting

All AEs and SAEs will be recorded at each participating fertility unit. All AEs/SAEs will be followed up until satisfactory resolution or until the treating clinician and the principal investigator deems the event to be chronic or the participant to be stable. Clinical/research staff will ask participants for any details of AEs at each daily contact in the intervention arm and then in both groups at all timepoints.

Blinding

It will not be possible to blind participants or clinicians to treatment allocation. The trial statistician and TSC will be blinded. To assess the potential impact of outcome assessment

For peer review only - http://bmjopen.bmj.com/site/about/guidelines.xhtml

bias, an additional analysis of the primary endpoint will be performed, using an independent blinded adjudicated primary outcome concerning whether trial participants needed OHSS-related hospitalisation within 28 days.

Trial monitoring and oversight

The trial will be overseen by a TSC and Data Monitoring and Ethics Committee (DMEC). Membership of both will consist of independent experts in the field and the TSC includes a patient representative. The DMEC may advise the chair of the TSC at any time if, in their view, the trial should be stopped for ethical or adaptive reasons.

Day-to-day running of the trial will be coordinated by the Trial Management Group (TMG), consisting of the grant co-applicants, plus members of the Jessop Wing Fertility Unit, Sheffield CTRU, and patient representatives.

Sample size and interim analyses

Under current conservative management, the mean OHSS-related hospitalisation rate is approximately 41.2% (26/63) based on a retrospective audit of medical records of IVF patients across six IVF fertility units over a year [95% confidence interval (CI): 29.0% to 54.4%). Earlier active management interventions based on uncontrolled small previous studies have resulted in low hospitalisation rates of around 0-8% [10,12,13,20]. As such, this trial is targeting a 20% absolute reduction in hospitalisation for OP to be considered superior to usual care. This reduction is believed to be realistic as previous uncontrolled studies have observed similar or greater effects and, if observed, is more convincing to change clinical practice. A 20% absolute reduction translates to an odds ratio (OR) of 0.3825 or relative risk/risk ratio (RR) of 0.512 assuming a 41% usual care event rate. Thus, the targeted effect in terms of OR or RR is a function of the usual care event rate whereas the targeted 20% absolute reduction is consistent across the plausible usual care event rates. An updated preliminary health economics model (trial protocol, via ISRCTN71978064) has indicated that OP needs to achieve at least 4.7% to 5.7% reduction in hospitalisation to become cost-saving under several assumptions about the ratio of early to late OHSS and underlying usual care hospitalisation rates assuming a 20% targeted reduction.

For peer review only - http://bmjopen.bmj.com/site/about/guidelines.xhtml

The aim of the trial is to gather convincing evidence to influence the effects of the early OP that is most likely to change practice regardless of the direction of results and so a group sequential design with an option for early stopping for futility (lack of benefit) is used.

The trial will require a maximum total sample size of ~224 (112 per arm) with an interim analysis when ~146 participants (73 per arm) have accrued primary outcome. This assumes a 90% power, one-sided 2.5% type 1 error, 41% usual care hospitalisation rate, 0% dropout rate, a 20% reduction in hospitalisation rate, and a futility threshold of -4.5% (difference in hospitalisation rate). In this case, the trial will be stopped early for futility if the observed reduction in hospitalisation rate at an interim analysis is less than 4.5%. There is only a 1.9% chance of stopping the trial early for futility in error when OP is truly beneficial. Finally, there is a 72.2% probability of stopping early when the effect of OP is the same as usual care (i.e., 41% hospitalisation rate). The simulation report can be found in Supplementary Material.

Statistical analysis

Detailed of all analysis will be described in an open-access and pre-specified SAP to be developed and signed off before accessing unblinded data.

The primary analysis will be intention-to-treat, including all eligible participants randomised with informed consent. Baseline data will be summarised at the interim and final analyses by treatment group. The absolute difference in hospitalisation rates between arms is the primary summary measure of the treatment effect. RR and OR (estimated using a simple logistic regression model) will also be presented. At an interim analysis, the unadjusted difference in hospitalisation rates between arms will be calculated to inform the interim decision on whether to stop early for futility. For sensitivity analysis, a mixed effects logistic regression model adjusted for stratification factors (site as a random effect and OHSS severity as a fixed effect) and adjusted difference in hospitalisation rates will be obtained using the delta method via margins (30). For the final analysis of the primary outcome, the unadjusted difference in hospitalisation rates with CI obtained via normal approximation will be reported as well as the p-value from a Chi-squared test. Stagewise ordering will be used for sensitivity analysis to obtain the median unbiased estimate of the difference in hospitalisation rates between arms with the associated 95% CI to be presented alongside

For peer review only - http://bmjopen.bmj.com/site/about/guidelines.xhtml

the maximum likelihood estimate if the trial progressed beyond interim analysis. The details of additional sensitivity analyses to be performed adjusting for baseline covariates are found in the SAP. Reporting will adhere to the Adaptive designs CONSORT Extension (ACE) guidance [21].

For progression of OHSS severity, a mixed effects logistic regression model adjusted for stratification factors (site as a random effect and OHSS severity as a fixed effect) will be used and an adjusted difference in hospitalisation rates will be obtained using the delta method via margins (30). Treatment effects will be presented as adjusted odds ratio (aOR), adjusted risk difference (aRD) and adjusted relative risk/risk ratio with 95% CIs.

For time to resolution of OHSS assessed within 28 days post-randomisation, Kaplan-Meier curves will be used to visualise the resolution curves between treatment arms and differences qualified using a log-rank test. Median time to resolution by treatment arm with 95% CI will be calculated and reported as summary measures of within-group effects. Participants who fail to achieve resolution of symptoms within 28 days post-randomisation will be censored. Sensitivity analysis And alternative approaches when the proportional hazard assumption is not met will be detailed in the SAP.

For cumulative length of OHSS hospital stay, bootstrapping resampling procedure (accounting for stratification) will be used to obtain the median difference with 95% CI and associated p-value. For patient satisfaction at 28 days, a total satisfaction score will be analysed using a mixed effects linear regression model adjusted for site (as a random effect) and OHSS severity (as a fixed effect), and the adjusted mean difference between treatment arms with 95% CI and associated p-value will be presented.

Analysis of safety outcomes (e.g., AEs and SAEs) will be based on descriptive statistics using the safety analysis population defined based on treatment-as-received population. Details of additional analysis accounting for repeated events and differential follow-up is found in the SAP.

For peer review only - http://bmjopen.bmj.com/site/about/guidelines.xhtml

Subgroup analyse will be performed to explore whether there is heterogeneity in treatment effect on the primary outcome across the following prespecified subgroups:

1) Baseline severity of OHSS (moderate or severe),

2) Whether a participant is taking a preventative drug at randomisation (yes or no),

3) Whether the participant has early or late OHSS at randomisation,

4) Whether the OP procedure was done vaginally or abdominally. For participants who received multiple procedure (which may happen in few cases), this classification will be based on the first performed procedure.

A mixed effects logistic regression model that includes an interaction effect between pre-planned subgroup and treatment group adjusted for site (as a random effect) and severity of OHSS (as a fixed effect) only if it is not a subgroup factor of interest will be used.

Health Economics

Analyses will be conducted in conjunction with a health economic analysis plan (HEAP). Two HEAPs will be produced, one in relation to the interim analysis, and one relating to the end-of-trial analysis.

The primary cost-effectiveness analysis will present cost per hospitalisation avoided. The feasibility of conducting a cost-utility analysis will be explored in the feasibility study, and if viable, the results will also be expressed as incremental cost per quality-adjusted life years (QALY) gained. An interim health economic analysis will be performed when 65% of the maximum sample size has accrued. The aims of the interim analysis will be to update the pre-trial model and to examine the cost-effectiveness of OP compared to usual care. The QALY will be calculated using the EQ-5D-5L questionnaire [22] administered daily until resolution of symptoms and then at 28 days post randomisation, when women will also be asked to complete resource use questionnaires and a patient cost questionnaire about their last visit to their fertility doctor. Further resource use data will be collected on training of staff and participants on the trial and consumables given; details of monitoring given to participants; outpatient treatment delivery; hospital admission information and treatment received. Unit costs will be derived from appropriate national sources including NHS reference costs and Personal Social Service Research Unit costs [23,24].

For peer review only - http://bmjopen.bmj.com/site/about/guidelines.xhtml

For peer review only

Analyses will be undertaken from the NHS and personal social service perspective as recommended by the National Institute for Health and Care Excellence [25] and will follow recommended methods and good practice guides [26,27]. The primary analysis for both Sub-Protocols within the trial will be a within-trial analysis using data from the trial. A secondary analysis will consider a decision tree model, similar to those used by Casals et al., and Csokmay et al. [16,28], that extends costs and outcomes over a 12 month time horizon. Incremental differences between costs and effectiveness/QALYs between those receiving OP and those receiving usual care will be described and the incremental cost effectiveness ratio will be calculated. Sensitivity analyses and subgroup analyses will also be undertaken.

Qualitative study to aid recruitment

A qualitative study will facilitate the feasibility of conducting the RCT by identifying potential problems with recruitment to the RCT, so that solutions can be instigated rapidly. We will:

1) Identify optimal and sub-optimal practice for recruitment to this trial using audio recording of recruitment sessions

2) Gather information about numbers approached and numbers who consent by assessment of anonymised recruitment logs and other trial documentation

3) Identify problems with the recruitment process by interviewing recruiting healthcare professionals

4) Explore the experiences of women being recruited by interviewing both women who consented, and women who declined, to participate in the trial.

Data Management and monitoring

Data management, confidentiality and access, and monitoring were undertaken in line with Sheffield CTRU SOPs. Details can be found in the full study protocol, accessible via the study webpage: (https://www.sheffield.ac.uk/scharr/research/centres/ctru/stop-ohss).

Data access requests will be reviewed by a sub-committee of the TMG while the trial is on-going and by the Sheffield CTRU in conjunction with project collaborators after the trial has ended.

For peer review only - http://bmjopen.bmj.com/site/about/guidelines.xhtml

Ethics and dissemination

The study is registered on the ISRCTN database (reference: 71978064) and has been approved by the London - Southeast Research Ethics Committee (reference: 22/LO/0015). The findings of this trial will be submitted to peer- reviewed journals and abstracts to national and international conferences. Other stakeholder specific outputs in relevant formats will be produced for trial participants, commissioners, IVF practitioners, third sector and user advocacy organisations.

Patient and public involvement

People with experience of OHSS were involved in the design and development of this project. Patient participation was incorporated in the delivery of the project through representation in the TMG and TSC. The Jessop Wing Reproductive Health Public Advisory Panel contributed to the development of the protocol and study materials.

Acknowledgements

We thank The Jessop Wing Reproductive Health Public Advisory Panel, each local Principal Investigator, Research Nurse/Midwife and research delivery team in the recruitment and management of patients and all members of the central project management team at Sheffield CTRU.

Authors Contributions

Contributors: MM, DW, CP, MD, TY, YC, AD, RM, AO, RC, & AS conceived the study and contributed to the study design. MM, DW, CP, KR, MD, CM, JW, TY, YC, AD, RM, AO, LD & AS initiated the project, developed the protocol, and contributed to implementation. DW, CP, KR, & MM drafted the manuscript. All authors read and approved the final manuscript. MM is the guarantor.

Funding statement

This article presents independent research supported by the NIHR HTA programme [NIHR128137]. The views expressed are those of the author(s) and not necessarily those of the NHS, the NIHR or the Department of Health.

For peer review only - http://bmjopen.bmj.com/site/about/guidelines.xhtml

Competing interests

RM declares a private practice in fertility treatment.

For peer review only

References

1. Boothroyd, C. *et al.* Consensus statement on prevention and detection of ovarian hyperstimulation syndrome. *Aust. New Zeal. J. Obstet. Gynaecol.* **55**, 523–534 (2015).

2. NHS Digital. Hospital Episode Statistics, Admitted Patient Care - England, 2010-11.

For peer review only - http://bmjopen.bmj.com/site/about/guidelines.xhtml

https://digital.nhs.uk/data-and-information/publications/statistical/hospital-admitted-patient-care-activity/hospital-episode-statistics-admitted-patient-care-england-2010-11#resources (2011).

3. Royal College of Obstetricians and Gynaecologists. *RCOG Green-top Guideline No. 5: The Management of Ovarian Hyperstimulation Syndrome*. (2016).

4. Levin, I. *et al.* Effect of paracentesis of ascitic fluids on urinary output and blood indices in patients with severe ovarian hyperstimulation syndrome. *Fertil. Steril.* **77**, 986–988 (2002).

5. Qublan, H. S., Al-Taani, M. I., Megdadi, M. F., Metri, R. M. & Al-Ahmad, N. Multiple transvaginal ascitic fluid aspirations improves the clinical and reproductive outcome in patients undergoing in vitro fertilisation treatment complicated by severe early ovarian hyperstimulation syndrome. *J. Obstet. Gynaecol. (Lahore).* **32**, 379–382 (2012).

6. Chen, C. D. *et al.* Effects of repeated abdominal paracentesis on uterine and intraovarian haemodynamics and pregnancy outcome in severe ovarian hyperstimulation syndrome. *Hum. Reprod.* **13**, 2077–2081 (1998).

7. Fertility treatment 2019: trends and figures | HFEA. https://www.hfea.gov.uk/about-us/publications/research-and-data/fertility-treatment-2019-trends-and-figures/ (2021).

8. Audit Office, N. Reducing emergency admissions A picture of the National Audit Office logo.

9. Smith, L. P., Hacker, M. R. & Alper, M. M. Patients with severe ovarian hyperstimulation syndrome can be managed safely with aggressive outpatient transvaginal paracentesis. *Fertil. Steril.* **92**, 1953–1959 (2009).

10. Lincoln, S. R., Opsahl, M. S., Blauer, K. L., Black, S. H. & Schulman, J. D. Aggressive outpatient treatment of Ovarian Hyperstimulation Syndrome with ascites using transvaginal culdocentesis and intravenous albumin minimizes hospitalization. *J. Assist. Reprod. Genet.* **19**, 159–163 (2002).

For peer review only - http://bmjopen.bmj.com/site/about/guidelines.xhtml

11. Gustofson, R. L., Browne, P., Van Nest, R. L., Richter, K. S. & Larsen, F. W. Aggressive Outpatient Management of Severe Ovarian Hyperstimulation Syndrome Avoids Complications and Prolonged Disease Course. *Fertil. Steril.* **84**, S95 (2005).

12. Fluker, M. R., Copeland, J. E. & Yuzpe, A. A. An ounce of prevention: Outpatient management of the ovarian hyperstimulation syndrome. *Fertil. Steril.* **73**, 821–824 (2000).

13. Shrivastav, P., Nadkarni, P. & Craft, I. Day care management of severe ovarian hyperstimulation syndrome avoids hospitalization and morbidity. *Hum. Reprod.* **9**, 812–814 (1994).

14. Abuzeid, M. I. *et al.* Pigtail catheter for the treatment of ascites associated with ovarian hyperstimulation syndrome. *Hum. Reprod.* **18**, 370–373 (2003).

15. Abuzeid, M. *et al.* Outpatient Management of Severe Ovarian Hyperstimulation Syndrome (OHSS) with Placement of Pigtail Catheter. *Facts, Views Vis. ObGyn* **6**, 31 (2014).

16. Csokmay, J. M. *et al.* Cost analysis model of outpatient management of ovarian hyperstimulation syndrome with paracentesis: 'Tap early and often' versus hospitalization. *Fertil. Steril.* **93**, 167–173 (2010).

17. Gebril, A., Hamoda, H. & Mathur, R. Outpatient management of severe ovarian hyperstimulation syndrome: a systematic review and a review of existing guidelines. *https://doi.org/10.1080/14647273.2017.1331048* **21**, 98–105 (2017).

18. Harris, P. A. *et al.* Research electronic data capture (REDCap)—A metadata-driven methodology and workflow process for providing translational research informatics support. *J. Biomed. Inform.* **42**, 377–381 (2009).

19. Larsen, D. L., Attkisson, C. C., Hargreaves, W. A. & Nguyen, T. D. Assessment of client/patient satisfaction: Development of a general scale. *Eval. Program Plann.* **2**, 197–207 (1979).

20. Lainas, G. T. *et al.* Outpatient management of severe early OHSS by administration of GnRH antagonist in the luteal phase: an observational cohort study. *Reprod. Biol.*

For peer review only - http://bmjopen.bmj.com/site/about/guidelines.xhtml

Endocrinol. **10**, 69 (2012).

21. Dimairo, M. *et al.* The adaptive designs CONSORT extension (ACE) statement: a checklist with explanation and elaboration guideline for reporting randomised trials that use an adaptive design. *Trials 2020 211* **21**, 1–34 (2020).

22. Van Hout, B. *et al.* Interim scoring for the EQ-5D-5L: Mapping the EQ-5D-5L to EQ-5D-3L value sets. *Value Heal.* **15**, 708–715 (2012).

23. Curtis, Lesley A. and Burns, A. Unit costs of health and social care 2019. *Canterbury* (2020) doi:10.22024/UniKent%2F01.02.79286.

24. NHS England » National Cost Collection for the NHS. https://www.england.nhs.uk/national-cost-collection/#archive.

25. Excellence, N. I. for H. and C. *Guide to the methods of technology appraisal 2013*. https://www.nice.org.uk/process/pmg9/resources/guide-to-the-methods-of-technology-appraisal-2013-pdf-2007975843781 (2013).

26. Faria, R., Gomes, M., Epstein, D. & White, I. R. A Guide to Handling Missing Data in Cost-Effectiveness Analysis Conducted Within Randomised Controlled Trials. *Pharmacoeconomics* **32**, 1157–1170 (2014).

27. Manca, A., Hawkins, N. & Sculpher, M. J. Estimating mean QALYs in trial-based cost-effectiveness analysis: the importance of controlling for baseline utility. *Health Econ.* **14**, 487–496 (2005).

28. Casals, G., Fábregues, F., Pavesi, M., Arroyo, V. & Balasch, J. Conservative medical treatment of ovarian hyperstimulation syndrome: a single center series and cost analysis study. *Acta Obstet. Gynecol. Scand.* **92**, 686–691 (2013).

For peer review only - http://bmjopen.bmj.com/site/about/guidelines.xhtml

Figure 1. Study flow chart

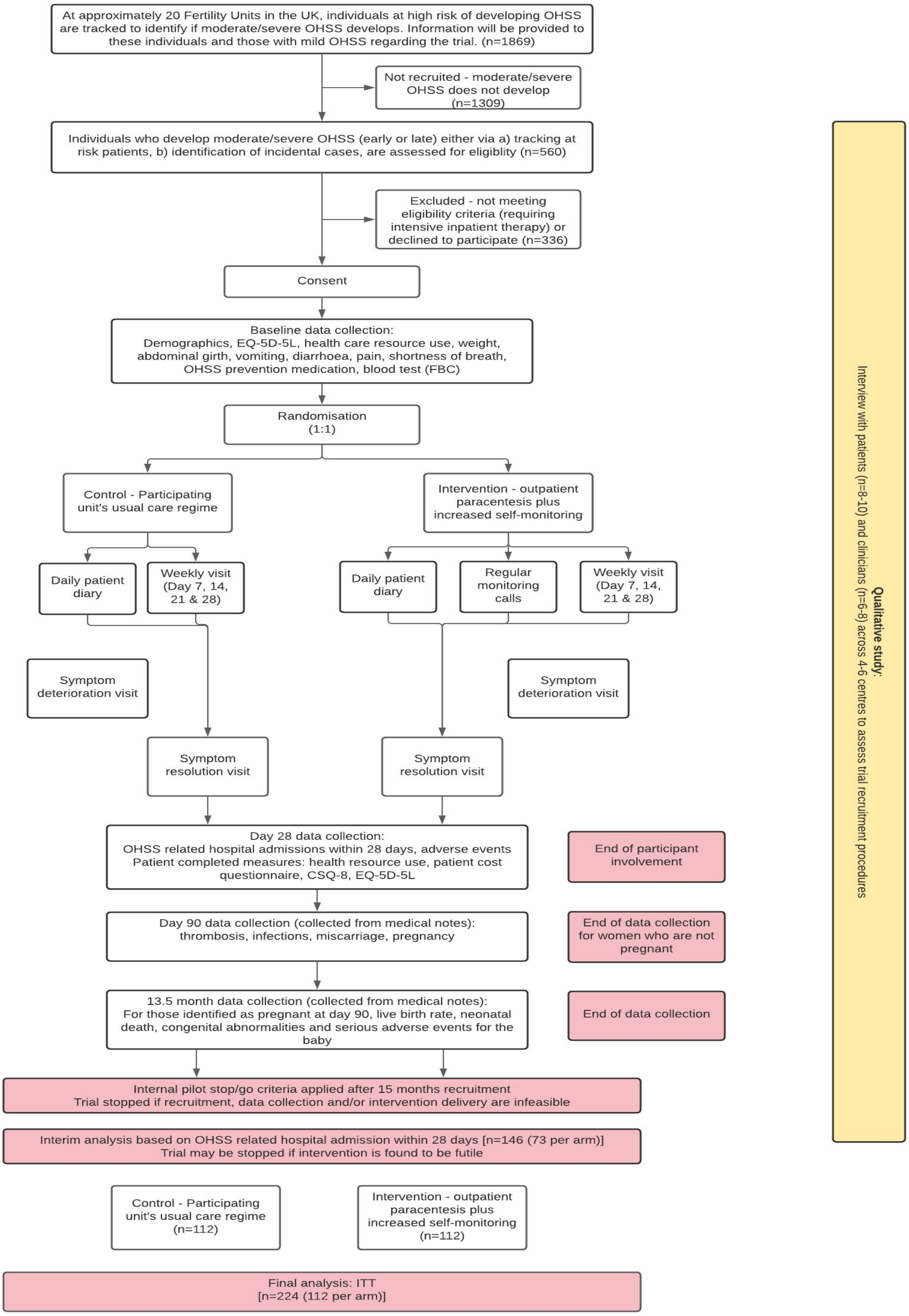

The University Of Sheffield.

Research site NHS logo

STOP OHSS

STOP-OHSS (Shaping and Trialling Outpatient Protocols for Ovarian HyperStimulation Syndrome):

A randomised controlled trial to assess the clinical and cost-effectiveness of active management of Ovarian HyperStimulation Syndrome.

PATIENT INFORMATION SHEET

Protocol Number: STH20532
ISRCTN Number: 71978064
Sponsor: Sheffield Teaching Hospitals NHS Foundation Trust
Clinical Research Office Sheffield
Royal Hallamshire Hospital
D Floor
Glossop Road
Sheffield, S10 2JF
Chief Investigator: Mostafa Metwally

Local Principal Investigator: *[insert name]*

Research Nurse: *[insert name]*
Telephone: *[insert]*
Email: *[insert]*

Introduction

We would like to invite you to take part in our research trial. Before you make a decision, it is important for you to understand why the research is being done and what it will involve. Please take time to read the following information carefully and discuss it with friends and family if you wish to do so. Ask us if there is anything that is not clear or if you would like more information. Take time to decide whether or not you wish to take part. Thank you for reading this.

For peer review only - http://bmjopen.bmj.com/site/about/guidelines.xhtml

The University Of Sheffield.

Research site NHS logo

What is our research about?

Ovarian hyperstimulation syndrome (OHSS) is a condition that can affect women having fertility treatment.

OHSS can be caused by the medication taken to increase egg production during ovulation induction or In Vitro Fertilisation (IVF). The medication known as Follicle-stimulating hormone (FSH) is a hormone which can cause too many eggs to develop, and your ovaries can become large and painful. It can affect women who are still recovering from the effects of this medication given during their fertility treatment and also those who have become pregnant after treatment.

If OHSS occurs up to 7 days after receiving the medication, it is classed as '**Early** OHSS' and if it occurs after 10 or more days, in patients who have become pregnant after treatment, due to their body naturally producing more of the same hormone which can cause the ovaries to become large, is classed as '**Late** OHSS'.

OHSS can range in severity, and can be categorised in the following ways:

- **Mild OHSS** – mild abdominal swelling, discomfort and nausea.
- **Moderate OHSS** – symptoms of mild OHSS, with increased swelling because fluid builds up in the abdomen. This can cause abdominal pain and vomiting.
- **Severe OHSS** – symptoms of moderate OHSS with extreme thirst and dehydration. You may only pass small amounts of urine which is dark in colour and/or you may experience difficulty breathing because of a build-up of fluid in your abdomen. A serious, but rare, complication is a blood clot (thrombosis) forming in the legs or lungs.

Mild symptoms are common and usually resolve quickly, but for some women who experience moderate or severe OHSS these symptoms can worsen. Severe OHSS can lead to serious health complications needing treatment in hospital for several days or weeks, and in a small number of women can be life threatening.

Currently, fertility teams monitor symptoms of OHSS and intervene if the symptoms get worse and the woman needs to be admitted to hospital for intensive monitoring or

For peer review only - http://bmjopen.bmj.com/site/about/guidelines.xhtml

treatment. This treatment can include a procedure call paracentesis and is normally performed as an inpatient.

The aim of this trial is to understand whether taking action earlier by carrying out the same paracentesis procedure but as an **outpatient** and including additional monitoring can stop symptoms getting worse and prevent women having to be admitted to hospital.

About the procedure

Paracentesis is a procedure where a needle is inserted through the vagina or abdomen to drain the fluid that has collected to relieve the OHSS symptoms and is carried out in a similar way to egg collection. It is generally a safe procedure and will not affect your pregnancy if you are already pregnant. Paracentesis is already regularly carried out at many hospitals for patients who are admitted for treatment. Potential complications include feeling tired, dizzy or lightheaded, increased pain in your tummy, low blood pressure or bleeding from the drainage site but these are short term symptoms and will be managed by your clinical team during the procedure. There is also the possibility of infection and injury to major blood vessels or bowel, but these are extremely rare. We will collect data about these side effects, and they will be monitored by the research team. In this study the procedure will be performed by your doctor/nurse/radiologist in an **outpatient** setting to prevent the OHSS symptoms getting worse. It can feel uncomfortable like egg collection, and you will be given pain relief.

What will happen if I take part in the trial?

Women taking part in the trial will be randomly allocated (randomised) by a computer to participate in one of two groups:

Group A) the intervention group will have the procedure called paracentesis (drainage of fluid) which will take place in an **outpatient** setting. They will have extra monitoring from their fertility team which will include filling in a daily patient diary.

Group B) the control group will not have the paracentesis procedure as an outpatient and will be monitored by their fertility team in the usual way, but will be asked to fill in a daily patient diary.

For peer review only - http://bmjopen.bmj.com/site/about/guidelines.xhtml

The University Of Sheffield.

Research site NHS logo

If you decide to take part this means that neither you nor your doctors can decide which treatment you will have and there is an equal chance of being placed in treatment group A or B.

What happens to you if you are randomised to the intervention group?

Your doctor will perform the paracentesis procedure as an **outpatient**. The procedure involves putting a thin needle or tube into your abdomen or vagina, depending on how your hospital usually does this, to remove the fluid which has built up. You may be offered a local anaesthetic for this procedure and we will ask you to stay in clinic for a few hours after the procedure to make sure you feel well enough to go home. Depending on how much fluid is in your abdomen, your doctor will drain the fluid then take out the needle or insert a tube which will be attached to a small drainage bag to collect the fluid from your abdomen. The bag can then be attached to your leg and hidden discreetly under a skirt, dress or loose fitting trousers and, your team will explain how to empty it once you get home. It will be taken out when no more fluid is draining which can take about 2 weeks, but your doctor will discuss which is best for you before carrying out the procedure.

We will ask you to fill in an electronic daily diary recording any OHSS symptoms you have. This will include your weight, how much fluid you have been drinking, how much urine you have passed, whether or not you have any pain or breathlessness and we will also ask you to measure your abdominal girth (the width of your tummy). We will give you a tape measure, weighing scales and measuring jug to do this. A member of the clinical team from your hospital will call you and discuss this diary information with you until you have recovered from the OHSS. This will be daily for the first few days at least but may then be less often. Depending on your symptoms you may be asked to visit the hospital for review by your doctor to give a blood sample or to have more fluid drained by the same **outpatient** paracentesis procedure.

The trial is designed to monitor your symptoms from home and only bring you into the hospital if necessary. For safety reasons we may ask you to visit the hospital weekly, if necessary, during the first 28 days that you are taking part in the trial or if your symptoms start to get worse or have got better, but again this will be discussed with you.

For peer review only - http://bmjopen.bmj.com/site/about/guidelines.xhtml

The University Of Sheffield.

What happens to you if you are randomised to the control group?

Exactly the same will happen as described above but you will not have the paracentesis procedure as an outpatient. However, you may be contacted less frequently by the clinical team, in line with the usual care provided at your centre, this may be every few days, but this will be discussed with you. If your doctor decides based on the information you have given during these calls that you need treatment for your OHSS symptoms, then you will be admitted to hospital as an inpatient. You might still have paracentesis performed but it will be as an inpatient on the ward and not as an outpatient.

Travel expenses for visits to the hospital for trial purposes will be reimbursed (e.g., parking, public transport).

Who is carrying out the research?

This trial is being sponsored (led) by the Sheffield Teaching Hospitals NHS Foundations Trust. *[insert participating site name]* is taking part in the trial as one of the research centres.

Why have I been chosen and what are the possible benefits?

You have been invited to take part in this trial because you have developed moderate or severe OHSS. Taking part in this trial may or may not reduce your chances of going into hospital because of OHSS, but information from this trial may help doctors understand whether it would be helpful to offer this **outpatient** procedure to women who have moderate or severe OHSS in the future.

We plan to recruit 224 women who are suffering from moderate or severe OHSS over 3 years. It will take place in about 20 fertility units in the UK. Half of these women will receive the paracentesis procedure as an **outpatient** and have increased monitoring, and half will not.

Do I have to take part?

No. It is up to you to decide whether or not to take part. If you do decide to take part you will be given this information sheet to keep and will be asked to sign a consent form. If you decide to take part you can still decide to withdraw at any time and without giving a reason.

If you decide to withdraw at any time, or not to take part, this will not affect the standard of care you receive. If you withdraw from the trial, any anonymised information we have

For peer review only - http://bmjopen.bmj.com/site/about/guidelines.xhtml

The University Of Sheffield.

Research site NHS logo

already collected about you will be included in the final data analysis, but we will not collect further information without your consent.

Providing information and data as part of the trial

All women, regardless of which group you are in, will be followed up to see how long it takes for your symptoms to get better and whether hospital admission is needed. We will also compare the safety, acceptability and cost of each treatment. We will ask you to complete questionnaires about your health, how you felt about the service you received and any health care you needed 28 days after you gave consent to take part in the trial. These can be completed by post or online. We will also ask you to complete a daily diary as described in the above section "**What happens to you if you are randomised to the intervention group?**".

You will be contacted by telephone by a research nurse/midwife or DR, or asked to come into hospital for the following reasons:

- A weekly visit (up to 28 days or until your symptoms have got better)
- If your symptoms start to get worse
- Once your symptoms have resolved

During these visits, the doctor/ nurse will ask you some questions about your symptoms. Information will also be collected from your medical notes about your symptoms and details of any time spent in hospital because of OHSS.

If you are taken into hospital during the trial at any other NHS Trust, this information may also be requested from them. We will ask your permission to do this.

We would also like to review your medical notes around 3 months (90 days) after you agree to take part in the trial to collect any information about any infections or blood clots (thrombosis) that you may have experienced. At this point we will also collect any information about whether you are pregnant or any pregnancy outcome. If you are not pregnant we will not collect any further data from your notes. If, however, we find that you are pregnant we will review your notes again around 10 months later (13.5 months after you first joined the trial) to collect information about the outcome of your pregnancy.

For peer review only - http://bmjopen.bmj.com/site/about/guidelines.xhtml

The research team will compare how many people were admitted to hospital from both groups at the end of the trial. This will help us to work out whether early paracentesis as an **outpatient** reduces the chance of women suffering from moderate or severe OHSS being taken into hospital.

For peer review only - http://bmjopen.bmj.com/site/about/guidelines.xhtml
For peer review only

The University Of Sheffield.

Research site NHS logo

Summary of what will happen if you take part in this trial

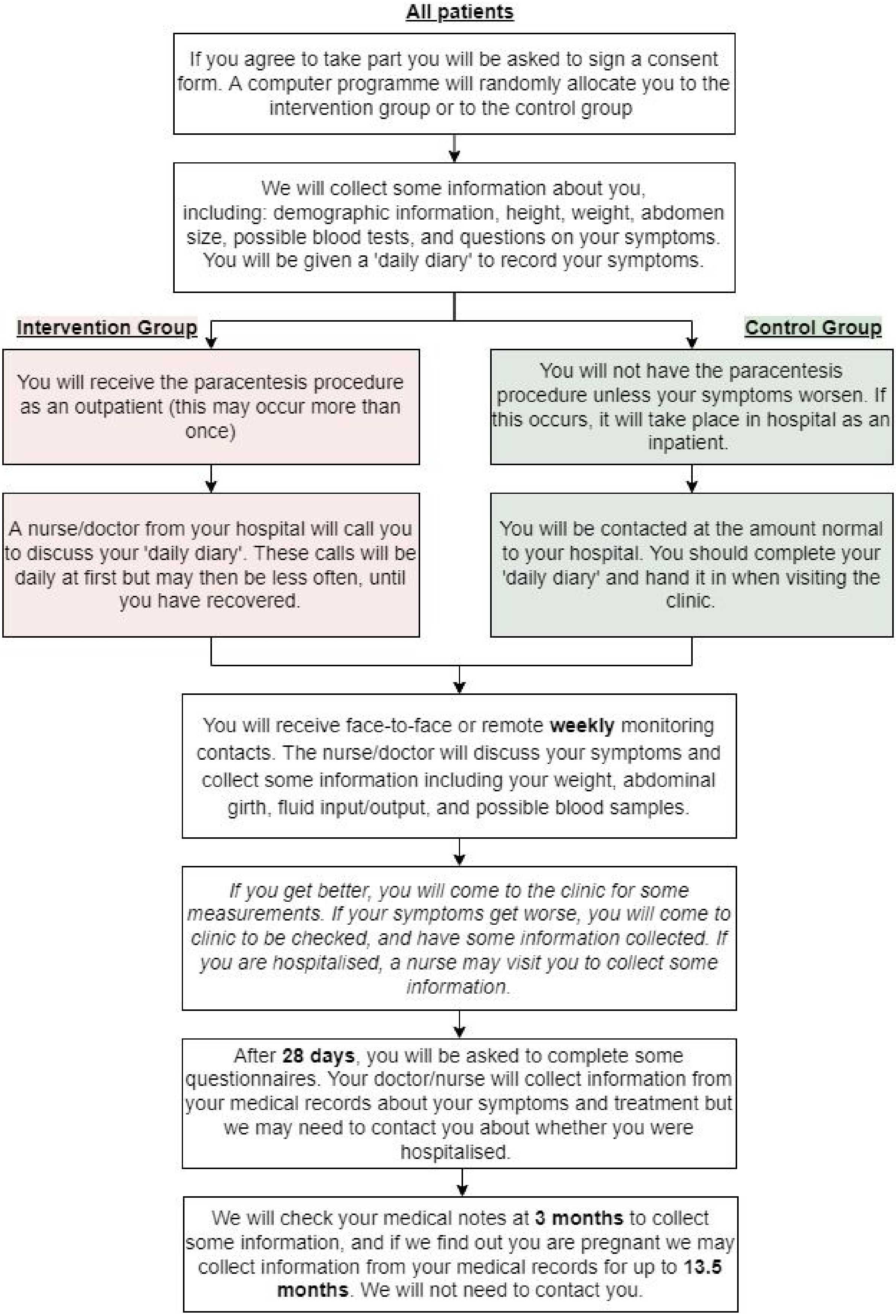

For peer review only - http://bmjopen.bmj.com/site/about/guidelines.xhtml

What are the possible disadvantages and risks of taking part?

Paracentesis is a relatively safe procedure, but there are some risks, which have been described in the section '**About the procedure'**, and these should be discussed with your doctor/ nurse.

Additionally, completing the patient diary daily for 28 days may be an inconvenience to you. This includes measurements of weight, abdominal girth, fluid intake and urine output. For patients in the intervention group, we will use this information to monitor your condition as best as possible. The data that both groups provide in these diaries will help us to answer our research question. We will explain how you should collect the data and provide you with any equipment needed.

How will we use information about you?

Sheffield Teaching Hospitals NHS Foundation Trust (STH NHSFT) is the sponsor that is leading this trial and will act as the data controller. This means that they are responsible for looking after your information and using it properly. The trial is managed by the Clinical Trials Research Unit (CTRU) in the School of Health and Related Research at The University of Sheffield.

Together STH NHSFT and the CTRU will need to use information from you and your medical records for this trial. This information will include your consent to take part in the trial, initials, NHS number (or CHI number if in Scotland), name, contact details and date of birth. STH NHSFT and the CTRU will use this information to do the research or to check your records to make sure that the research is being done properly. Members of the research team who do not need to know who you are will not be able to see your name or contact details. Your data will have a code number instead.

We will keep all information about you safe and secure.

STH NHSFT, the CTRU and *[insert site name]* will keep your data securely for 15 years after the end of the trial.

No one would be able to work out that you took part in the trial from the reports we write about it.

For peer review only - http://bmjopen.bmj.com/site/about/guidelines.xhtml

The University Of Sheffield.

Research site NHS logo

What are your choices about how your information is used?

You can stop being part of the trial at any time, without giving a reason, but we will keep information about you that we already have, as it may affect our results.

- If you choose to stop taking part in the trial, we would like to continue collecting information about your health from your hospital records. If you do not want this to happen, please let us know and we will stop.
- We need to manage the information that we collect in specific ways for the research to be reliable. This means that we won't be able to let you see or change the data we hold about you.
- If you agree to take part in this trial, you may have the option to take part in future research using your data saved from this trial.
- The anonymised data may be used to support other research in the future, and may be shared with other researchers for comparison studies

Where can you find out more about how your information is used?

You can find out more about how we use your information here www.sheffieldclinicalresearch.org/for-patients-public/how-is-yourinformation-handled-in-research/

What will happen to the results of the research trial?

The results of this trial will be published in scientific journals and presented at scientific meetings, as well as shared directly with NHS Trusts, professional bodies and patient groups. You will not be identified in any report or publication. If you would like to receive a summary of the trial results and a copy of the research report, we will send this to you.

Who is sponsoring and funding the research?

This trial is funded by the National Institute for Health Research (NIHR) Health Technology Assessment (HTA) and sponsored by Sheffield Teaching Hospitals NHS Foundation Trust.

It is organised, managed and coordinated by the University of Sheffield, and data will be collected and stored by this institution.

For peer review only - http://bmjopen.bmj.com/site/about/guidelines.xhtml

The University Of Sheffield.

Additionally, the trial will be supervised on a regular basis by a Data Monitoring and Ethics Committee (DMEC) and a Trial Steering Committee (TSC). The primary function of the DMEC is to ensure the absolute safety of all participants.

Who has reviewed the trial?

Research in the NHS is looked at by an independent group of people called a Research Ethics Committee to protect your interests. This trial has been reviewed and given a favourable opinion by the London - South East Research Ethics Committee.

The trial has also been reviewed by the Reproductive Health Research Public Advisory Panel at the Jessop Wing, Sheffield. This is a group of lay members whose main purpose is to ensure that research carried out is patient focused. The role of the panel is to give a patients' perspective on the research that is being carried out.

What if I wish to complain about the way in which this trial has been conducted?

If you have a concern about any aspect of this trial you should ask to speak with the researchers who will do their best to answer your questions. If you remain unhappy and wish to complain formally, you can do this through the NHS complaints procedure, by contacting the Patient Liaison Service (PALs).

Patient Advice and Liaison Service:

Telephone: 0151 702 4353

Email: PALS@lwh.nhs.uk

Harm

Your participation in this trial is to help us find out if performing this procedure in women suffering from moderate or severe OHSS reduces the chance of being admitted to hospital. In the event that something does go wrong, and you are harmed during the research, and this is due to someone's negligence then you may have grounds for a legal action for compensation, but you may have to pay your legal costs. The normal National Health Service complaints procedure will still be available to you.

Further information and contact details for local research team

You are free to ask questions about this information sheet or the trial now or at any time

For peer review only - http://bmjopen.bmj.com/site/about/guidelines.xhtml

The University Of Sheffield.

Research site NHS logo

during the trial. If you experience any side effects, or if you have any questions about this research during this trial you may contact: kirsty.mckendrick@sheffield.ac.uk

The trial website contains information that may be helpful. The website is located at www.sheffield.ac.uk/scharr/research/centres/ctru/stop-ohss. If you agree to participate in this trial, you will receive a signed and dated copy of the consent form and this patient information sheet for your records. Whether or not you wish to participate in our trial, we would like to thank you for taking the time to read this information sheet.

For peer review only

For peer review only - http://bmjopen.bmj.com/site/about/guidelines.xhtml

Research site NHS logo

STOP-OHSS (Shaping and Trialling <u>Outpatient</u> Protocols for Ovarian HyperStimulation Syndrome):

A randomized controlled trial to assess the clinical and cost-effectiveness of active management of Ovarian HyperStimulation Syndrome.

<u>CONSENT FORM</u>

Local Principal Investigator: *[insert name]*
Centre Number/ID:
Participant Identification Number:

Ple[illegible]itial

1. I can confirm that I have read and understood the participant information sheet version 3.0 dated 12Dec22 for the STOP-OHSS trial and have had the opportunity to ask questions and these have been answered satisfactorily. ☐ (Your initials)

2. I understand that my participation in the trial is voluntary and I am free to withdraw at any time without my treatment or legal rights being affected. ☐ (Your initials)

3. I understand that my local research team and the study organisers at the University of Sheffield (Clinical Trials Research Unit) will each have access to personal information about my progress and may receive a copy of my consent form for monitoring purposes. Transfer of this information between organisations will be done in confidence via secure email or a secure access controlled database. ☐ (Your initials)

4. I understand that the information collected will be used for medical research only and that I will not be identified in any way in the analysis and reporting of the results. ☐ (Your initials)

5. I understand that even if I withdraw from the trial, information already collected about me may be included in the final analysis after being anonymised. ☐ (Your initials)

For peer review only - http://bmjopen.bmj.com/site/about/guidelines.xhtml

The University Of Sheffield.

Research site NHS logo

6. I understand that relevant sections of my medical notes and data collected during the trial may be looked at by members of the research team (at Sheffield Teaching Hospitals NHSFT or the Clinical Trials Research Unit at the University of Sheffield), or my participating trial centre, or by individuals from statutory regulators. I give permission for these individuals to have access to my records. ☐ (Your initials)

When completed: 1 for participant; 1 for researcher site file; 1 to be kept in medical notes

7. I understand that if I am hospitalised during my time in the trial at an NHS Trust or a private centre other than Sheffield Teaching Hospitals NHSFT then the research team will seek to obtain data about the hospitalisation from the other organisation for the purpose of this study. I give my permission for this. ☐ (Your initials)

8. I understand that information held by the NHS may be used to keep in touch with me to follow up on my health and pregnancy status for the purpose of the trial. ☐ (Your initials)

9. I understand that if I become pregnant during the course of the trial that information about the outcome of my pregnancy including relevant sections of my child's medical notes (if applicable) may be looked at by members of the research team (at Sheffield Teaching Hospitals NHSFT or the Clinical Trials Research Unit at the University of Sheffield) or Sheffield Teaching Hospitals participating trial centre, or by individuals from statutory regulators. I give permission for these individuals to have access to my child's records. ☐ (Your initials)

10. I understand that by agreeing to take part in this study I also agree to completing trial visits and follow-up questionnaires. The Sheffield CTRU will be given my contact details in order to send these. ☐ (Your initials)

11. I understand that data collected about me without personal identifiers may be used to support other research in the future, and may be shared with other researchers at other institutions to enable them to undertake studies for which my data may be useful; and I give permission for this. ☐ (Your initials)

12. I understand the information that I have been given about the above study and I agree to take part. ☐ (Your initials)

The following are optional and additional, answering "No" to any will not affect your ability to take part in the study.

When completed: 1 for participant; 1 for researcher site file; 1 to be kept in medical notes

For peer review only - http://bmjopen.bmj.com/site/about/guidelines.xhtml

The University Of Sheffield.

Research site NHS logo

	**Yes**		**No**
13. I understand that if I choose to stop taking part in the study, the research team would like to continue collecting information about my health from my medical notes, and if applicable those of my child, and I give permission for them to do this.	☐ (Your initials)	OR	☐
14. I understand I may be contacted in the future to give my consent to take part in future studies similar to this project.	☐ (Your initials)	OR	☐ No
15. I am happy for a member of the research team to contact me about taking part in an interview about my experience in this trial.	☐ (Your initials)	OR	☐ No

____________________ ___________ ________________

Name of participant: Date: Signature:

____________________ ___________ ________________

Name of person taking consent: Date: Signature:

When completed: 1 for participant; 1 for researcher site file; 1 to be kept in medical notes

For peer review only - http://bmjopen.bmj.com/site/about/guidelines.xhtml

For peer review only

STOP-OHSS Simulation Report Final Version 2.0

STOP-OHSS Trial Simulation Report: Summary report on sample size calculation and operating characteristics of the group sequential trial

Munya Dimairo [a], Laura Sutton [b], Tracey Young [c], Ellen Lee [a], Cara Mooney [a], and David White [a]

[a] *Clinical Trial Research Unit (CTRU); Design, Trials and Statistics (DTS), School of Health and Related Research (ScHARR); University of Sheffield*

[b] *DTS, ScHARR, University of Sheffield*

[c] *Health Economics and Decision Science (HEDS), ScHARR, University of Sheffield*

The contribution of the authors is described in Section 10.

For peer review only

For peer review only - http://bmjopen.bmj.com/site/about/guidelines.xhtml

STOP-OHSS Simulation Report Final Version 2.0

Contents

1 Background 3
2 Findings from a retrospective audit study 3
2.1 Characterisation of the eligible patient population 3
2.2 Estimates of hospitalisation rate in the eligible population 3
2.3 Summary 4
3 Design, trial adaptations and interim decision-making criteria 4
3.1 The design 4
3.2 Trial adaptations and interim analysis aspects 4
3.3 Interim decision-making criteria 5
4 Sample size calculation 5
5 Statistical simulations 6
5.1 Objectives 6
5.2 Approach to simulations 6
6 Results 6
6.1 Summary of sample sizes 6
6.2 Impact of futility thresholds and uncertainty in usual care event rate 7
6.2.1 Impact on the probability of declaring efficacy correctly or in error. 8
6.2.2 Impact on the probability of stopping early for futility correctly or in error. 9
7 Summary 11
8 Limitations....11
9 Impact of early stopping on study duration and sample size saving....12
10 Contributions 12
11 Acknowledgements....12
12 References 12

For peer review only - http://bmjopen.bmj.com/site/about/guidelines.xhtml

STOP-OHSS Simulation Report Final Version 2.0

1 Background

This report summarises findings from a retrospective review of medical records of patients undergoing in-vitro fertilisation (IVF) treatments that inform the redesign of the STOP-OHSS trial. In addition, the design features of the STOP-OHSS trial, sample size estimates, and the statistical behaviour of the design in addressing the research questions under different assumptions are presented. Finally, the limitations of the design and implications of early stopping on trial duration are also discussed.

2 Findings from a retrospective audit study

This study was undertaken to get a better understanding of the pool of potentially eligible patients with early and late ovarian hyperstimulation syndrome (OHSS) to assess the feasibility of the initially proposed design and recruitment. Importantly, this was also to estimate the OHSS-related hospitalisation rate in the usual care to validate the assumptions made during the design of the original design or use this to modify the design accordingly. Finally, we wanted to understand how the usual care hospitalisation rate differs by proposed study populations (early versus late OHSS) and severity of OHSS (moderate versus severe).

2.1 Characterisation of the eligible patient population

Across five IVF sites, a total of 134 cycles from 134 patients were returned from searches performed over a year (1st March 2019 to 29th February 2020), of which, 63 would have been eligible to be recruited into this trial Of these 63 eligible patients, 25 (39.7%) had early OHSS and 38 (60.3%) had late OHSS. In addition, most patients 73.0% (46/63) were moderate OHSS cases (95% confidence interval, CI: 60.3% to 83.4%) and only 27.0% (17/63) were severe OHSS cases (95% CI: 16.6% to 39.7%).

2.2 Estimates of hospitalisation rate in the eligible population

Of the 63 eligible patients, 26 were hospitalised relating to OHSS, which translated to a 41.2% mean hospitalisation rate across five sites (95% CI: 29.0% to 54.4%).

Only 11 of the 25 eligible patients with early OHSS were hospitalised, representing a mean hospitalisation rate of 44.0% (95% CI: 24.4% to 65.1%). In the late OHSS population, 15 of the 38 eligible patients were hospitalised, translating to a mean hospitalisation rate of 39.5% (95 %CI: 24.0% to 56.6%).

Of the 46 eligible patients with moderate OHSS, 16 (34.8%) were hospitalised (95% CI: 21.4% to 50.2%). In 17 eligible patients with severe OHSS, there was a 58.8% (10/17) hospitalisation rate (95% CI: 32.9% to 81.6%).

For peer review only - http://bmjopen.bmj.com/site/about/guidelines.xhtml

2.3 Summary

These estimates are based on OHSS patients from only 5 sites of the expected 20 sites so results should be interpreted with existing uncertainties in mind. However, the estimates give us some insights into eligible patients in the early and late OHSS populations, hospitalisation rates and how this varies by subpopulation and severity of OHSS. Specifically, cases of late OHSS appear to be higher than early OHSS, which is the opposite of what was expected. This strongly suggests that it is unlikely to be feasible to conduct a three-arm definitive trial in the early OHSS population as originally designed to investigate the effectiveness of early outpatient management interventions using paracentesis or a gonadotrophin-releasing hormone (GnRH) antagonist drug compared to usual care. Similarly, the cases of late OHSS seem to be much higher than expected so there is scope for a trial that is more than just exploratory as originally envisaged.

As a result, the Trial Management Group agreed to make changes to the original design by dropping one of the proposed study treatments in the early OHSS population (GnRH antagonist drug) and combining the early and late OHSS populations. Response to treatment in the early and late OHSS population is expected to be similar although the underlying hospitalisation rate may differ as shown in Section 2.2.

3 Design, trial adaptations and interim decision-making criteria

3.1 The design

In an open-label, pragmatic, multi-centre, superiority, parallel-group, group sequential, randomised controlled trial; participants with either early or late OHSS will be individually randomised (1:1) to receive early outpatient paracentesis (OP) or usual care (which excludes early OP). The primary outcome is any OHSS-related hospitalisation for at least 24 hours within 28 days from randomisation.

3.2 Trial adaptations and interim analysis aspects

The goal of the trial is to gather convincing evidence about the effects of the early OP that is most likely to change practice regardless of the direction of results. The recruitment to this trial is expected to be quite challenging so there is a need to pursue the trial only when the study treatment is viewed as promising to use research resources, study patients, and time efficiently. This motivated the use of a group sequential design with an option for early stopping for futility (lack of benefit). The decision to stop for futility is non-binding in the sense that it is advisory so it can be ignored or overruled for some reasons without affecting the statistical behaviour of the design (i.e., it does not increase the type I error beyond the budgeted 2.5% for a one-sided test). One interim analysis will be performed when 65% of the maximum sample size had accrued primary outcome data on hospitalisation within 28 days of randomisation. This delayed timing of interim analysis has been chosen to allow sufficient data to be gathered on hospitalisation rates in both arms to inform reliable interim decisions.

For peer review only - http://bmjopen.bmj.com/site/about/guidelines.xhtml

STOP-OHSS Simulation Report Final Version 2.0

Of note, the trial does not allow for early stopping for overwhelming benefit (efficacy). In addition, the continuation of the trial after interim analysis does not necessarily imply that the study treatment is effective but it only indicates that early OP is promising and worth pursuing further to assess if it is truly effective at the end.

3.3 Interim decision-making criteria

Decision rules at an interim analysis should be fit for purpose and decisions made should be reliable, sensible, and yield convincing and interpretable results. If early OP is found to be effective, the goal is to reduce the hospitalisation rate thus reducing the burden on the NHS, patients, and society, which is cost-saving. To claim the efficacy of early OP, the research team has targeted a realistic and worthwhile 20% reduction in hospitalisation rate that is viewed to be important to influence practice. To inform early stopping, the minimum reduction in hospitalisation below which early OP is viewed as futile needs to be predefined. In addition, a good stopping rule should balance the need to reduce the risk of stopping early for futility of a treatment that is potentially cost-saving and/or effective with the desire to increase the chances of early stopping when treatment is unlikely to be cost-saving and clinically effective.

To help achieve this, a preliminary health economic model which was developed at the grant development stage was updated based on estimated costs associated with early OP and usual care and results from a retrospective audit study (Section 2). This was done under several assumptions about the ratio of early to late OHSS and underlying hospitalisation rates assuming a 20% targeted reduction (if early OP is effective). **Results showed that early OP needs to achieve at least between 4.7% and 5.7% reduction in hospitalisation to become cost-saving** (see appendix found in protocol https://www.isrctn.com/ISRCTN71978064). As such, a sensible futility stopping rule needs to use a value less than 4.7%, which is only 23.5% of the targeted effect of 20% reduction. To further guide the choice of the futility threshold, we explored the statistical behaviour of the design through simulations using futility thresholds (hospital reduction) of 0, -2.5%, -3.0%, -3.5%, -4.0%, and -4.5% (see Section 6.2).

4 Sample size calculation

Sample sizes were estimated using the *rpact* [1] R package without continuity correction under the following assumptions:

- 1:1 allocation ratio, 90% power and 2.5% one-sided type I error rate;
- the best estimate of the usual care hospitalisation rate of 41% (Section 2.2);
- 20% targeted difference in hospitalisation rates between treatment arms;
- one interim analysis at 65% of the maximum total sample size;
- 0% dropout rate;
- futility stopping thresholds of 0, -2.5%, -3.0%, -3.5%, -4.0%, and -4.5%.

For peer review only - http://bmjopen.bmj.com/site/about/guidelines.xhtml

STOP-OHSS Simulation Report Final Version 2.0

5 Statistical simulations

5.1 Objectives

Simulations were undertaken to:

1) explore the impact of possible futility thresholds such as on the probability of stopping the trial under a specific assumption about the known treatment effect and the decision-making errors (e.g. incorrectly stopping for futility when the early OP is actually effective);
2) help the research team select an appropriate stopping threshold to use;
3) explore the impact of existing uncertainty in the usual care hospitalisation rate (which can vary from 29% to 54%) on study power.

5.2 Approach to simulations

Simulations were performed as follows in that order:

1. use the calculated 224 maximum total sample size (112 per arm) corresponding to a futility threshold of -4.5% (reduction in hospitalisation);
2. generate pseudo-trial data with the number of patients calculated as in item 1 using a binomial distribution assuming a certain treatment effect (differences in proportions of 0%, 5%, 10%, 15%, and 20% were used) and underlying usual care event rate (hospitalisation rate of 29% to 50% with an increment of 3% were used);
3. for each scenario, repeat the process to generate 100 000 trials sufficient to produce accurate results;
4. for each simulated pseudo-trial, analyse interim data from the first 65% of participants and decide whether the trial should have been stopped at this point using a selected futility threshold. This was repeated for futility thresholds of 0, -2.5%, -3.0%, -3.5%, -4.0%, and -4.5% (hospitalisation reduction);
5. for each simulated pseudo-trial, what the overall decision about clinical efficacy would have been if it was not stopped early was noted;
6. using data from all 100 000 simulated pseudo-trials, the proportions of trials that would have been stopped for futility and those that indicated efficacy if they were not stopped early were calculated.

All simulations were performed in Stata v16.1 using a bespoke program and results are presented in Section 6.2.

6 Results

6.1 Summary of sample sizes

Table 1 summarises the total maximum sample sizes and total sample sizes required for interim analysis under different assumptions. For example (row marked in green), the trial

For peer review only - http://bmjopen.bmj.com/site/about/guidelines.xhtml

STOP OHSS

STOP-OHSS Simulation Report Final Version 2.0

will require a maximum total sample size of ~224 (112 per arm), rounded upwards to the nearest even number. An interim analysis will be performed when ~146 participants (73 per arm) had accrued primary outcome data on hospitalisation within 28 days of randomisation. This assumes a 90% power, one-sided 2.5% type 1 error rate, 41% hospitalisation rate in the usual care arm (see Section 2.2), a 20% worthwhile and realistic reduction in hospitalisation rate (as per original design), and a futility threshold of -4.5% (difference in hospitalisation rate). In this case, a trial will be stopped early for futility if the observed reduction in hospitalisation rate at an interim analysis is less than 4.5% (equivalent to a one-sided p-value above 2.9 or critical value below 0.553). There is only a **1.9% chance** of stopping the trial early for futility in error for a beneficial treatment which is very low as expected.

As noted in Table 1, the total sample size is very similar across futility thresholds (0% to 4.5%). However, the influence of the size of the futility threshold on the chances of stopping early is apparent in Figure 2.

Table 1. Estimated total sample sizes assuming 90% power and 2.5% one-sided type I error.

Usual care event rate	A targeted difference in hospitalisation rates	Futility stopping boundaries at interim analysis			Efficacy boundaries at:		Sample sizes across groups		Probability of early stopping when paracentesis is efficacious
		Critical value	Difference in proportions (paracentesis – usual care)	One-sided p-value	An interim analysis	The final analysis	Interim analysis (65% of the total)	In total	
41%	-20%	0	0	0.50	N/A	-0.126	144	221	0.0045
41%	-20%	0.305	-0.025	0.38	N/A	-0.125	144	222	0.0104
41%	-20%	0.366	-0.030	0.357	N/A	-0.125	145	222	0.0121
41%	-20%	0.429	-0.035	0.334	N/A	-0.125	145	222	0.0142
41%	-20%	0.491	-0.040	0.312	N/A	-0.125	145	223	0.0165
41%	-20%	0.553	-0.045	0.290	N/A	-0.125	145	223	0.0191

Assumptions: 1:1 randomisation; 0% dropout rate; interim analysis to be performed when 65% of the maximum total sample size had accrued outcome data; NA, not applicable.

6.2 Impact of futility thresholds and uncertainty in usual care event rate

A good trial design should facilitate reliable decisions with high probability both at interim analysis and at the end if it progressed beyond interim analysis. For example, if we simulate many pseudo trials assuming that a treatment is effective with a targeted treatment effect we wish to see (e.g., 20%), a very high proportion of these trials should progress after interim analysis and the treatment should be deemed efficacious in the end. On the contrary, if we simulate assuming that a treatment does not work (0% effect say), we should expect most trials to indicate early stopping and only a negligible fraction to indicate efficacy in the end (i.e., making erroneous conclusion claiming efficacy, type I error).

For peer review only - http://bmjopen.bmj.com/site/about/guidelines.xhtml

6.2.1 Impact on the probability of declaring efficacy correctly or in error.

As evident in Figure 1, across all presented scenarios, the choice of futility threshold between -4.5% and 0 has a negligible effect on the chances of declaring efficacy either correctly or incorrectly.

Figure 1 (**A**) shows the power of the study as the usual care hospitalisation rate changes from 29% to 50%. If the underlying hospital reduction is as assumed (20%), the study will still have more than 87% power even if the usual care hospitalisation rate is as high as 50%. That is, regardless of the usual care hospitalisation rate, the study will have sufficient power (above or close to the assumed 90%) to find a 20% hospital reduction if the treatment is truly effective with this effect.

On the contrary, in Figure 1 (**B**), if the observed hospital reduction is 15% (rather than the assumed 20%), the power of the study will range from 62% to 79% to find a 15% hospital reduction if early OP is effective with this effect as the usual care hospitalisation rate decreases from 50% to 29%, respectively. Figure 1 (**C**) shows that the study will be severely underpowered to find a 10% hospital reduction across all scenarios of the usual care hospitalisation rate even though this effect is likely to be cost-saving based on the updated preliminary health economic model.

Figure 1 (**E**) shows the chances of erroneously claiming that early OP is effective when its hospitalisation rate is the same as in the usual care (i.e., 0% hospital reduction). This error is very small as expected and ranges from 2.4% to 2.6% across (accounting for Monte Carlo error) all scenarios compared to the targeted 2.5% for a one-sided test.

In summary, if the underlying treatment effect is at least 20% hospital reduction, the study will be sufficiently powered across all realistic scenarios of the usual care hospitalisation rate and this is not affected by the choice of the futility threshold of between -4.5% and 0%. In addition, the chance of incorrectly declaring efficacy when treatment is ineffective is very small and as expected. However, the study will be severely underpowered for effects that are far less than 20% but likely to be cost-saving (e.g., 5% to 15%).

For peer review only

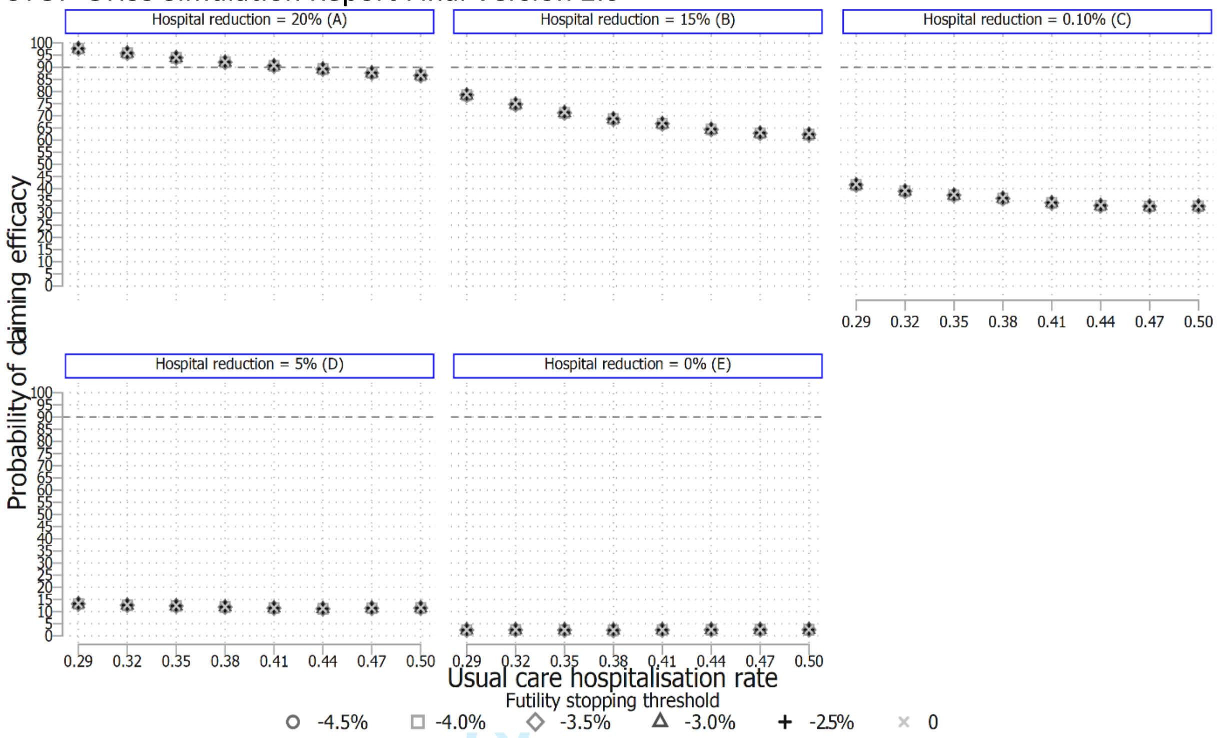

Figure 1. Impact on the chances of claiming efficacy at the end of the trial.

6.2.2 Impact on the probability of stopping early for futility correctly or in error.

Figure 2 presents the chances of stopping early for futility under different assumptions of the underlying treatment effect, usual care hospitalisation rate, and futility stopping threshold. As evident, the impact of futility stopping threshold becomes more apparent as the underlying hospital reduction changes from 20% to 0%. In principle, we want a design that:

1) allows early stopping with high probability when the underlying treatment effect is in support of the null hypothesis of no difference and,
2) erroneously leads to trial early stopping with very low or negligible probability when it is known that the treatment is beneficial.

In Figure 2 **(A)**, if early OP is effective, the chances of stopping early range from 0% to 2.9% as the futility stopping threshold decreases from 0 to -4.5% and the usual care hospitalisation rate increases from 29% to 50%, respectively. For example, if the underlying treatment effect is 20% as hoped and the usual care event rate is 41% (current best estimate), there is only approximately a 2.2% chance of stopping early for futility if a stopping threshold of -4.5% is used.

Figure 2 **(E)** is a scenario when it is known that the treatment effect is **0%** and a good design should trigger early stopping with high probability. As evident, this is achieved across all scenarios when a futility stopping threshold of -4.5% is chosen. For example, this probability is 72.2% when the usual care hospital rate is 41% (current best estimate).

For peer review only - http://bmjopen.bmj.com/site/about/guidelines.xhtml

STOP OHSS

STOP-OHSS Simulation Report Final Version 2.0

Figure 2 (subfigures **B-D**) are interpreted in the same manner when the underlying treatment effect is 15%, 10%, and 5% respectively.

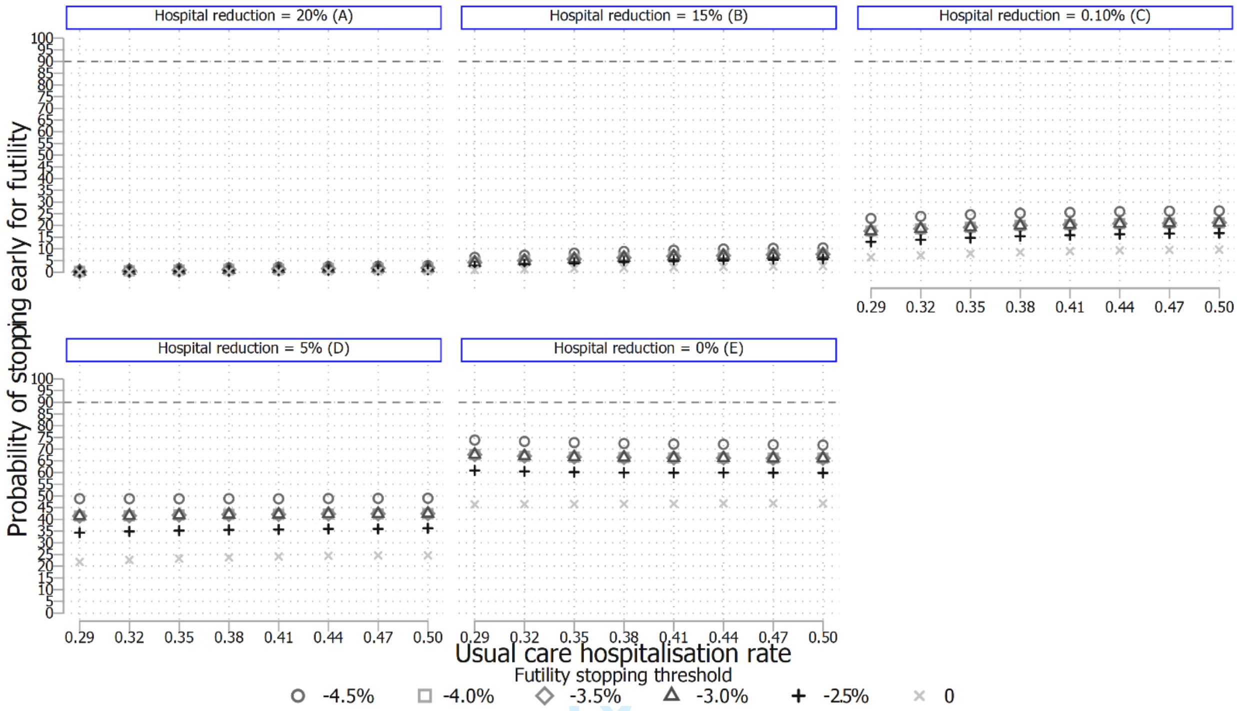

Figure 2. Impact on the chances of stopping early correctly or in error.

In summary, when choosing a futility stopping threshold, there is a trade-off between increasing the chances of early stopping for an ineffective treatment (scenario **E**) and reducing the risks of stopping a trial early for futility erroneously (scenario **A**).

For peer review only - http://bmjopen.bmj.com/site/about/guidelines.xhtml

STOP OHSS

STOP-OHSS Simulation Report Final Version 2.0

7 Summary

The trial will require a maximum total of 224 participants (112 per arm) to maintain a power of 90% for a 2.5% one-sided assuming a 41% hospitalisation rate in the usual care and a large targeted reduction of 20%. One interim analysis will be performed to inform whether the trial should be stopped early for futility when 65% of the maximum total sample size had accrued primary outcome data. It has been shown that the trial will be sufficiently powered across realistic event rates (e.g., 29% to 50%) as long as the targeted effect of a 20% reduction is realistic. Key limitations of the design are highlighted in Section 8.

In this trial, it has been shown in Figure 1 that the choice of futility stopping threshold had a negligible effect on the power and type I error rate of the study across the usual care event rate considered. However, as evident in Figure 2, the choice of futility stopping threshold has a significant effect on the chances of correctly stopping for futility and a very small effect on the probability of erroneously stopping early for futility.

The choice of a **4.5% hospital reduction as a futility stopping threshold appears very reasonable** for four specific reasons. First, it is below the lower bound of hospital reduction likely to result in cost savings. Second, it gives a higher chance of stopping the trial early when then the treatment is known to be ineffective which is the motivation for futility early stopping. Third, the risk of erroneously stopping the trial early for futility when the treatment is known to be effective is very small. Finally, it does not affect the power of the study.

The final decision on which futility stopping threshold to use lies with the research team guided by the results presented and the levels of risk they are willing to tolerate. Notably, in this design, the chosen futility stopping threshold is advisory so it can be overruled or ignored by the independent data monitoring committee and research team without affecting the design negatively.

Finally, the group sequential design considered here is feasible to implement and the primary outcome is observed within 28 days of randomisation and recruitment in this population is expected to be very slow.

8 Limitations

One major limitation of the design is that it is based on a large treatment effect of 20% hospital reduction which is viewed as realistic (based on data from observational studies) and worthwhile to change practice. However, as noted from the updated health economic model, hospital reductions of about 4.7% to 5.7% are also likely to result in cost savings. As a result, there is a region where the treatment effect is likely to be cost-saving (e.g., 10% to 15%) but the trial will be severely underpowered for such small to moderate treatment effects. This will complicate the interpretation of results if such effects turn out to be true. Finally, the treatment effect of 20% becomes unrealistic and impossible to achieve as the usual care event rate gets closer to 20%, however, the hospitalisation rate in the usual care is likely to be much higher than 20% based on the current best estimate presented in Section 2.2.

For peer review only - http://bmjopen.bmj.com/site/about/guidelines.xhtml

9 Impact of early stopping on study duration and sample size saving

An audit review of medical records suggested that the average recruitment across 20 sites will be around 4.6 patients per month. Stopping the trial early for futility at an interim analysis implies that an additional total of 78 participants would no longer be recruited. The trial will not be paused during interim analysis and interim decision-making process as the recruitment rate is expected to be very slow. As such, even if the trial is stopped early, we would expect negligible participants to have been recruited during interim analysis (e.g., at most 2 participants per arm). At this point, if we assume a constant average recruitment rate of 4.6 patients per month across all sites and staggered opening of sites to recruitment starting with 2 followed by 5, 8, 11, 15, 18, and 20 in consecutive months, then 10 months of further recruitment will no longer be required. Finally, potential cost savings can be extrapolated under different scenarios.

10 Contributions

Munya Dimairo designed the simulation study, wrote the simulation code, conducted simulations, and drafted the simulation report.

Laura Sutton performed statistical quality control in R relating to results presented in Table 1.

Ellen Lee performed statistical quality control in Stata relating to the simulation code.

Tracey Young contributed to the preliminary health economic model that informed the futlity stopping threshold.

David White and Cara Mooney reviewed the report and contributed to Section 9.

11 Acknowledgements

We are very grateful for the invaluable feedback from the Trial Steering Committee independent statistician (Babak Choodari-Oskooei, Medical Research Council (MRC) Clinical Trial Unit (CTU) at University College London (UCL)). Finally, we would like to thank the Trial Management Group (TMG) responsible for the day-to-day conduct of the trial.

12 References

1. Wassmer, G. & Pahlke, F. RPACT Package Overview | RPACT.

For peer review only - http://bmjopen.bmj.com/site/about/guidelines.xhtml

STANDARD PROTOCOL ITEMS: RECOMMENDATIONS FOR INTERVENTIONAL TRIALS

SPIRIT 2013 Checklist: Recommended items to address in a clinical trial protocol and related documents*

Section/item	Item No	Description	Addressed on page number
**Administrative information**			
Title	1	Descriptive title identifying the study design, population, interventions, and, if applicable, trial acronym	1
Trial registration	2a	Trial identifier and registry name. If not yet registered, name of intended registry	3 & 19
	2b	All items from the World Health Organization Trial Registration Data Set	
Protocol version	3	Date and version identifier	1
Funding	4	Sources and types of financial, material, and other support	20
Roles and responsibilities	5a	Names, affiliations, and roles of protocol contributors	1 & 19
	5b	Name and contact information for the trial sponsor	1
	5c	Role of study sponsor and funders, if any, in study design; collection, management, analysis, and interpretation of data; writing of the report; and the decision to submit the report for publication, including whether they will have ultimate authority over any of these activities	NA
	5d	Composition, roles, and responsibilities of the coordinating centre, steering committee, endpoint adjudication committee, data management team, and other individuals or groups overseeing the trial, if applicable (see Item 21a for data monitoring committee)	14

For peer review only

Introduction

Background and rationale	6a	Description of research question and justification for undertaking the trial, including summary of relevant studies (published and unpublished) examining benefits and harms for each intervention	____5-6______
	6b	Explanation for choice of comparators	____5-6______
Objectives	7	Specific objectives or hypotheses	____6________
Trial design	8	Description of trial design including type of trial (eg, parallel group, crossover, factorial, single group), allocation ratio, and framework (eg, superiority, equivalence, noninferiority, exploratory)	____6________

Methods: Participants, interventions, and outcomes

Study setting	9	Description of study settings (eg, community clinic, academic hospital) and list of countries where data will be collected. Reference to where list of study sites can be obtained	_____7________
Eligibility criteria	10	Inclusion and exclusion criteria for participants. If applicable, eligibility criteria for study centres and individuals who will perform the interventions (eg, surgeons, psychotherapists)	____8-9_______
Interventions	11a	Interventions for each group with sufficient detail to allow replication, including how and when they will be administered	___9-10_______
	11b	Criteria for discontinuing or modifying allocated interventions for a given trial participant (eg, drug dose change in response to harms, participant request, or improving/worsening disease)	_____10_______
	11c	Strategies to improve adherence to intervention protocols, and any procedures for monitoring adherence (eg, drug tablet return, laboratory tests)	_____10_______
	11d	Relevant concomitant care and interventions that are permitted or prohibited during the trial	____10_______
Outcomes	12	Primary, secondary, and other outcomes, including the specific measurement variable (eg, systolic blood pressure), analysis metric (eg, change from baseline, final value, time to event), method of aggregation (eg, median, proportion), and time point for each outcome. Explanation of the clinical relevance of chosen efficacy and harm outcomes is strongly recommended	____11________
Participant timeline	13	Time schedule of enrolment, interventions (including any run-ins and washouts), assessments, and visits for participants. A schematic diagram is highly recommended (see Figure)	____Figure 1____

For peer review only - http://bmjopen.bmj.com/site/about/guidelines.xhtml

Sample size	14	Estimated number of participants needed to achieve study objectives and how it was determined, including clinical and statistical assumptions supporting any sample size calculations	____13_______
Recruitment	15	Strategies for achieving adequate participant enrolment to reach target sample size	____7-8______

Methods: Assignment of interventions (for controlled trials)

Allocation:			
Sequence generation	16a	Method of generating the allocation sequence (eg, computer-generated random numbers), and list of any factors for stratification. To reduce predictability of a random sequence, details of any planned restriction (eg, blocking) should be provided in a separate document that is unavailable to those who enrol participants or assign interventions	_____9_____
Allocation concealment mechanism	16b	Mechanism of implementing the allocation sequence (eg, central telephone; sequentially numbered, opaque, sealed envelopes), describing any steps to conceal the sequence until interventions are assigned	____9______
Implementation	16c	Who will generate the allocation sequence, who will enrol participants, and who will assign participants to interventions	____9______
Blinding (masking)	17a	Who will be blinded after assignment to interventions (eg, trial participants, care providers, outcome assessors, data analysts), and how	___13-14_____
	17b	If blinded, circumstances under which unblinding is permissible, and procedure for revealing a participant's allocated intervention during the trial	___NA_______

Methods: Data collection, management, and analysis

Data collection methods	18a	Plans for assessment and collection of outcome, baseline, and other trial data, including any related processes to promote data quality (eg, duplicate measurements, training of assessors) and a description of study instruments (eg, questionnaires, laboratory tests) along with their reliability and validity, if known. Reference to where data collection forms can be found, if not in the protocol	_____11-12____
	18b	Plans to promote participant retention and complete follow-up, including list of any outcome data to be collected for participants who discontinue or deviate from intervention protocols	____10______

For peer review only - http://bmjopen.bmj.com/site/about/guidelines.xhtml

Data management	19	Plans for data entry, coding, security, and storage, including any related processes to promote data quality (eg, double data entry; range checks for data values). Reference to where details of data management procedures can be found, if not in the protocol	______18_______
Statistical methods	20a	Statistical methods for analysing primary and secondary outcomes. Reference to where other details of the statistical analysis plan can be found, if not in the protocol	____15-17_____
	20b	Methods for any additional analyses (eg, subgroup and adjusted analyses)	____15-17_____
	20c	Definition of analysis population relating to protocol non-adherence (eg, as randomised analysis), and any statistical methods to handle missing data (eg, multiple imputation)	_____16_______
**Methods: Monitoring**			
Data monitoring	21a	Composition of data monitoring committee (DMC); summary of its role and reporting structure; statement of whether it is independent from the sponsor and competing interests; and reference to where further details about its charter can be found, if not in the protocol. Alternatively, an explanation of why a DMC is not needed	____14_________
	21b	Description of any interim analyses and stopping guidelines, including who will have access to these interim results and make the final decision to terminate the trial	____14-15______
Harms	22	Plans for collecting, assessing, reporting, and managing solicited and spontaneously reported adverse events and other unintended effects of trial interventions or trial conduct	_____13________
Auditing	23	Frequency and procedures for auditing trial conduct, if any, and whether the process will be independent from investigators and the sponsor	____18_________
**Ethics and dissemination**			
Research ethics approval	24	Plans for seeking research ethics committee/institutional review board (REC/IRB) approval	____19________
Protocol amendments	25	Plans for communicating important protocol modifications (eg, changes to eligibility criteria, outcomes, analyses) to relevant parties (eg, investigators, REC/IRBs, trial participants, trial registries, journals, regulators)	______7________

Consent or assent	26a	Who will obtain informed consent or assent from potential trial participants or authorised surrogates, and how (see Item 32)	______8________
	26b	Additional consent provisions for collection and use of participant data and biological specimens in ancillary studies, if applicable	______NA________
Confidentiality	27	How personal information about potential and enrolled participants will be collected, shared, and maintained in order to protect confidentiality before, during, and after the trial	____18______
Declaration of interests	28	Financial and other competing interests for principal investigators for the overall trial and each study site	____20________
Access to data	29	Statement of who will have access to the final trial dataset, and disclosure of contractual agreements that limit such access for investigators	____18_________
Ancillary and post-trial care	30	Provisions, if any, for ancillary and post-trial care, and for compensation to those who suffer harm from trial participation	_______NA_______
Dissemination policy	31a	Plans for investigators and sponsor to communicate trial results to participants, healthcare professionals, the public, and other relevant groups (eg, via publication, reporting in results databases, or other data sharing arrangements), including any publication restrictions	____19________
	31b	Authorship eligibility guidelines and any intended use of professional writers	______19________
	31c	Plans, if any, for granting public access to the full protocol, participant-level dataset, and statistical code	______NA_______
**Appendices**			
Informed consent materials	32	Model consent form and other related documentation given to participants and authorised surrogates	______Supp_______
Biological specimens	33	Plans for collection, laboratory evaluation, and storage of biological specimens for genetic or molecular analysis in the current trial and for future use in ancillary studies, if applicable	_______NA_______

*It is strongly recommended that this checklist be read in conjunction with the SPIRIT 2013 Explanation & Elaboration for important clarification on the items. Amendments to the protocol should be tracked and dated. The SPIRIT checklist is copyrighted by the SPIRIT Group under the Creative Commons "Attribution-NonCommercial-NoDerivs 3.0 Unported" license.

BMJ Open

Outpatient paracentesis for the management of Ovarian HyperStimulation Syndrome: Study protocol for the STOP-OHSS randomised controlled trial

Journal:	*BMJ Open*
Manuscript ID	bmjopen-2023-076434.R1
Article Type:	Protocol
Date Submitted by the Author:	09-Nov-2023

Complete List of Authors:	White, David; The University of Sheffield, Clinical Trials Research Unit, School of Health and Related Research<br>Pye, Clare; Sheffield Teaching Hospitals NHS Foundation Trust, Jessop Wing<br>Ridsdale, Katie; The University of Sheffield, Clinical Trials Research Unit, School of Health and Related Research<br>Dimairo, Munyaradzi; The University of Sheffield, Clinical Trials Research Unit, School of Health and Related Research<br>Mooney, Cara; The University of Sheffield, Clinical Trials Research Unit, School of Health and Related Research<br>Wright, Jesssica; The University of Sheffield, Clinical Trials Research Unit, School of Health and Related Research<br>Young, Tracey; The University of Sheffield, School of Health and Related Research<br>Cheong, Ying; University of Southampton, Obstetrics and Gynaecology<br>Drakeley, Andrew; Liverpool Women's NHS Foundation Trust, Hewitt Fertility Centre<br>Mathur, Raj; Manchester University NHS Foundation Trust, Manchester Academic Health Sciences Centre<br>O'Cathain, Alicia; University of Sheffield, School of Health and Related Research<br>Desoysa, Lauren; The University of Sheffield, Clinical Trials Research Unit, School of Health and Related Research<br>Sizer, Anya; Fertility Network UK<br>Lumley, Elizabeth; The University of Sheffield<br>Chatters, Robin; The University of Sheffield, Clinical Trials Research Unit, School of Health and Related Research<br>Metwally, Mostafa; Sheffield Teaching Hospitals NHS Foundation Trust; The University of Sheffield

**Primary Subject Heading**:	Obstetrics and gynaecology
Secondary Subject Heading:	Reproductive medicine
Keywords:	Reproductive medicine < GYNAECOLOGY, GYNAECOLOGY, REPRODUCTIVE MEDICINE

SCHOLARONE™
Manuscripts

Title:

Outpatient paracentesis for the management of Ovarian HyperStimulation Syndrome: Study protocol for the STOP-OHSS randomised controlled trial

David White[a], Clare Pye[b], Katie Ridsdale[a], Munya Dimairo[a], Cara Mooney[a], Jesssica Wright[a], Tracey Young[c], Ying Cheong[d], Andrew Drakeley[e], Raj Mathur[f], Alicia O'Cathain[c], Lauren Desoysa[a], Anya Sizer[g], Elizabeth Lumley[c], Robin Chatters[a], Mostafa Metwally[b,c]

a – Clinical Trials Research Unit, School of Health and Related Research, The University of Sheffield
b – Jessop Fertility, Sheffield Teaching Hospitals NHS Foundation Trust
c – School of Health and Related Research, The University of Sheffield
d – Complete Fertility Southampton, Princess Anne Hospital, Human Development and Health, Faculty of Medicine, University of Southampton
e – Hewitt Fertility Centre, Liverpool Women's NHS Foundation Trust
f – Manchester Academic Health Sciences Centre, Manchester Foundation Trust
g - Fertility Network UK

Corresponding author

Mr David White
Clinical Trials Research Unit (CTRU)
School of Health and Related Research (ScHARR)
The University of Sheffield
Email: d.a.white@sheffield.ac.uk

Sponsor
Sheffield Teaching Hospital NHS Foundation Trust
The University of Sheffield
D Floor, Royal Hallamshire Hospital
Glossop Road
Sheffield
S10 2JF

Key words
Assisted conception
Randomised Controlled trial
Ovarian HyperStimulation Syndrome
Paracentesis
In Vitro Fertilisation

Protocol Version 4 Dated 12 January 2023

Word count: 4347

For peer review only - http://bmjopen.bmj.com/site/about/guidelines.xhtml

Abstract

Introduction

Ovarian hyperstimulation syndrome (OHSS) is the most significant short-term complication of pharmacological ovarian stimulation. Symptoms range from mild abdominal discomfort to rare complications such as renal failure, thromboembolism, and respiratory distress syndrome.

Currently, clinical practice typically involves monitoring the patient until the condition becomes severe, at which point they are admitted to hospital, where drainage of ascitic fluid (paracentesis) may take place. Preliminary studies have indicated that earlier outpatient paracentesis may reduce the progression of OHSS and prevent hospitalisation in women.

Methods and analysis

This United Kingdom, multi-centre, pragmatic, two-arm, parallel-group, adaptive (group sequential with one interim analysis), open-label, superiority, confirmatory, group sequential, individually randomised controlled trial, with internal pilot will assess the clinical and cost-effectiveness and safety of outpatient paracentesis versus conservative management (usual care) for moderate or severe OHSS. 224 women from 20 National Health Service & Private fertility units will be randomised (1:1) and followed up for up to 13.5 months. The primary outcome is the rate of OHSS related hospital admission of at least 24 hours within 28 days post-randomisation. The primary analysis will be intention to treat with difference in hospitalisation rates as measure of treatment effect. Secondary outcomes include time to resolution of symptoms, patient satisfaction, adverse events and cost-effectiveness. A qualitative sub-study will facilitate the feasibility of recruitment. Participant recruitment commenced in June 2022.

Ethics & Dissemination

London - Southeast Research Ethics Committee approved the protocol (reference: 22/LO/0015). Findings will be submitted to peer- reviewed journals and abstracts to

For peer review only

For peer review only - http://bmjopen.bmj.com/site/about/guidelines.xhtml

relevant national and international conferences, as well as being disseminated to trial participants and patient groups.

Trial Registration number: ISRCTN - 71978064.

Strengths and Limitations of this study

- This is the largest randomised controlled trial to date which aims to establish whether outpatient paracentesis reduces the rate of OHSS related hospital admissions in those presenting with moderate or severe OHSS, compared to usual care.
- Due to the nature of the intervention, it is not possible to blind study participants or clinicians.
- Measures are taken to reduce the impact of this, including a secondary independent blinded adjudication of hospital admissions and blinding of trial statisticians
- Patients with lived experience were involved in the design of the trial and will provide advice throughout delivery

Introduction

Ovarian hyperstimulation syndrome (OHSS) is the most significant short-term complications of pharmacological ovarian stimulation for assisted conception. It is most often seen in women with a high ovarian reserve, who are prone to an excessive response to gonadotropin stimulation.

There is no universally accepted classification of OHSS. In the UK, the Royal College of Obstetricians and Gynaecologists (RCOG) classification is widely used and includes mild, moderate, severe, or critical categories[3]. Moderate cases involve the build-up of fluid in the abdomen (called ascites), increased ovarian size, and abdominal distention and discomfort. In severe cases, fluid retention and dehydration can lead to changes to the constitution of the blood (haemoconcentration and hypoalbuminemia); critical cases can lead to respiratory distress, thrombosis, disturbed renal and liver functions, and rarely death (1).

For peer review only

OHSS can be further classified into early and late (Table 1). Early OHSS is usually caused by the ovarian stimulation drugs given during treatment and usually occurs within 7 days after the final drug Human chorionic gonadotropin (hCG) is given. Late OHSS usually occurs 10 days or more after the administration of hCG and is caused by endogenous hCG of the resulting pregnancy. The late type is usually more difficult to control, runs a longer course and is more severe (2).

Table 1. Categorisation of OHSS and its severity, based on the RCOG definitions[3]

**Categorisation of OHSS**	
Early OHSS	Caused by the ovarian stimulation drugs given during Assisted Reproductive Technologies and occurs usually up to 7 days of the final trigger drug (hCG) being given.
Late OHSS	Usually occurs 10 or more days after the trigger drug is given.
Where a patient presents at day 8 or 9 clinical judgement will be used to classify the early or late OHSS.	
**Severity of OHSS**	
Mild OHSS	• Abdominal bloating<br>• Mild abdominal pain<br>• Ovarian size is usually below 8cm
Moderate OHSS	Patients do not meet the criteria of severe (described below) and have fluid accumulation in abdomen (ascites), confirmed by ultrasound scan. The following symptoms may also be present:<br>• Moderate abdominal pain<br>• Increased ovarian size of usually 8-12cm<br>• Nausea with or without vomiting<br><br>*Some degree of clinical judgement may be needed to confirm the diagnosis of moderate OHSS.*
Severe OHSS	Patients have fluid accumulation in abdomen (clinical ascites/clinically detectable fluids), confirmed by ultrasound, with or without hydrothorax<br>The main distinguishing features are any of the following:<br>• Low urine output (oliguria)(< 300 ml/day or < 30 ml/hour)<br>• Haematocrit >0.45, (confirmed via full blood count (FBC) test)<br>• Ovarian size >12cm.
Critical OHSS	Clinically obvious ascites, with one of the following features:<br>the patient has a tense ascites or a large hydrothorax<br>• Haematocrit level >0.55 (confirmed via FBC test)**<br>• A white cell count of over 25000/ml (confirmed via FBC test)<br>• Anuria (very little/no urine) (< 100 ml/day)<br>• Thromboembolism<br>• Acute respiratory distress syndrome<br><br>*** haematocrit level is the most important measurement*

Clinical practice usually involves monitoring the patient until the condition becomes severe, when the patient is admitted to hospital, as recommended by the RCOG guideline (3). Inpatient management often includes drainage of ascitic fluid (paracentesis) which can result

For peer review only - http://bmjopen.bmj.com/site/about/guidelines.xhtml

in a significant improvement of the condition and in renal blood fluid, urine output, and reversal of the haematological abnormalities (4–6). Complications such as venous thromboembolism can have long-term health problems lasting well beyond the length of the pregnancy.

Although mild forms are fairly common (approximately 1 in 3 women undergoing IVF (In Vitro Fertilisation) treatment), more severe OHSS (up to 8% for combined moderate and severe OHSS) (3) can have a significant impact on a woman's health resulting in prolonged hospitalisation and posing a significant economic burden on both patient and National Health Service (NHS). With over 69,000 IVF cycles performed in the United Kingdom (UK) in 2019 alone(7), the burden of the problem becomes evident (3). It is therefore important to look into novel approaches to improve outcomes and the management of complications associated with IVF (8).

Small retrospective studies (9,10) and a larger uncontrolled study (11) have suggested that transvaginal paracentesis can reduce the need for hospitalisation to between 2.9% and 14% of patients. Another small cohort study identified patients with moderate OHSS at risk of this progressing to severe OHSS and reduced this progression with only 1 to 3 paracentesis procedures (12). There is also some evidence to suggest transabdominal administration of paracentesis can prevent inpatient hospitalisation compared to those managed supportively (13). Furthermore, preliminary studies have safely and effectively managed patients in the outpatient setting by using a pigtail catheter to drain ascetic fluid (14,15). All of these studies have still achieved high pregnancy rates of 68% (13) and 100% (12). Modelling also suggests that it would be more cost-effective to treat patients with moderate or severe OHSS with early outpatient paracentesis (OP), compared to less active management and inpatient admission (16).

Preliminary results have been encouraging and suggest promising safety and effectiveness of OP (9–13,15,17). However, this needs to be evaluated in a robust and adequately powered randomised controlled trial (RCT) taking into consideration variabilities in practice across fertility units.

For peer review only - http://bmjopen.bmj.com/site/about/guidelines.xhtml

This study aims to establish the clinical and cost-effectiveness, safety, and acceptability of OP as an active management for women with moderate or severe OHSS. The primary objective is to establish whether OP reduces the rate of OHSS related hospital admissions in those presenting with moderate or severe OHSS compared to usual care. Secondary objectives are to:

1) Establish whether OP prevents the escalation of OHSS severity;
2) Establish whether OP reduces the time taken for OHSS symptoms to resolve;
3) Establish the safety of OP as an active intervention for moderate or severe OHSS;
4) Explore whether OP would improve patient satisfaction and quality of life;
5) Establish whether OP is cost-effective by examining healthcare resource use and patient costs;
6) Facilitate the feasibility of conducting the RCT by identifying problems with the conduct of the RCT during an internal pilot.

Pre-trial development work involved qualitative interviews with clinicians and patients exploring the feasibility and acceptability of earlier active management protocols. These results fed into a consensus event to confirm the treatment protocol used in the RCT. We also undertook a comprehensive audit of proposed RCT centres to inform the parameters used to design the RCT.

Method and analysis

Full detailed methods of the STOP OHSS trial are included in the trial protocol, available via the trial registry (https://www.isrctn.com/ISRCTN71978064).

STOP-OHSS is a pragmatic, parallel open-label, multi-centre, superiority, adaptive, group sequential, confirmatory RCT with an internal pilot (after 15 of 31 months recruitment) to assess feasibility aspects (Figure 1). Eligible participants will be individually randomised (1:1) to receive either routine treatment or OP plus daily diary monitoring.

The trial is a collaboration between The Jessop Wing, Sheffield Teaching Hospital NHS Foundation Trust & the University of Sheffield - Clinical Trials Research Unit (CTRU) who are responsible for the conduct of the trial. It is funded by the National Institute of Health and

For peer review only - http://bmjopen.bmj.com/site/about/guidelines.xhtml

Care Research (NIHR) Health Technology Assessment (HTA), who had no involvement in the study design or conduct; or the decision to submit the protocol for publication. The trial will be conducted in compliance with the protocol, Good Clinical Practice and regulatory requirements. Substantial protocol amendments will be communicated to all relevant bodies. Main trial recruitment commenced June 2022 and is ongoing.

Internal pilot

An internal pilot will use the same procedures as the main trial and will assess the following after 15 months of a planned 31-month recruitment period:

1) Participant recruitment (both arms),
2) Retention of randomised participants (both arms),
3) Delivery of the OP treatment (only OP arm).

At the end of the pilot phase, the Trial Steering Committee (TSC) will report to the funder on whether criteria have been met to continue.

Recruitment

The trial will recruit women attending approximately 20 NHS or private UK Fertility Units, undergoing Assisted Reproductive Technologies (including IVF, IntraCytoplasmic Sperm Injection and Intrauterine Insemination) and who experience moderate or severe, early, or late OHSS symptoms. Participation is voluntary and choosing not to participate will not negatively influence the woman's treatment. Consent can be withdrawn at any stage. Women will be identified by the following recruitment strategies.

Monitoring cases – women who are considered at risk of developing moderate or severe OHSS due to various risk factors such as a previous episode of OHSS, high response to stimulation with increased follicular activity, >15 eggs with or without symptoms of OHSS, polycystic ovary syndrome, high levels of Anti-Müllerian hormone (greater than 30 pmol/l, younger women (<30yrs) who have a good ovarian reserve and are therefore known to respond well to treatment, low body mass index or any symptoms of OHSS like bloating and nausea. Incident cases – women with moderate or severe OHSS who were not previously identified as at risk.

For peer review only - http://bmjopen.bmj.com/site/about/guidelines.xhtml

Potentially eligible participants will be approached as soon as possible after the diagnosis of moderate or severe OHSS and, where possible, initial eligibility screening has taken place. Women may also be alerted to the trial via the trial website or posters displayed at the fertility unit.

Research staff will provide the patient with the participant information sheet and/or link to a trial animation video outlining the study as soon as they become aware the woman is potentially eligible. Prior to randomisation, full written informed consent (see Supplementary File 1) will be obtained by a suitably trained clinician researcher at the clinic visit. Standard consent includes a provision for anonymised data to be shared with other researchers. Baseline data will be collected at this visit and participants will be randomised.

Women will be considered suitable if they meet the following eligibility criteria.
Inclusion criteria:

1. Women presenting with moderate or severe, early or late OHSS,
2. Patients able and willing to attend weekly follow-up appointments in person or remotely, daily remote appointments/phone calls, and able to undertake self-monitoring at home.

Exclusion criteria:

1. OHSS-related exclusion criteria:
    a. Significant pain or vomiting requiring hospitalisation,
    b. Pulmonary embolism,
    c. When in the judgment of the clinician, the patient's condition is severe enough to warrant admission to a High Dependency Care Unit (such as Critical OHSS as defined in the RCOG green-top guidelines) and therefore not suitable for outpatient management.
2. Non-OHSS related medical conditions: a concurrent medical condition requiring immediate inpatient management,
3. Patients who have been previously randomised but later present with moderate or severe OHSS symptoms in subsequent cycles after their initial trial involvement,

For peer review only

4. Participation in other trials involving ovarian stimulation or ovarian response.

Centralised Research Nursing Delivery Team

Local research teams will be supported in recruiting participants and ongoing management of the trial protocol procedures from the Central Research Nursing Delivery team based at the Jessop Wing, Sheffield Teaching Hospitals NHSFT. This team will support local discussions with potential participants about trial participation, randomisation procedures, and monitoring and routine follow up visits in both trial arms.

Randomisation and concealment

Patients will be randomised using a centralised web-based system (SCRAM) hosted by Sheffield CTRU, which has user-restricted functionalities granting access rights to specific areas as appropriate. The sequence will be computer generated using permuted block randomisation stratified by recruiting site and severity of OHSS. Block sizes will be disclosed during dissemination of findings and only the randomisation (trial) statistician(s) not involved in the recruitment process will know the block sizes during the trial. The trial statistician will generate the randomisation sequence; however, they will not have access to the generated sequence. Research staff at recruiting centres will use a web-based computer system with restricted access rights to enter participant details; randomisation outcome will then be revealed. Re-randomisation will not be permitted.

Intervention and control

Following randomisation, women in the intervention arm will have OP performed in the outpatient setting as soon as clinically possible. Sites will follow their own local procedures for conducting paracentesis either abdominally or vaginally by a suitably trained Doctor, Advanced Nurse Practitioner or Radiologist in the fertility, gynaecology, or radiology unit and while the trial does not dictate how to perform the intervention, guidance will be offered.

If re-hydration is required via intravenous methods this will be at the discretion of the attending clinician. If a large volume of fluid is removed, then intravenous colloid therapy should be considered. If this administration of fluid results in overnight inpatient hospitalisation it will not be viewed as meeting the trials primary outcome relating to

For peer review only - http://bmjopen.bmj.com/site/about/guidelines.xhtml

For peer review only

hospitalisation. Some patients may require multiple drainage which can be performed if the participant has a re-accumulation of ascites.

Women in the usual care (UC) group will not have the paracentesis performed in an OP setting and will be monitored by their clinical team as per usual care. If usual care at the site involves administering paracentesis on an outpatient basis for treatment of OHSS then the site will be requested to stop routinely providing this for participants in the usual care arm.

Both groups of women will be provided with access to a diary (either in paper format or sent electronically using Research Electronic Data Capture (REDCap)(18) hosted at The University of Sheffield for the purpose of daily self-report of weight, abdominal girth, diarrhoea, vomiting, pain, shortness of breath, fluid input, and urine output. This information will be used by the clinical team in the intervention arm on a daily basis to inform further management and whether to perform further OP. The UC group will also perform daily self-monitoring, but this data will be sent directly to CTRU for input into the database and will not be available to be reviewed by site staff.

Some fertility units undertake other preventative measures (e.g., dopamine agonists, Gonadotropin-releasing hormone (GnRH) antagonists, cessation of IVF cycle) to reduce the risk of OHSS and details of these measures will be collected during the trial, along with information on the provision of care within the conservative management arm. Rarely, patients may be prescribed GnRH antagonists as treatment for established moderate or severe OHSS. This will not be prohibited for trial participants in both arms and data will be collected on GnRH use within the trial.

Participants may wish to stop study treatment, or there may be a clinical need to stop treatment. Participants randomised to the intervention arm, who withdraw from receiving the intervention will continue to be followed-up and will remain in the trial, unless they request to be withdrawn.

Outcomes

For peer review only - http://bmjopen.bmj.com/site/about/guidelines.xhtml

Data collection points are outlined in Table 2.

Primary outcome is any:

- OHSS-related hospitalisation for at least 24 hours within 28 days of randomisation.

Secondary outcomes:

- Need for OHSS related hospitalisation within 28 days – independent blinded central assessment,
- Time to resolution of OHSS assessed within 28 days of randomisation,
- Progression of OHSS severity within 28 days of randomisation,
- Cumulative length of OHSS related hospitalisation,
- Live birth, pregnancy outcomes, neonatal death and serious adverse events (SAEs) including congenital abnormalities in the new-born within 13.5 months of randomisation,
- The occurrence of thrombosis, embolism and significant infections requiring antibiotic treatment or hospitalisation within 90 days of randomisation,
- Adverse events (AEs) within 28 days of randomisation,
- Patient satisfaction assessed using the Client Satisfaction Questionnaire 8 (CSQ-8)(19) based on total scores at 28 days post randomisation,
- EQ-5D-5L participant quality of life daily and at 28 days post randomisation,
- Health resource use and patient costs at 28 days post randomisation.

For peer review only

Table 2. Data collection time-points

	Baseline/ randomisation (Day 0)	Daily until symptom resolution	Weekly (Days 7, 14, 21, 28)	Symptom Resolution	Symptom Deterioration[#]	During hospitalisation	Day 90	13.5 months
**Data collected by site staff**								
Demographics and medical history (including medication to prevent OHSS)	OPt							
Symptoms:<br>- Fluid balance including fluid drunk, urine output*<br>- Diarrhoea and vomiting<br>- Nausea**<br>- Abdominal girth<br>- Pain<br>- Shortness of breath<br>- Weight	OPt	SR	T/OP^	OPt	OPt	IP / MN		
Blood tests (FBC) – if a face-to-face appointment	OPt		T/OP^	OPt	OPt	IP / MN		
Training and provision of consumables, and record details	OPt							
EQ-5D-5L questionnaire	OPt	SR				SR / IP		
OHSS related hospital admission within 28 days (primary outcome) and reasons for hospitalisation			T/OPt	OPt	OPt			
Intervention delivery (volume of ascites removed, number of paracentesis)			MN	OPt		MN		
Conservative management arm – details of usual care monitoring			MN	OPt				
Use of GnRH antagonist for treatment of OHSS			OPt	OPt	OPt	IP / MN		
Confirmation of symptom resolution				OPt		MN		
Adverse events (patient)		T***	OPt / MN	OPt	OPt	IP		
Pregnancy outcome (e.g miscarriage) and ongoing pregnancies							MN	
Incidence of thrombosis or embolism or							MN	

For peer review only - http://bmjopen.bmj.com/site/about/guidelines.xhtml

significant infection								
Live birth information and pregnancy outcome (e.g. miscarriage)								MN
Neonatal death, and SAEs related to the baby								MN
**Data collected by the central study team**								
Health resource use questionnaire			Q (day 28 only)					
CSQ-8 questionnaire			Q (day 28 only)					
Patient cost questionnaire			Q (day 28 only)					
EQ-5D-5L questionnaire			Q (day 28 only)					

OPt = Outpatient

IP = Inpatient

T = Data collected over the telephone by a research nurse

SR = Self-reported by the patient

MN = Medical notes

If symptoms deteriorate to the point where hospital admission is thought to be required

^ If OHSS symptoms have not resolved by this time point

Q = data collected via a questionnaire

For peer review only - http://bmjopen.bmj.com/site/about/guidelines.xhtml

Follow up

Data will be collected as follows:

- Participant diary data, self-completed daily until symptom resolution.
- Monitoring visits, either face-to-face or remotely, at days 7, 14, 21 and 28 or until symptoms have resolved.
- Symptom resolution visit, face-to-face (unless symptoms resolve during hospitalisation and the data can be collected from medical notes or the participant)
- A symptom deterioration visit, to assess if hospitalisation may be required.
- A participant self-completed questionnaire at 28 days
- Remote follow up at day 90, to collect ongoing pregnancies/pregnancy outcome (e.g., miscarriage), thrombosis/embolism and significant infections. Participants not pregnant at this timepoint will be considered complete and will not require further data collection.
- For participants identified as pregnant at 90 days, live birth and pregnancy outcome (e.g., miscarriage) as well as neonatal death, congenital abnormalities, and serious adverse events for the baby at 13.5 months.

Safety considerations, safety monitoring and adverse event reporting

All AEs and SAEs will be recorded at each participating fertility unit. All AEs/SAEs will be followed up until satisfactory resolution or until the treating clinician and the principal investigator deems the event to be chronic or the participant to be stable. Clinical/research staff will ask participants for any details of AEs at each daily contact in the intervention arm and then in both groups at all timepoints.

Blinding

It will not be possible to blind participants or clinicians to treatment allocation. The trial statistician and TSC will be blinded. To assess the potential impact of outcome assessment bias, an additional analysis of the primary endpoint will be performed, using an independent blinded adjudicated primary outcome concerning whether trial participants needed OHSS-related hospitalisation within 28 days.

Trial monitoring and oversight

For peer review only - http://bmjopen.bmj.com/site/about/guidelines.xhtml

The trial will be overseen by a TSC and Data Monitoring and Ethics Committee (DMEC). Membership of both will consist of independent experts in the field and the TSC includes a patient representative. The DMEC may advise the chair of the TSC at any time if, in their view, the trial should be stopped for ethical or adaptive reasons.

Day-to-day running of the trial will be coordinated by the Trial Management Group (TMG), consisting of the grant co-applicants, plus members of the Jessop Wing Fertility Unit, Sheffield CTRU, and patient representatives.

Sample size and interim analyses

Under current conservative management, the mean OHSS-related hospitalisation rate is approximately 41.2% (26/63) based on a retrospective audit of medical records of IVF patients across six IVF fertility units over a year [95% confidence interval (CI): 29.0% to 54.4%). Earlier active management interventions based on uncontrolled small previous studies have resulted in low hospitalisation rates of around 0-8% (10,12,13,20). As such, this trial is targeting a 20% absolute reduction in hospitalisation for OP to be considered superior to usual care. This reduction is believed to be realistic as previous uncontrolled studies have observed similar or greater effects and, if observed, is more convincing to change clinical practice. A 20% absolute reduction translates to an odds ratio (OR) of 0.3825 or relative risk/risk ratio (RR) of 0.512 assuming a 41% usual care event rate. Thus, the targeted effect in terms of OR or RR is a function of the usual care event rate whereas the targeted 20% absolute reduction is consistent across the plausible usual care event rates. An updated preliminary health economics model (trial protocol, via ISRCTN71978064) has indicated that OP needs to achieve at least 4.7% to 5.7% reduction in hospitalisation to become cost-saving under several assumptions about the ratio of early to late OHSS and underlying usual care hospitalisation rates assuming a 20% targeted reduction.

The aim of the trial is to gather convincing evidence to influence the effects of the early OP that is most likely to change practice regardless of the direction of results and so a group sequential design with an option for early stopping for futility (lack of benefit) is used.

The trial will require a maximum total sample size of ~224 (112 per arm) with an interim analysis when ~146 participants (73 per arm) have accrued primary outcome. This assumes a 90% power, one-sided 2.5% type 1 error, 41% usual care hospitalisation rate, 0% dropout

For peer review only - http://bmjopen.bmj.com/site/about/guidelines.xhtml

rate, a 20% reduction in hospitalisation rate, and a futility threshold of -4.5% (difference in hospitalisation rate). In this case, the trial will be stopped early for futility if the observed reduction in hospitalisation rate at an interim analysis is less than 4.5%. There is only a 1.9% chance of stopping the trial early for futility in error when OP is truly beneficial. Finally, there is a 72.2% probability of stopping early when the effect of OP is the same as usual care (i.e., 41% hospitalisation rate) (see Supplementary File 2)

Statistical analysis

Detailed of all analysis will be described in an open-access and pre-specified SAP to be developed and signed off before accessing unblinded data.

The primary analysis will be intention-to-treat, including all eligible participants randomised with informed consent. Baseline data will be summarised at the interim and final analyses by treatment group. The absolute difference in hospitalisation rates between arms is the primary summary measure of the treatment effect. RR and OR (estimated using a simple logistic regression model) will also be presented. At an interim analysis, the unadjusted difference in hospitalisation rates between arms will be calculated to inform the interim decision on whether to stop early for futility. For sensitivity analysis, a mixed effects logistic regression model adjusted for stratification factors (site as a random effect and OHSS severity as a fixed effect) and adjusted difference in hospitalisation rates will be obtained using the delta method via margins (30). For the final analysis of the primary outcome, the unadjusted difference in hospitalisation rates with CI obtained via normal approximation will be reported as well as the p-value from a Chi-squared test. Stagewise ordering will be used for sensitivity analysis to obtain the median unbiased estimate of the difference in hospitalisation rates between arms with the associated 95% CI to be presented alongside the maximum likelihood estimate if the trial progressed beyond interim analysis. The details of additional sensitivity analyses to be performed adjusting for baseline covariates are found in the SAP. Reporting will adhere to the Adaptive designs CONSORT Extension (ACE) guidance (21).

For progression of OHSS severity, a mixed effects logistic regression model adjusted for stratification factors (site as a random effect and OHSS severity as a fixed effect)

For peer review only

For peer review only - http://bmjopen.bmj.com/site/about/guidelines.xhtml

will be used and an adjusted difference in hospitalisation rates will be obtained using the delta method via margins (30). Treatment effects will be presented as adjusted odds ratio (aOR), adjusted risk difference (aRD) and adjusted relative risk/risk ratio with 95% CIs.

For time to resolution of OHSS assessed within 28 days post-randomisation, Kaplan-Meier curves will be used to visualise the resolution curves between treatment arms and differences qualified using a log-rank test. Median time to resolution by treatment arm with 95% CI will be calculated and reported as summary measures of within-group effects. Participants who fail to achieve resolution of symptoms within 28 days post-randomisation will be censored. Sensitivity analysis And alternative approaches when the proportional hazard assumption is not met will be detailed in the SAP.

For cumulative length of OHSS hospital stay, bootstrapping resampling procedure (accounting for stratification) will be used to obtain the median difference with 95% CI and associated p-value. For patient satisfaction at 28 days, a total satisfaction score will be analysed using a mixed effects linear regression model adjusted for site (as a random effect) and OHSS severity (as a fixed effect), and the adjusted mean difference between treatment arms with 95% CI and associated p-value will be presented.

Analysis of safety outcomes (e.g., AEs and SAEs) will be based on descriptive statistics using the safety analysis population defined based on treatment-as-received population. Details of additional analysis accounting for repeated events and differential follow-up is found in the SAP.

Subgroup analyse will be performed to explore whether there is heterogeneity in treatment effect on the primary outcome across the following prespecified subgroups:
1) Baseline severity of OHSS (moderate or severe),
2) Whether a participant is taking a preventative drug at randomisation (yes or no),
3) Whether the participant has early or late OHSS at randomisation,
4) Whether the OP procedure was done vaginally or abdominally. For participants who received multiple procedure (which may happen in few cases), this classification will be based on the first performed procedure.

For peer review only - http://bmjopen.bmj.com/site/about/guidelines.xhtml

A mixed effects logistic regression model that includes an interaction effect between pre-planned subgroup and treatment group adjusted for site (as a random effect) and severity of OHSS (as a fixed effect) only if it is not a subgroup factor of interest will be used.

Health Economics

Analyses will be conducted in conjunction with a health economic analysis plan (HEAP). Two HEAPs will be produced, one in relation to the interim analysis, and one relating to the end-of-trial analysis.

The primary cost-effectiveness analysis will present cost per hospitalisation avoided. The feasibility of conducting a cost-utility analysis will be explored in the feasibility study, and if viable, the results will also be expressed as incremental cost per quality-adjusted life years (QALY) gained. An interim health economic analysis will be performed when 65% of the maximum sample size has accrued. The aims of the interim analysis will be to update the pre-trial model and to examine the cost-effectiveness of OP compared to usual care. The QALY will be calculated using the EQ-5D-5L questionnaire (22) administered daily until resolution of symptoms and then at 28 days post randomisation, when women will also be asked to complete resource use questionnaires and a patient cost questionnaire about their last visit to their fertility doctor. Further resource use data will be collected on training of staff and participants on the trial and consumables given; details of monitoring given to participants; outpatient treatment delivery; hospital admission information and treatment received. Unit costs will be derived from appropriate national sources including NHS reference costs and Personal Social Service Research Unit costs (23,24).

Analyses will be undertaken from the NHS and personal social service perspective as recommended by the National Institute for Health and Care Excellence (25) and will follow recommended methods and good practice guides (26,27). The primary analysis for both Sub-Protocols within the trial will be a within-trial analysis using data from the trial. A secondary analysis will consider a decision tree model, similar to those used by Casals et al., and Csokmay et al. (16,28), that extends costs and outcomes over a 12 month time horizon. Incremental differences between costs and effectiveness/QALYs between those receiving

For peer review only - http://bmjopen.bmj.com/site/about/guidelines.xhtml

For peer review only

OP and those receiving usual care will be described and the incremental cost effectiveness ratio will be calculated. Sensitivity analyses and subgroup analyses will also be undertaken.

Qualitative study to aid recruitment

A qualitative study will facilitate the feasibility of conducting the RCT by identifying potential problems with recruitment to the RCT, so that solutions can be instigated rapidly. We will:

1) Identify optimal and sub-optimal practice for recruitment to this trial using audio recording of recruitment sessions

2) Gather information about numbers approached and numbers who consent by assessment of anonymised recruitment logs and other trial documentation

3) Identify problems with the recruitment process by interviewing recruiting healthcare professionals

4) Explore the experiences of women being recruited by interviewing both women who consented, and women who declined, to participate in the trial.

Data Management and monitoring

Data management, confidentiality and access, and monitoring were undertaken in line with Sheffield CTRU SOPs. Details can be found in the full study protocol, accessible via the study webpage: (https://www.sheffield.ac.uk/scharr/research/centres/ctru/stop-ohss).

Data access requests will be reviewed by a sub-committee of the TMG while the trial is on-going and by the Sheffield CTRU in conjunction with project collaborators after the trial has ended.

Ethics and dissemination

The study is registered on the ISRCTN database (reference: 71978064) and has been approved by the London - Southeast Research Ethics Committee (reference: 22/LO/0015). The findings of this trial will be submitted to peer- reviewed journals and abstracts to national and international conferences. Other stakeholder specific outputs in relevant formats will be produced for trial participants, commissioners, IVF practitioners, third sector and user advocacy organisations.

For peer review only

Patient and public involvement

People with experience of OHSS were involved in the design and development of this project. Patient participation was incorporated in the delivery of the project through representation in the TMG and TSC. The Jessop Wing Reproductive Health Public Advisory Panel contributed to the development of the protocol and study materials.

Acknowledgements

We thank The Jessop Wing Reproductive Health Public Advisory Panel, each local Principal Investigator, Research Nurse/Midwife and research delivery team in the recruitment and management of patients and all members of the central project management team at Sheffield CTRU.

Authors Contributions

Contributors: MM, DW, CP, MD, TY, YC, AD, RM, AO, RC, & AS conceived the study and contributed to the study design. MM, DW, CP, KR, MD, CM, JW, TY, YC, AD, RM, AO, LD & AS initiated the project, developed the protocol, and contributed to implementation. DW, CP, KR, & MM drafted the manuscript. All authors read and approved the final manuscript. MM is the guarantor.

Funding statement

This article presents independent research supported by the NIHR HTA programme [NIHR128137]. The views expressed are those of the author(s) and not necessarily those of the NHS, the NIHR or the Department of Health.

Competing interests

RM declares a private practice in fertility treatment.

Data availability statement

Deidentified participant data and statistical code will be made available upon reasonable request. Requests should be made via email to ctru@sheffield.ac.uk, stating the data fields required and purpose of the request (ideally with a protocol but, at a minimum, with a

For peer review only - http://bmjopen.bmj.com/site/about/guidelines.xhtml

research plan). The data dictionary and statistical analysis plan can also be made available. Requests will be considered on a case-by-case basis and requestors will be asked to complete a data sharing agreement with the sponsor before data transfer.

For peer review only

References

1. Boothroyd C, Karia S, Andreadis N, Rombauts L, Johnson N, Chapman M. Consensus

For peer review only - http://bmjopen.bmj.com/site/about/guidelines.xhtml

statement on prevention and detection of ovarian hyperstimulation syndrome. Aust New Zeal J Obstet Gynaecol. 2015 Dec;55(6):523–34.

2. NHS Digital. Hospital Episode Statistics, Admitted Patient Care - England, 2010-11. 2011.

3. Royal College of Obstetricians and Gynaecologists. RCOG Green-top Guideline No. 5: The Management of Ovarian Hyperstimulation Syndrome. London; 2016.

4. Levin I, Almog B, Avni A, Baram A, Lessing JB, Gamzu R. Effect of paracentesis of ascitic fluids on urinary output and blood indices in patients with severe ovarian hyperstimulation syndrome. Fertil Steril. 2002;77(5):986–8.

5. Qublan HS, Al-Taani MI, Megdadi MF, Metri RM, Al-Ahmad N. Multiple transvaginal ascitic fluid aspirations improves the clinical and reproductive outcome in patients undergoing in vitro fertilisation treatment complicated by severe early ovarian hyperstimulation syndrome. J Obstet Gynaecol (Lahore). 2012 May;32(4):379–82.

6. Chen CD, Yang JH, Chao KH, Chen SU, Ho HN, Yang YS. Effects of repeated abdominal paracentesis on uterine and intraovarian haemodynamics and pregnancy outcome in severe ovarian hyperstimulation syndrome. Hum Reprod. 1998 Aug;13(8):2077–81.

7. Fertility treatment 2019: trends and figures | HFEA. 2021.

8. Audit Office N. Reducing emergency admissions A picture of the National Audit Office logo.

9. Smith LP, Hacker MR, Alper MM. Patients with severe ovarian hyperstimulation syndrome can be managed safely with aggressive outpatient transvaginal paracentesis. Fertil Steril. 2009 Dec;92(6):1953–9.

10. Lincoln SR, Opsahl MS, Blauer KL, Black SH, Schulman JD. Aggressive outpatient treatment of Ovarian Hyperstimulation Syndrome with ascites using transvaginal culdocentesis and intravenous albumin minimizes hospitalization. J Assist Reprod Genet. 2002;19(4):159–63.

11. Gustofson RL, Browne P, Van Nest RL, Richter KS, Larsen FW. Aggressive Outpatient Management of Severe Ovarian Hyperstimulation Syndrome Avoids Complications

For peer review only - http://bmjopen.bmj.com/site/about/guidelines.xhtml

and Prolonged Disease Course. Fertil Steril. 2005 Sep;84:S95.

12. Fluker MR, Copeland JE, Yuzpe AA. An ounce of prevention: Outpatient management of the ovarian hyperstimulation syndrome. Fertil Steril. 2000 Apr;73(4):821–4.

13. Shrivastav P, Nadkarni P, Craft I. Day care management of severe ovarian hyperstimulation syndrome avoids hospitalization and morbidity. Hum Reprod. 1994 May;9(5):812–4.

14. Abuzeid MI, Nassar Z, Massaad Z, Weiss M, Ashraf M, Fakih M. Pigtail catheter for the treatment of ascites associated with ovarian hyperstimulation syndrome. Hum Reprod. 2003 Feb;18(2):370–3.

15. Abuzeid M, Warda H, Joseph S, Corrado MG, Abuzeid Y, Ashraf M, et al. Outpatient Management of Severe Ovarian Hyperstimulation Syndrome (OHSS) with Placement of Pigtail Catheter. Facts, Views Vis ObGyn. 2014;6(1):31.

16. Csokmay JM, Yauger BJ, Henne MB, Armstrong AY, Queenan JT, Segars JH. Cost analysis model of outpatient management of ovarian hyperstimulation syndrome with paracentesis: "Tap early and often" versus hospitalization. Fertil Steril. 2010 Jan;93(1):167–73.

17. Gebril A, Hamoda H, Mathur R. Outpatient management of severe ovarian hyperstimulation syndrome: a systematic review and a review of existing guidelines. https://doi.org/101080/1464727320171331048. 2017 Apr;21(2):98–105.

18. Harris PA, Taylor R, Thielke R, Payne J, Gonzalez N, Conde JG. Research electronic data capture (REDCap)—A metadata-driven methodology and workflow process for providing translational research informatics support. J Biomed Inform. 2009 Apr;42(2):377–81.

19. Larsen DL, Attkisson CC, Hargreaves WA, Nguyen TD. Assessment of client/patient satisfaction: Development of a general scale. Eval Program Plann. 1979;2(3):197–207.

20. Lainas GT, Kolibianakis EM, Sfontouris IA, Zorzovilis IZ, Petsas GK, Tarlatzi TB, et al. Outpatient management of severe early OHSS by administration of GnRH antagonist in the luteal phase: an observational cohort study. Reprod Biol Endocrinol.

For peer review only - http://bmjopen.bmj.com/site/about/guidelines.xhtml

2012;10(1):69.

21. Dimairo M, Pallmann P, Wason J, Todd S, Jaki T, Julious SA, et al. The adaptive designs CONSORT extension (ACE) statement: a checklist with explanation and elaboration guideline for reporting randomised trials that use an adaptive design. Trials 2020 211. 2020 Jun;21(1):1–34.

22. Van Hout B, Janssen MF, Feng YS, Kohlmann T, Busschbach J, Golicki D, et al. Interim scoring for the EQ-5D-5L: Mapping the EQ-5D-5L to EQ-5D-3L value sets. Value Heal. 2012 Jul;15(5):708–15.

23. Curtis, Lesley A. and Burns A. Unit costs of health and social care 2019. Canterbury. 2020;

24. NHS England » National Cost Collection for the NHS.

25. Excellence NI for H and C. Guide to the methods of technology appraisal 2013. 2013.

26. Faria R, Gomes M, Epstein D, White IR. A Guide to Handling Missing Data in Cost-Effectiveness Analysis Conducted Within Randomised Controlled Trials. Pharmacoeconomics. 2014 Dec;32(12):1157–70.

27. Manca A, Hawkins N, Sculpher MJ. Estimating mean QALYs in trial-based cost-effectiveness analysis: the importance of controlling for baseline utility. Health Econ. 2005 May;14(5):487–96.

28. Casals G, Fábregues F, Pavesi M, Arroyo V, Balasch J. Conservative medical treatment of ovarian hyperstimulation syndrome: a single center series and cost analysis study. Acta Obstet Gynecol Scand. 2013 Jun;92(6):686–91.

For peer review only - http://bmjopen.bmj.com/site/about/guidelines.xhtml

Figure legend

Figure 1. Study flow diagram.

For peer review only

For peer review only - http://bmjopen.bmj.com/site/about/guidelines.xhtml

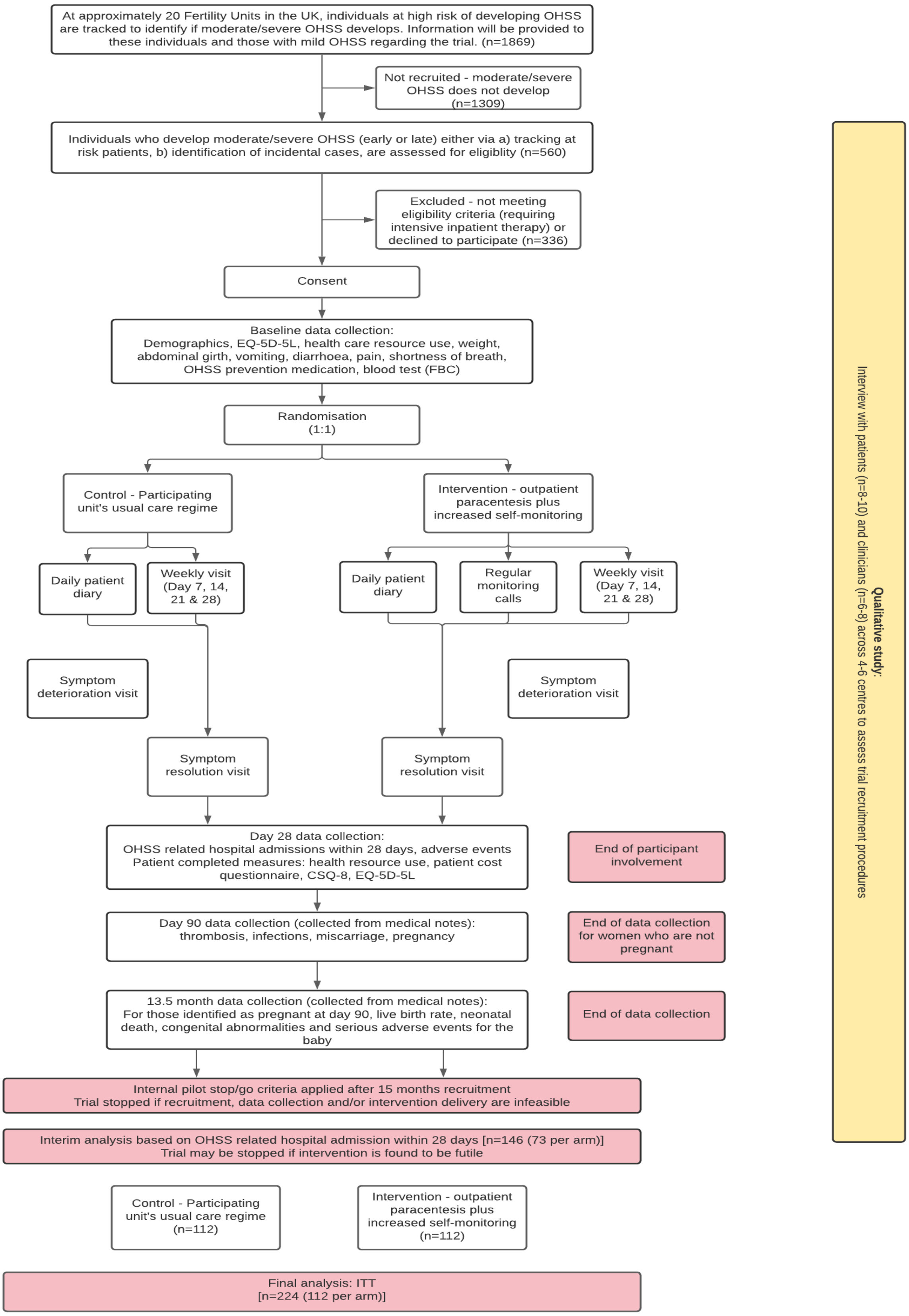
At approximately 20 Fertility Units in the UK, individuals at high risk of developing OHSS are tracked to identify if moderate/severe OHSS develops. Information will be provided to these individuals and those with mild OHSS regarding the trial. (n=1869)
Not recruited - moderate/severe OHSS does not develop (n=1309)
Individuals who develop moderate/severe OHSS (early or late) either via a) tracking at risk patients, b) identification of incidental cases, are assessed for eligiblity (n=560)
Excluded - not meeting eligibility criteria (requiring intensive inpatient therapy) or declined to participate (n=336)
Consent
Baseline data collection: Demographics, EQ-5D-5L, health care resource use, weight, abdominal girth, vomiting, diarrhoea, pain, shortness of breath, OHSS prevention medication, blood test (FBC)
Randomisation (1:1)
Control - Participating unit's usual care regime
Intervention - outpatient paracentesis plus increased self-monitoring
Daily patient diary
Weekly visit (Day 7, 14, 21 & 28)
Daily patient diary
Regular monitoring calls
Weekly visit (Day 7, 14, 21 & 28)
Symptom deterioration visit
Symptom deterioration visit
Symptom resolution visit
Symptom resolution visit
Day 28 data collection: OHSS related hospital admissions within 28 days, adverse events Patient completed measures: health resource use, patient cost questionnaire, CSQ-8, EQ-5D-5L
End of participant involvement
Day 90 data collection (collected from medical notes): thrombosis, infections, miscarriage, pregnancy
End of data collection for women who are not pregnant
13.5 month data collection (collected from medical notes): For those identified as pregnant at day 90, live birth rate, neonatal death, congenital abnormalities and serious adverse events for the baby
End of data collection
Internal pilot stop/go criteria applied after 15 months recruitment Trial stopped if recruitment, data collection and/or intervention delivery are infeasible
Interim analysis based on OHSS related hospital admission within 28 days [n=146 (73 per arm)] Trial may be stopped if intervention is found to be futile
Control - Participating unit's usual care regime (n=112)
Intervention - outpatient paracentesis plus increased self-monitoring (n=112)
Final analysis: ITT [n=224 (112 per arm)]
Qualitative study: Interview with patients (n=8-10) and clinicians (n=6-8) across 4-6 centres to assess trial recruitment procedures

Research site NHS logo

The University Of Sheffield.

STOP OHSS

STOP-OHSS (Shaping and Trialling Outpatient Protocols for Ovarian HyperStimulation Syndrome):

A randomised controlled trial to assess the clinical and cost-effectiveness of active management of Ovarian HyperStimulation Syndrome.

PATIENT INFORMATION SHEET

Protocol Number: STH20532
ISRCTN Number: 71978064
Sponsor: Sheffield Teaching Hospitals NHS Foundation Trust
Clinical Research Office Sheffield
Royal Hallamshire Hospital
D Floor
Glossop Road
Sheffield, S10 2JF
Chief Investigator: Mostafa Metwally

Local Principal Investigator: *[insert name]*

Research Nurse: *[insert name]*
Telephone: *[insert]*
Email: *[insert]*

Introduction

We would like to invite you to take part in our research trial. Before you make a decision, it is important for you to understand why the research is being done and what it will involve. Please take time to read the following information carefully and discuss it with friends and family if you wish to do so. Ask us if there is anything that is not clear or if you would like more information. Take time to decide whether or not you wish to take part. Thank you for reading this.

For peer review only - http://bmjopen.bmj.com/site/about/guidelines.xhtml

The University Of Sheffield.

Research site NHS logo

What is our research about?

Ovarian hyperstimulation syndrome (OHSS) is a condition that can affect women having fertility treatment.

OHSS can be caused by the medication taken to increase egg production during ovulation induction or In Vitro Fertilisation (IVF). The medication known as Follicle-stimulating hormone (FSH) is a hormone which can cause too many eggs to develop, and your ovaries can become large and painful. It can affect women who are still recovering from the effects of this medication given during their fertility treatment and also those who have become pregnant after treatment.

If OHSS occurs up to 7 days after receiving the medication, it is classed as '**Early** OHSS' and if it occurs after 10 or more days, in patients who have become pregnant after treatment, due to their body naturally producing more of the same hormone which can cause the ovaries to become large, is classed as '**Late** OHSS'.

OHSS can range in severity, and can be categorised in the following ways:

- **Mild OHSS** – mild abdominal swelling, discomfort and nausea.
- **Moderate OHSS** – symptoms of mild OHSS, with increased swelling because fluid builds up in the abdomen. This can cause abdominal pain and vomiting.
- **Severe OHSS** – symptoms of moderate OHSS with extreme thirst and dehydration. You may only pass small amounts of urine which is dark in colour and/or you may experience difficulty breathing because of a build-up of fluid in your abdomen. A serious, but rare, complication is a blood clot (thrombosis) forming in the legs or lungs.

Mild symptoms are common and usually resolve quickly, but for some women who experience moderate or severe OHSS these symptoms can worsen. Severe OHSS can lead to serious health complications needing treatment in hospital for several days or weeks, and in a small number of women can be life threatening.

Currently, fertility teams monitor symptoms of OHSS and intervene if the symptoms get worse and the woman needs to be admitted to hospital for intensive monitoring or

For peer review only - http://bmjopen.bmj.com/site/about/guidelines.xhtml

The University Of Sheffield.

Research site NHS logo

treatment. This treatment can include a procedure call paracentesis and is normally performed as an inpatient.

The aim of this trial is to understand whether taking action earlier by carrying out the same paracentesis procedure but as an **outpatient** and including additional monitoring can stop symptoms getting worse and prevent women having to be admitted to hospital.

About the procedure

Paracentesis is a procedure where a needle is inserted through the vagina or abdomen to drain the fluid that has collected to relieve the OHSS symptoms and is carried out in a similar way to egg collection. It is generally a safe procedure and will not affect your pregnancy if you are already pregnant. Paracentesis is already regularly carried out at many hospitals for patients who are admitted for treatment. Potential complications include feeling tired, dizzy or lightheaded, increased pain in your tummy, low blood pressure or bleeding from the drainage site but these are short term symptoms and will be managed by your clinical team during the procedure. There is also the possibility of infection and injury to major blood vessels or bowel, but these are extremely rare. We will collect data about these side effects, and they will be monitored by the research team. In this study the procedure will be performed by your doctor/nurse/radiologist in an **outpatient** setting to prevent the OHSS symptoms getting worse. It can feel uncomfortable like egg collection, and you will be given pain relief.

What will happen if I take part in the trial?

Women taking part in the trial will be randomly allocated (randomised) by a computer to participate in one of two groups:

Group A) the intervention group will have the procedure called paracentesis (drainage of fluid) which will take place in an **outpatient** setting. They will have extra monitoring from their fertility team which will include filling in a daily patient diary.

Group B) the control group will not have the paracentesis procedure as an outpatient and will be monitored by their fertility team in the usual way, but will be asked to fill in a daily patient diary.

For peer review only - http://bmjopen.bmj.com/site/about/guidelines.xhtml

Research site NHS logo

If you decide to take part this means that neither you nor your doctors can decide which treatment you will have and there is an equal chance of being placed in treatment group A or B.

What happens to you if you are randomised to the intervention group?

Your doctor will perform the paracentesis procedure as an **outpatient**. The procedure involves putting a thin needle or tube into your abdomen or vagina, depending on how your hospital usually does this, to remove the fluid which has built up. You may be offered a local anaesthetic for this procedure and we will ask you to stay in clinic for a few hours after the procedure to make sure you feel well enough to go home. Depending on how much fluid is in your abdomen, your doctor will drain the fluid then take out the needle or insert a tube which will be attached to a small drainage bag to collect the fluid from your abdomen. The bag can then be attached to your leg and hidden discreetly under a skirt, dress or loose fitting trousers and, your team will explain how to empty it once you get home. It will be taken out when no more fluid is draining which can take about 2 weeks, but your doctor will discuss which is best for you before carrying out the procedure.

We will ask you to fill in an electronic daily diary recording any OHSS symptoms you have. This will include your weight, how much fluid you have been drinking, how much urine you have passed, whether or not you have any pain or breathlessness and we will also ask you to measure your abdominal girth (the width of your tummy). We will give you a tape measure, weighing scales and measuring jug to do this. A member of the clinical team from your hospital will call you and discuss this diary information with you until you have recovered from the OHSS. This will be daily for the first few days at least but may then be less often. Depending on your symptoms you may be asked to visit the hospital for review by your doctor to give a blood sample or to have more fluid drained by the same **outpatient** paracentesis procedure.

The trial is designed to monitor your symptoms from home and only bring you into the hospital if necessary. For safety reasons we may ask you to visit the hospital weekly, if necessary, during the first 28 days that you are taking part in the trial or if your symptoms start to get worse or have got better, but again this will be discussed with you.

For peer review only - http://bmjopen.bmj.com/site/about/guidelines.xhtml

The University Of Sheffield.

What happens to you if you are randomised to the control group?

Exactly the same will happen as described above but you will not have the paracentesis procedure as an outpatient. However, you may be contacted less frequently by the clinical team, in line with the usual care provided at your centre, this may be every few days, but this will be discussed with you. If your doctor decides based on the information you have given during these calls that you need treatment for your OHSS symptoms, then you will be admitted to hospital as an inpatient. You might still have paracentesis performed but it will be as an inpatient on the ward and not as an outpatient.

Travel expenses for visits to the hospital for trial purposes will be reimbursed (e.g., parking, public transport).

Who is carrying out the research?

This trial is being sponsored (led) by the Sheffield Teaching Hospitals NHS Foundations Trust. *[insert participating site name]* is taking part in the trial as one of the research centres.

Why have I been chosen and what are the possible benefits?

You have been invited to take part in this trial because you have developed moderate or severe OHSS. Taking part in this trial may or may not reduce your chances of going into hospital because of OHSS, but information from this trial may help doctors understand whether it would be helpful to offer this **outpatient** procedure to women who have moderate or severe OHSS in the future.

We plan to recruit 224 women who are suffering from moderate or severe OHSS over 3 years. It will take place in about 20 fertility units in the UK. Half of these women will receive the paracentesis procedure as an **outpatient** and have increased monitoring, and half will not.

Do I have to take part?

No. It is up to you to decide whether or not to take part. If you do decide to take part you will be given this information sheet to keep and will be asked to sign a consent form. If you decide to take part you can still decide to withdraw at any time and without giving a reason.

If you decide to withdraw at any time, or not to take part, this will not affect the standard of care you receive. If you withdraw from the trial, any anonymised information we have

For peer review only - http://bmjopen.bmj.com/site/about/guidelines.xhtml

The University Of Sheffield.

Research site NHS logo

already collected about you will be included in the final data analysis, but we will not collect further information without your consent.

Providing information and data as part of the trial

All women, regardless of which group you are in, will be followed up to see how long it takes for your symptoms to get better and whether hospital admission is needed. We will also compare the safety, acceptability and cost of each treatment. We will ask you to complete questionnaires about your health, how you felt about the service you received and any health care you needed 28 days after you gave consent to take part in the trial. These can be completed by post or online. We will also ask you to complete a daily diary as described in the above section "**What happens to you if you are randomised to the intervention group?**".

You will be contacted by telephone by a research nurse/midwife or DR, or asked to come into hospital for the following reasons:

- A weekly visit (up to 28 days or until your symptoms have got better)
- If your symptoms start to get worse
- Once your symptoms have resolved

During these visits, the doctor/ nurse will ask you some questions about your symptoms. Information will also be collected from your medical notes about your symptoms and details of any time spent in hospital because of OHSS.

If you are taken into hospital during the trial at any other NHS Trust, this information may also be requested from them. We will ask your permission to do this.

We would also like to review your medical notes around 3 months (90 days) after you agree to take part in the trial to collect any information about any infections or blood clots (thrombosis) that you may have experienced. At this point we will also collect any information about whether you are pregnant or any pregnancy outcome. If you are not pregnant we will not collect any further data from your notes. If, however, we find that you are pregnant we will review your notes again around 10 months later (13.5 months after you first joined the trial) to collect information about the outcome of your pregnancy.

For peer review only

For peer review only - http://bmjopen.bmj.com/site/about/guidelines.xhtml

The University Of Sheffield.

Research site NHS logo

The research team will compare how many people were admitted to hospital from both groups at the end of the trial. This will help us to work out whether early paracentesis as an **outpatient** reduces the chance of women suffering from moderate or severe OHSS being taken into hospital.

For peer review only

For peer review only - http://bmjopen.bmj.com/site/about/guidelines.xhtml

The University Of Sheffield.

Research site NHS logo

Summary of what will happen if you take part in this trial

All patients

If you agree to take part you will be asked to sign a consent form. A computer programme will randomly allocate you to the intervention group or to the control group

We will collect some information about you, including: demographic information, height, weight, abdomen size, possible blood tests, and questions on your symptoms. You will be given a 'daily diary' to record your symptoms.

Intervention Group

You will receive the paracentesis procedure as an outpatient (this may occur more than once)

A nurse/doctor from your hospital will call you to discuss your 'daily diary'. These calls will be daily at first but may then be less often, until you have recovered.

Control Group

You will not have the paracentesis procedure unless your symptoms worsen. If this occurs, it will take place in hospital as an inpatient.

You will be contacted at the amount normal to your hospital. You should complete your 'daily diary' and hand it in when visiting the clinic.

You will receive face-to-face or remote **weekly** monitoring contacts. The nurse/doctor will discuss your symptoms and collect some information including your weight, abdominal girth, fluid input/output, and possible blood samples.

If you get better, you will come to the clinic for some measurements. If your symptoms get worse, you will come to clinic to be checked, and have some information collected. If you are hospitalised, a nurse may visit you to collect some information.

After **28 days**, you will be asked to complete some questionnaires. Your doctor/nurse will collect information from your medical records about your symptoms and treatment but we may need to contact you about whether you were hospitalised.

We will check your medical notes at **3 months** to collect some information, and if we find out you are pregnant we may collect information from your medical records for up to **13.5 months**. We will not need to contact you.

For peer review only - http://bmjopen.bmj.com/site/about/guidelines.xhtml

The University Of Sheffield.

What are the possible disadvantages and risks of taking part?

Paracentesis is a relatively safe procedure, but there are some risks, which have been described in the section '**About the procedure**', and these should be discussed with your doctor/ nurse.

Additionally, completing the patient diary daily for 28 days may be an inconvenience to you. This includes measurements of weight, abdominal girth, fluid intake and urine output. For patients in the intervention group, we will use this information to monitor your condition as best as possible. The data that both groups provide in these diaries will help us to answer our research question. We will explain how you should collect the data and provide you with any equipment needed.

How will we use information about you?

Sheffield Teaching Hospitals NHS Foundation Trust (STH NHSFT) is the sponsor that is leading this trial and will act as the data controller. This means that they are responsible for looking after your information and using it properly. The trial is managed by the Clinical Trials Research Unit (CTRU) in the School of Health and Related Research at The University of Sheffield.

Together STH NHSFT and the CTRU will need to use information from you and your medical records for this trial. This information will include your consent to take part in the trial, initials, NHS number (or CHI number if in Scotland), name, contact details and date of birth. STH NHSFT and the CTRU will use this information to do the research or to check your records to make sure that the research is being done properly. Members of the research team who do not need to know who you are will not be able to see your name or contact details. Your data will have a code number instead.

We will keep all information about you safe and secure.

STH NHSFT, the CTRU and *[insert site name]* will keep your data securely for 15 years after the end of the trial.

No one would be able to work out that you took part in the trial from the reports we write about it.

For peer review only - http://bmjopen.bmj.com/site/about/guidelines.xhtml

The University Of Sheffield.

Research site NHS logo

What are your choices about how your information is used?

You can stop being part of the trial at any time, without giving a reason, but we will keep information about you that we already have, as it may affect our results.

- If you choose to stop taking part in the trial, we would like to continue collecting information about your health from your hospital records. If you do not want this to happen, please let us know and we will stop.
- We need to manage the information that we collect in specific ways for the research to be reliable. This means that we won't be able to let you see or change the data we hold about you.
- If you agree to take part in this trial, you may have the option to take part in future research using your data saved from this trial.
- The anonymised data may be used to support other research in the future, and may be shared with other researchers for comparison studies

Where can you find out more about how your information is used?

You can find out more about how we use your information here www.sheffieldclinicalresearch.org/for-patients-public/how-is-yourinformation-handled-in-research/

What will happen to the results of the research trial?

The results of this trial will be published in scientific journals and presented at scientific meetings, as well as shared directly with NHS Trusts, professional bodies and patient groups. You will not be identified in any report or publication. If you would like to receive a summary of the trial results and a copy of the research report, we will send this to you.

Who is sponsoring and funding the research?

This trial is funded by the National Institute for Health Research (NIHR) Health Technology Assessment (HTA) and sponsored by Sheffield Teaching Hospitals NHS Foundation Trust.

It is organised, managed and coordinated by the University of Sheffield, and data will be collected and stored by this institution.

For peer review only - http://bmjopen.bmj.com/site/about/guidelines.xhtml

Additionally, the trial will be supervised on a regular basis by a Data Monitoring and Ethics Committee (DMEC) and a Trial Steering Committee (TSC). The primary function of the DMEC is to ensure the absolute safety of all participants.

Who has reviewed the trial?

Research in the NHS is looked at by an independent group of people called a Research Ethics Committee to protect your interests. This trial has been reviewed and given a favourable opinion by the London - South East Research Ethics Committee.

The trial has also been reviewed by the Reproductive Health Research Public Advisory Panel at the Jessop Wing, Sheffield. This is a group of lay members whose main purpose is to ensure that research carried out is patient focused. The role of the panel is to give a patients' perspective on the research that is being carried out.

What if I wish to complain about the way in which this trial has been conducted?

If you have a concern about any aspect of this trial you should ask to speak with the researchers who will do their best to answer your questions. If you remain unhappy and wish to complain formally, you can do this through the NHS complaints procedure, by contacting the Patient Liaison Service (PALs).

Patient Advice and Liaison Service:

Telephone: 0151 702 4353

Email: PALS@lwh.nhs.uk

Harm

Your participation in this trial is to help us find out if performing this procedure in women suffering from moderate or severe OHSS reduces the chance of being admitted to hospital. In the event that something does go wrong, and you are harmed during the research, and this is due to someone's negligence then you may have grounds for a legal action for compensation, but you may have to pay your legal costs. The normal National Health Service complaints procedure will still be available to you.

Further information and contact details for local research team

You are free to ask questions about this information sheet or the trial now or at any time

For peer review only - http://bmjopen.bmj.com/site/about/guidelines.xhtml
For peer review only

The University Of Sheffield.

Research site NHS logo

during the trial. If you experience any side effects, or if you have any questions about this research during this trial you may contact: kirsty.mckendrick@sheffield.ac.uk

The trial website contains information that may be helpful. The website is located at www.sheffield.ac.uk/scharr/research/centres/ctru/stop-ohss. If you agree to participate in this trial, you will receive a signed and dated copy of the consent form and this patient information sheet for your records. Whether or not you wish to participate in our trial, we would like to thank you for taking the time to read this information sheet.

For peer review only

For peer review only - http://bmjopen.bmj.com/site/about/guidelines.xhtml

The University Of Sheffield.

Research site NHS logo

STOP OHSS

STOP-OHSS (Shaping and Trialling Outpatient Protocols for Ovarian HyperStimulation Syndrome):

A randomized controlled trial to assess the clinical and cost-effectiveness of active management of Ovarian HyperStimulation Syndrome.

CONSENT FORM

Local Principal Investigator: *[insert name]*
Centre Number/ID:
Participant Identification Number:

Please initial

1. I can confirm that I have read and understood the participant information sheet version 3.0 dated 12Dec22 for the STOP-OHSS trial and have had the opportunity to ask questions and these have been answered satisfactorily. (Your initials)

2. I understand that my participation in the trial is voluntary and I am free to withdraw at any time without my treatment or legal rights being affected. (Your initials)

3. I understand that my local research team and the study organisers at the University of Sheffield (Clinical Trials Research Unit) will each have access to personal information about my progress and may receive a copy of my consent form for monitoring purposes. Transfer of this information between organisations will be done in confidence via secure email or a secure access controlled database. (Your initials)

4. I understand that the information collected will be used for medical research only and that I will not be identified in any way in the analysis and reporting of the results. (Your initials)

5. I understand that even if I withdraw from the trial, information already collected about me may be included in the final analysis after being anonymised. (Your initials)

For peer review only - http://bmjopen.bmj.com/site/about/guidelines.xhtml

The University Of Sheffield.

Research site NHS logo

When completed: 1 for participant; 1 for researcher site file; 1 to be kept in medical notes

6. I understand that relevant sections of my medical notes and data collected during the trial may be looked at by members of the research team (at Sheffield Teaching Hospitals NHSFT or the Clinical Trials Research Unit at the University of Sheffield), or my participating trial centre, or by individuals from statutory regulators. I give permission for these individuals to have access to my records. ☐ (Your initials)

7. I understand that if I am hospitalised during my time in the trial at an NHS Trust or a private centre other than Sheffield Teaching Hospitals NHSFT then the research team will seek to obtain data about the hospitalisation from the other organisation for the purpose of this study. I give my permission for this. ☐ (Your initials)

8. I understand that information held by the NHS may be used to keep in touch with me to follow up on my health and pregnancy status for the purpose of the trial. ☐ (Your initials)

9. I understand that if I become pregnant during the course of the trial that information about the outcome of my pregnancy including relevant sections of my child's medical notes (if applicable) may be looked at by members of the research team (at Sheffield Teaching Hospitals NHSFT or the Clinical Trials Research Unit at the University of Sheffield) or Sheffield Teaching Hospitals participating trial centre, or by individuals from statutory regulators. I give permission for these individuals to have access to my child's records. ☐ (Your initials)

10. I understand that by agreeing to take part in this study I also agree to completing trial visits and follow-up questionnaires. The Sheffield CTRU will be given my contact details in order to send these. ☐ (Your initials)

11. I understand that data collected about me without personal identifiers may be used to support other research in the future, and may be shared with other researchers at other institutions to enable them to undertake studies for which my data may be useful; and I give permission for this. ☐ (Your initials)

12. I understand the information that I have been given about the above study and I agree to take part. ☐ (Your initials)

The following are optional and additional, answering "No" to any will not affect your ability to take part in the study.

When completed: 1 for participant; 1 for researcher site file; 1 to be kept in medical notes

For peer review only - http://bmjopen.bmj.com/site/about/guidelines.xhtml

The University Of Sheffield.

Research site NHS logo

	Yes		No
13. I understand that if I choose to stop taking part in the study, the research team would like to continue collecting information about my health from my medical notes, and if applicable those of my child, and I give permission for them to do this.	☐ (Your initials)	OR	☐
14. I understand I may be contacted in the future to give my consent to take part in future studies similar to this project.	☐ (Your initials)	OR	☐ No
15. I am happy for a member of the research team to contact me about taking part in an interview about my experience in this trial.	☐ (Your initials)	OR	☐ No

_____________________ ___________ _________________

Name of participant: Date: Signature:

_____________________ ___________ _________________

Name of person taking consent: Date: Signature:

When completed: 1 for participant; 1 for researcher site file; 1 to be kept in medical notes

For peer review only

For peer review only - http://bmjopen.bmj.com/site/about/guidelines.xhtml

STOP-OHSS Simulation Report Final Version 2.0

STOP-OHSS Trial Simulation Report: Summary report on sample size calculation and operating characteristics of the group sequential trial

Munya Dimairo [a], Laura Sutton [b], Tracey Young [c], Ellen Lee [a], Cara Mooney [a], and David White [a]

[a] *Clinical Trial Research Unit (CTRU); Design, Trials and Statistics (DTS), School of Health and Related Research (ScHARR); University of Sheffield*

[b] *DTS, ScHARR, University of Sheffield*

[c] *Health Economics and Decision Science (HEDS), ScHARR, University of Sheffield*

The contribution of the authors is described in Section 10.

For peer review only - http://bmjopen.bmj.com/site/about/guidelines.xhtml

STOP
OHSS

STOP-OHSS Simulation Report Final Version 2.0

Contents

1 Background 3
2 Findings from a retrospective audit study 3
2.1 Characterisation of the eligible patient population 3
2.2 Estimates of hospitalisation rate in the eligible population 3
2.3 Summary 4
3 Design, trial adaptations and interim decision-making criteria 4
3.1 The design 4
3.2 Trial adaptations and interim analysis aspects 4
3.3 Interim decision-making criteria 5
4 Sample size calculation 5
5 Statistical simulations 6
5.1 Objectives 6
5.2 Approach to simulations 6
6 Results 6
6.1 Summary of sample sizes 6
6.2 Impact of futility thresholds and uncertainty in usual care event rate 7
6.2.1 Impact on the probability of declaring efficacy correctly or in error. 8
6.2.2 Impact on the probability of stopping early for futility correctly or in error. 9
7 Summary 11
8 Limitations 11
9 Impact of early stopping on study duration and sample size saving 12
10 Contributions 12
11 Acknowledgements 12
12 References 12

For peer review only

STOP OHSS

STOP-OHSS Simulation Report Final Version 2.0

1 Background

This report summarises findings from a retrospective review of medical records of patients undergoing in-vitro fertilisation (IVF) treatments that inform the redesign of the STOP-OHSS trial. In addition, the design features of the STOP-OHSS trial, sample size estimates, and the statistical behaviour of the design in addressing the research questions under different assumptions are presented. Finally, the limitations of the design and implications of early stopping on trial duration are also discussed.

2 Findings from a retrospective audit study

This study was undertaken to get a better understanding of the pool of potentially eligible patients with early and late ovarian hyperstimulation syndrome (OHSS) to assess the feasibility of the initially proposed design and recruitment. Importantly, this was also to estimate the OHSS-related hospitalisation rate in the usual care to validate the assumptions made during the design of the original design or use this to modify the design accordingly. Finally, we wanted to understand how the usual care hospitalisation rate differs by proposed study populations (early versus late OHSS) and severity of OHSS (moderate versus severe).

2.1 Characterisation of the eligible patient population

Across five IVF sites, a total of 134 cycles from 134 patients were returned from searches performed over a year (1st March 2019 to 29th February 2020), of which, 63 would have been eligible to be recruited into this trial Of these 63 eligible patients, 25 (39.7%) had early OHSS and 38 (60.3%) had late OHSS. In addition, most patients 73.0% (46/63) were moderate OHSS cases (95% confidence interval, CI: 60.3% to 83.4%) and only 27.0% (17/63) were severe OHSS cases (95% CI: 16.6% to 39.7%).

2.2 Estimates of hospitalisation rate in the eligible population

Of the 63 eligible patients, 26 were hospitalised relating to OHSS, which translated to a 41.2% mean hospitalisation rate across five sites (95% CI: 29.0% to 54.4%).

Only 11 of the 25 eligible patients with early OHSS were hospitalised, representing a mean hospitalisation rate of 44.0% (95% CI: 24.4% to 65.1%). In the late OHSS population, 15 of the 38 eligible patients were hospitalised, translating to a mean hospitalisation rate of 39.5% (95 %CI: 24.0% to 56.6%).

Of the 46 eligible patients with moderate OHSS, 16 (34.8%) were hospitalised (95% CI: 21.4% to 50.2%). In 17 eligible patients with severe OHSS, there was a 58.8% (10/17) hospitalisation rate (95% CI: 32.9% to 81.6%).

For peer review only - http://bmjopen.bmj.com/site/about/guidelines.xhtml

STOP-OHSS Simulation Report Final Version 2.0

2.3 Summary

These estimates are based on OHSS patients from only 5 sites of the expected 20 sites so results should be interpreted with existing uncertainties in mind. However, the estimates give us some insights into eligible patients in the early and late OHSS populations, hospitalisation rates and how this varies by subpopulation and severity of OHSS. Specifically, cases of late OHSS appear to be higher than early OHSS, which is the opposite of what was expected. This strongly suggests that it is unlikely to be feasible to conduct a three-arm definitive trial in the early OHSS population as originally designed to investigate the effectiveness of early outpatient management interventions using paracentesis or a gonadotrophin-releasing hormone (GnRH) antagonist drug compared to usual care. Similarly, the cases of late OHSS seem to be much higher than expected so there is scope for a trial that is more than just exploratory as originally envisaged.

As a result, the Trial Management Group agreed to make changes to the original design by dropping one of the proposed study treatments in the early OHSS population (GnRH antagonist drug) and combining the early and late OHSS populations. Response to treatment in the early and late OHSS population is expected to be similar although the underlying hospitalisation rate may differ as shown in Section 2.2.

3 Design, trial adaptations and interim decision-making criteria

3.1 The design

In an open-label, pragmatic, multi-centre, superiority, parallel-group, group sequential, randomised controlled trial; participants with either early or late OHSS will be individually randomised (1:1) to receive early outpatient paracentesis (OP) or usual care (which excludes early OP). The primary outcome is any OHSS-related hospitalisation for at least 24 hours within 28 days from randomisation.

3.2 Trial adaptations and interim analysis aspects

The goal of the trial is to gather convincing evidence about the effects of the early OP that is most likely to change practice regardless of the direction of results. The recruitment to this trial is expected to be quite challenging so there is a need to pursue the trial only when the study treatment is viewed as promising to use research resources, study patients, and time efficiently. This motivated the use of a group sequential design with an option for early stopping for futility (lack of benefit). The decision to stop for futility is non-binding in the sense that it is advisory so it can be ignored or overruled for some reasons without affecting the statistical behaviour of the design (i.e., it does not increase the type I error beyond the budgeted 2.5% for a one-sided test). One interim analysis will be performed when 65% of the maximum sample size had accrued primary outcome data on hospitalisation within 28 days of randomisation. This delayed timing of interim analysis has been chosen to allow sufficient data to be gathered on hospitalisation rates in both arms to inform reliable interim decisions.

For peer review only - http://bmjopen.bmj.com/site/about/guidelines.xhtml

STOP-OHSS Simulation Report Final Version 2.0

Of note, the trial does not allow for early stopping for overwhelming benefit (efficacy). In addition, the continuation of the trial after interim analysis does not necessarily imply that the study treatment is effective but it only indicates that early OP is promising and worth pursuing further to assess if it is truly effective at the end.

3.3 Interim decision-making criteria

Decision rules at an interim analysis should be fit for purpose and decisions made should be reliable, sensible, and yield convincing and interpretable results. If early OP is found to be effective, the goal is to reduce the hospitalisation rate thus reducing the burden on the NHS, patients, and society, which is cost-saving. To claim the efficacy of early OP, the research team has targeted a realistic and worthwhile 20% reduction in hospitalisation rate that is viewed to be important to influence practice. To inform early stopping, the minimum reduction in hospitalisation below which early OP is viewed as futile needs to be predefined. In addition, a good stopping rule should balance the need to reduce the risk of stopping early for futility of a treatment that is potentially cost-saving and/or effective with the desire to increase the chances of early stopping when treatment is unlikely to be cost-saving and clinically effective.

To help achieve this, a preliminary health economic model which was developed at the grant development stage was updated based on estimated costs associated with early OP and usual care and results from a retrospective audit study (Section 2). This was done under several assumptions about the ratio of early to late OHSS and underlying hospitalisation rates assuming a 20% targeted reduction (if early OP is effective). **Results showed that early OP needs to achieve at least between 4.7% and 5.7% reduction in hospitalisation to become cost-saving** (see appendix found in protocol https://www.isrctn.com/ISRCTN71978064). As such, a sensible futility stopping rule needs to use a value less than 4.7%, which is only 23.5% of the targeted effect of 20% reduction. To further guide the choice of the futility threshold, we explored the statistical behaviour of the design through simulations using futility thresholds (hospital reduction) of 0, -2.5%, -3.0%, -3.5%, -4.0%, and -4.5% (see Section 6.2).

4 Sample size calculation

Sample sizes were estimated using the *rpact* [1] R package without continuity correction under the following assumptions:

- 1:1 allocation ratio, 90% power and 2.5% one-sided type I error rate;
- the best estimate of the usual care hospitalisation rate of 41% (Section 2.2);
- 20% targeted difference in hospitalisation rates between treatment arms;
- one interim analysis at 65% of the maximum total sample size;
- 0% dropout rate;
- futility stopping thresholds of 0, -2.5%, -3.0%, -3.5%, -4.0%, and -4.5%.

For peer review only - http://bmjopen.bmj.com/site/about/guidelines.xhtml

STOP-OHSS Simulation Report Final Version 2.0

5 Statistical simulations

5.1 Objectives

Simulations were undertaken to:

1) explore the impact of possible futility thresholds such as on the probability of stopping the trial under a specific assumption about the known treatment effect and the decision-making errors (e.g. incorrectly stopping for futility when the early OP is actually effective);
2) help the research team select an appropriate stopping threshold to use;
3) explore the impact of existing uncertainty in the usual care hospitalisation rate (which can vary from 29% to 54%) on study power.

5.2 Approach to simulations

Simulations were performed as follows in that order:

1. use the calculated 224 maximum total sample size (112 per arm) corresponding to a futility threshold of -4.5% (reduction in hospitalisation);
2. generate pseudo-trial data with the number of patients calculated as in item 1 using a binomial distribution assuming a certain treatment effect (differences in proportions of 0%, 5%, 10%, 15%, and 20% were used) and underlying usual care event rate (hospitalisation rate of 29% to 50% with an increment of 3% were used);
3. for each scenario, repeat the process to generate 100 000 trials sufficient to produce accurate results;
4. for each simulated pseudo-trial, analyse interim data from the first 65% of participants and decide whether the trial should have been stopped at this point using a selected futility threshold. This was repeated for futility thresholds of 0, -2.5%, -3.0%, -3.5%, -4.0%, and -4.5% (hospitalisation reduction);
5. for each simulated pseudo-trial, what the overall decision about clinical efficacy would have been if it was not stopped early was noted;
6. using data from all 100 000 simulated pseudo-trials, the proportions of trials that would have been stopped for futility and those that indicated efficacy if they were not stopped early were calculated.

All simulations were performed in Stata v16.1 using a bespoke program and results are presented in Section 6.2.

6 Results

6.1 Summary of sample sizes

Table 1 summarises the total maximum sample sizes and total sample sizes required for interim analysis under different assumptions. For example (row marked in green), the trial

For peer review only - http://bmjopen.bmj.com/site/about/guidelines.xhtml

STOP OHSS

STOP-OHSS Simulation Report Final Version 2.0

will require a maximum total sample size of ~224 (112 per arm), rounded upwards to the nearest even number. An interim analysis will be performed when ~146 participants (73 per arm) had accrued primary outcome data on hospitalisation within 28 days of randomisation. This assumes a 90% power, one-sided 2.5% type 1 error rate, 41% hospitalisation rate in the usual care arm (see Section 2.2), a 20% worthwhile and realistic reduction in hospitalisation rate (as per original design), and a futility threshold of -4.5% (difference in hospitalisation rate). In this case, a trial will be stopped early for futility if the observed reduction in hospitalisation rate at an interim analysis is less than 4.5% (equivalent to a one-sided p-value above 2.9 or critical value below 0.553). There is only a **1.9% chance** of stopping the trial early for futility in error for a beneficial treatment which is very low as expected.

As noted in Table 1, the total sample size is very similar across futility thresholds (0% to 4.5%). However, the influence of the size of the futility threshold on the chances of stopping early is apparent in Figure 2.

Table 1. Estimated total sample sizes assuming 90% power and 2.5% one-sided type I error.

Usual care event rate	A targeted difference in hospitalisation rates	Futility stopping boundaries at interim analysis			Efficacy boundaries at:		Sample sizes across groups		Probability of early stopping when paracentesis is efficacious
		Critical value	Difference in proportions (paracentesis – usual care)	One-sided p-value	An interim analysis	The final analysis	Interim analysis (65% of the total)	In total	
41%	-20%	0	0	0.50	N/A	-0.126	144	221	0.0045
41%	-20%	0.305	-0.025	0.38	N/A	-0.125	144	222	0.0104
41%	-20%	0.366	-0.030	0.357	N/A	-0.125	145	222	0.0121
41%	-20%	0.429	-0.035	0.334	N/A	-0.125	145	222	0.0142
41%	-20%	0.491	-0.040	0.312	N/A	-0.125	145	223	0.0165
41%	-20%	0.553	-0.045	0.290	N/A	-0.125	145	223	0.0191

Assumptions: 1:1 randomisation; 0% dropout rate; interim analysis to be performed when 65% of the maximum total sample size had accrued outcome data; NA, not applicable.

6.2 Impact of futility thresholds and uncertainty in usual care event rate

A good trial design should facilitate reliable decisions with high probability both at interim analysis and at the end if it progressed beyond interim analysis. For example, if we simulate many pseudo trials assuming that a treatment is effective with a targeted treatment effect we wish to see (e.g., 20%), a very high proportion of these trials should progress after interim analysis and the treatment should be deemed efficacious in the end. On the contrary, if we simulate assuming that a treatment does not work (0% effect say), we should expect most trials to indicate early stopping and only a negligible fraction to indicate efficacy in the end (i.e., making erroneous conclusion claiming efficacy, type I error).

For peer review only - http://bmjopen.bmj.com/site/about/guidelines.xhtml

STOP OHSS

STOP-OHSS Simulation Report Final Version 2.0

6.2.1 Impact on the probability of declaring efficacy correctly or in error.

As evident in Figure 1, across all presented scenarios, the choice of futility threshold between -4.5% and 0 has a negligible effect on the chances of declaring efficacy either correctly or incorrectly.

Figure 1 **(A)** shows the power of the study as the usual care hospitalisation rate changes from 29% to 50%. If the underlying hospital reduction is as assumed (20%), the study will still have more than 87% power even if the usual care hospitalisation rate is as high as 50%. That is, regardless of the usual care hospitalisation rate, the study will have sufficient power (above or close to the assumed 90%) to find a 20% hospital reduction if the treatment is truly effective with this effect.

On the contrary, in Figure 1 **(B)**, if the observed hospital reduction is 15% (rather than the assumed 20%), the power of the study will range from 62% to 79% to find a 15% hospital reduction if early OP is effective with this effect as the usual care hospitalisation rate decreases from 50% to 29%, respectively. Figure 1 **(C)** shows that the study will be severely underpowered to find a 10% hospital reduction across all scenarios of the usual care hospitalisation rate even though this effect is likely to be cost-saving based on the updated preliminary health economic model.

Figure 1 **(E)** shows the chances of erroneously claiming that early OP is effective when its hospitalisation rate is the same as in the usual care (i.e., 0% hospital reduction). This error is very small as expected and ranges from 2.4% to 2.6% across (accounting for Monte Carlo error) all scenarios compared to the targeted 2.5% for a one-sided test.

In summary, if the underlying treatment effect is at least 20% hospital reduction, the study will be sufficiently powered across all realistic scenarios of the usual care hospitalisation rate and this is not affected by the choice of the futility threshold of between -4.5% and 0%. In addition, the chance of incorrectly declaring efficacy when treatment is ineffective is very small and as expected. However, the study will be severely underpowered for effects that are far less than 20% but likely to be cost-saving (e.g., 5% to 15%).

For peer review only

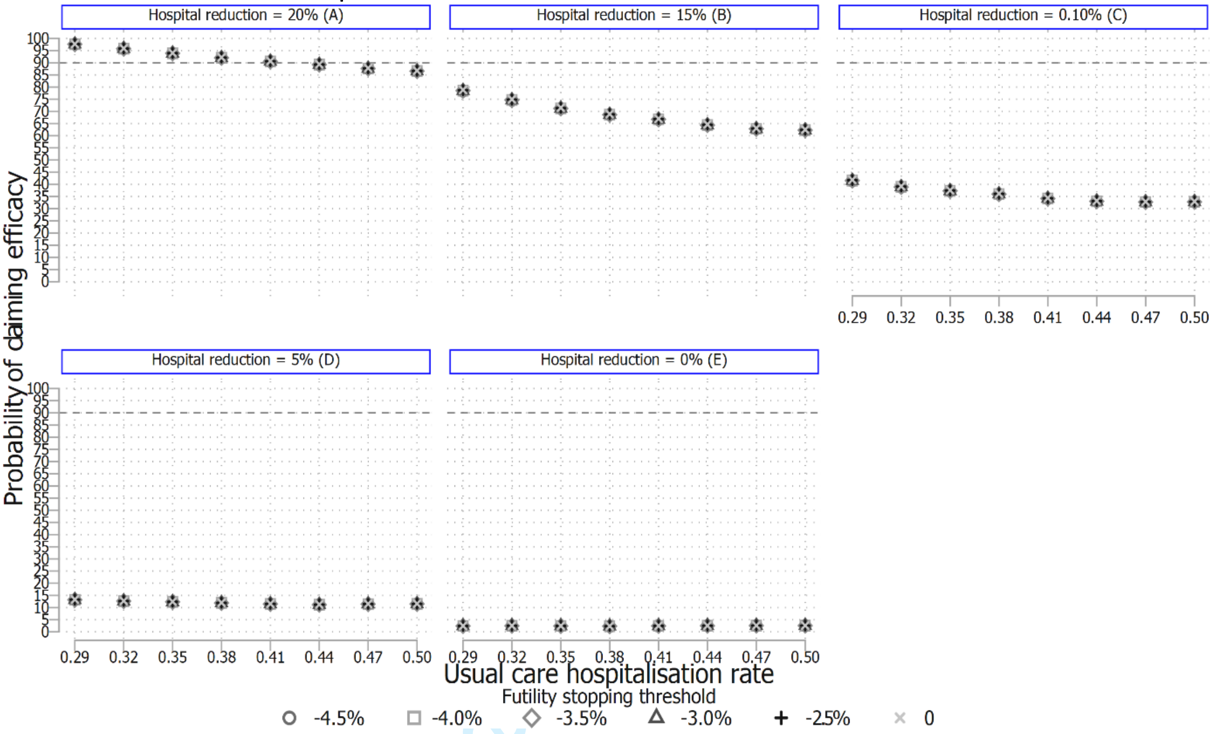

Figure 1. Impact on the chances of claiming efficacy at the end of the trial.

6.2.2 Impact on the probability of stopping early for futility correctly or in error.

Figure 2 presents the chances of stopping early for futility under different assumptions of the underlying treatment effect, usual care hospitalisation rate, and futility stopping threshold. As evident, the impact of futility stopping threshold becomes more apparent as the underlying hospital reduction changes from 20% to 0%. In principle, we want a design that:

1) allows early stopping with high probability when the underlying treatment effect is in support of the null hypothesis of no difference and,
2) erroneously leads to trial early stopping with very low or negligible probability when it is known that the treatment is beneficial.

In Figure 2 (A), if early OP is effective, the chances of stopping early range from 0% to 2.9% as the futility stopping threshold decreases from 0 to -4.5% and the usual care hospitalisation rate increases from 29% to 50%, respectively. For example, if the underlying treatment effect is 20% as hoped and the usual care event rate is 41% (current best estimate), there is only approximately a 2.2% chance of stopping early for futility if a stopping threshold of -4.5% is used.

Figure 2 (E) is a scenario when it is known that the treatment effect is **0%** and a good design should trigger early stopping with high probability. As evident, this is achieved across all scenarios when a futility stopping threshold of -4.5% is chosen. For example, this probability is 72.2% when the usual care hospital rate is 41% (current best estimate).

For peer review only - http://bmjopen.bmj.com/site/about/guidelines.xhtml

STOP-OHSS Simulation Report Final Version 2.0

Figure 2 (subfigures **B-D**) are interpreted in the same manner when the underlying treatment effect is 15%, 10%, and 5% respectively.

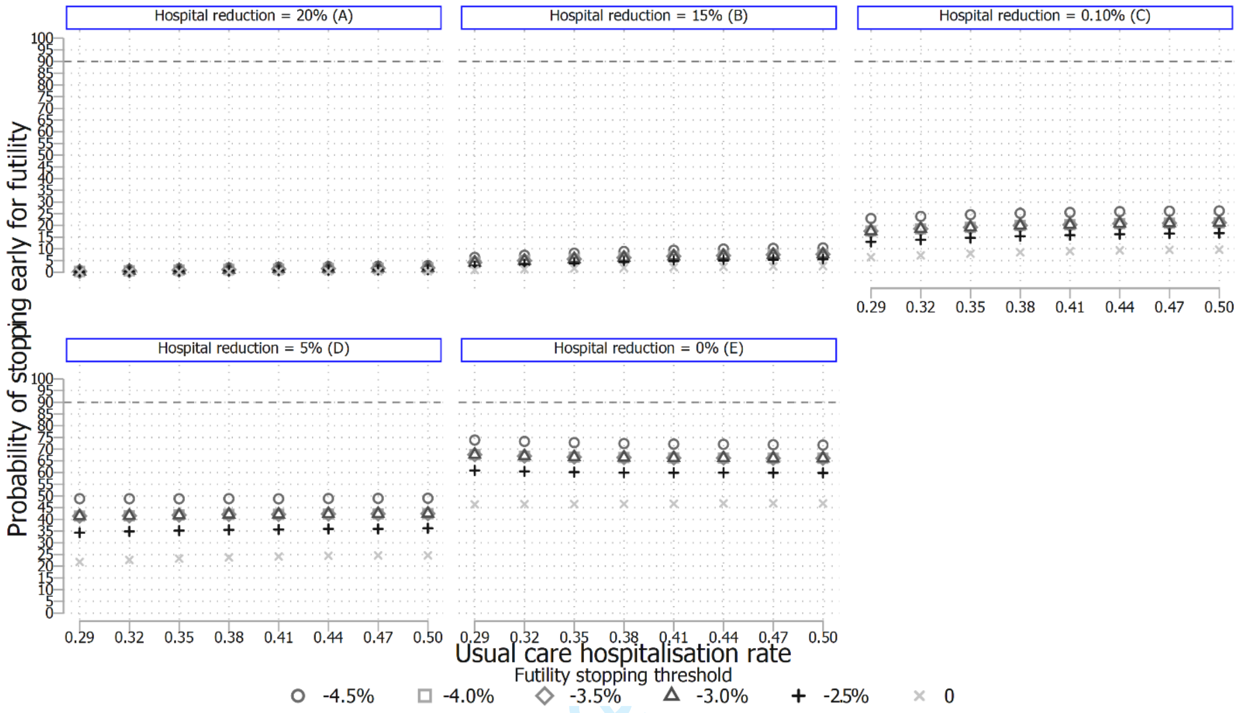

Figure 2. Impact on the chances of stopping early correctly or in error.

In summary, when choosing a futility stopping threshold, there is a trade-off between increasing the chances of early stopping for an ineffective treatment (scenario **E**) and reducing the risks of stopping a trial early for futility erroneously (scenario **A**).

For peer review only - http://bmjopen.bmj.com/site/about/guidelines.xhtml

STOP OHSS

STOP-OHSS Simulation Report Final Version 2.0

7 Summary

The trial will require a maximum total of 224 participants (112 per arm) to maintain a power of 90% for a 2.5% one-sided assuming a 41% hospitalisation rate in the usual care and a large targeted reduction of 20%. One interim analysis will be performed to inform whether the trial should be stopped early for futility when 65% of the maximum total sample size had accrued primary outcome data. It has been shown that the trial will be sufficiently powered across realistic event rates (e.g., 29% to 50%) as long as the targeted effect of a 20% reduction is realistic. Key limitations of the design are highlighted in Section 8.

In this trial, it has been shown in Figure 1 that the choice of futility stopping threshold had a negligible effect on the power and type I error rate of the study across the usual care event rate considered. However, as evident in Figure 2, the choice of futility stopping threshold has a significant effect on the chances of correctly stopping for futility and a very small effect on the probability of erroneously stopping early for futility.

The choice of a **4.5% hospital reduction as a futility stopping threshold appears very reasonable** for four specific reasons. First, it is below the lower bound of hospital reduction likely to result in cost savings. Second, it gives a higher chance of stopping the trial early when then the treatment is known to be ineffective which is the motivation for futility early stopping. Third, the risk of erroneously stopping the trial early for futility when the treatment is known to be effective is very small. Finally, it does not affect the power of the study.

The final decision on which futility stopping threshold to use lies with the research team guided by the results presented and the levels of risk they are willing to tolerate. Notably, in this design, the chosen futility stopping threshold is advisory so it can be overruled or ignored by the independent data monitoring committee and research team without affecting the design negatively.

Finally, the group sequential design considered here is feasible to implement and the primary outcome is observed within 28 days of randomisation and recruitment in this population is expected to be very slow.

8 Limitations

One major limitation of the design is that it is based on a large treatment effect of 20% hospital reduction which is viewed as realistic (based on data from observational studies) and worthwhile to change practice. However, as noted from the updated health economic model, hospital reductions of about 4.7% to 5.7% are also likely to result in cost savings. As a result, there is a region where the treatment effect is likely to be cost-saving (e.g., 10% to 15%) but the trial will be severely underpowered for such small to moderate treatment effects. This will complicate the interpretation of results if such effects turn out to be true. Finally, the treatment effect of 20% becomes unrealistic and impossible to achieve as the usual care event rate gets closer to 20%, however, the hospitalisation rate in the usual care is likely to be much higher than 20% based on the current best estimate presented in Section 2.2.

For peer review only - http://bmjopen.bmj.com/site/about/guidelines.xhtml

STOP OHSS

9 Impact of early stopping on study duration and sample size saving

An audit review of medical records suggested that the average recruitment across 20 sites will be around 4.6 patients per month. Stopping the trial early for futility at an interim analysis implies that an additional total of 78 participants would no longer be recruited. The trial will not be paused during interim analysis and interim decision-making process as the recruitment rate is expected to be very slow. As such, even if the trial is stopped early, we would expect negligible participants to have been recruited during interim analysis (e.g., at most 2 participants per arm). At this point, if we assume a constant average recruitment rate of 4.6 patients per month across all sites and staggered opening of sites to recruitment starting with 2 followed by 5, 8, 11, 15, 18, and 20 in consecutive months, then 10 months of further recruitment will no longer be required. Finally, potential cost savings can be extrapolated under different scenarios.

10 Contributions

Munya Dimairo designed the simulation study, wrote the simulation code, conducted simulations, and drafted the simulation report.

Laura Sutton performed statistical quality control in R relating to results presented in Table 1.

Ellen Lee performed statistical quality control in Stata relating to the simulation code.

Tracey Young contributed to the preliminary health economic model that informed the futlity stopping threshold.

David White and Cara Mooney reviewed the report and contributed to Section 9.

11 Acknowledgements

We are very grateful for the invaluable feedback from the Trial Steering Committee independent statistician (Babak Choodari-Oskooei, Medical Research Council (MRC) Clinical Trial Unit (CTU) at University College London (UCL)). Finally, we would like to thank the Trial Management Group (TMG) responsible for the day-to-day conduct of the trial.

12 References

1. Wassmer, G. & Pahlke, F. RPACT Package Overview | RPACT.

For peer review only - http://bmjopen.bmj.com/site/about/guidelines.xhtml

SPIRIT

STANDARD PROTOCOL ITEMS: RECOMMENDATIONS FOR INTERVENTIONAL TRIALS

SPIRIT 2013 Checklist: Recommended items to address in a clinical trial protocol and related documents*

Section/item	Item No	Description	Addressed on page number
**Administrative information**			
Title	1	Descriptive title identifying the study design, population, interventions, and, if applicable, trial acronym	_____1_________
Trial registration	2a	Trial identifier and registry name. If not yet registered, name of intended registry	___3 & 19______
	2b	All items from the World Health Organization Trial Registration Data Set	_______________
Protocol version	3	Date and version identifier	_____1_________
Funding	4	Sources and types of financial, material, and other support	_____20________
Roles and responsibilities	5a	Names, affiliations, and roles of protocol contributors	___1 & 19______
	5b	Name and contact information for the trial sponsor	_______1________
	5c	Role of study sponsor and funders, if any, in study design; collection, management, analysis, and interpretation of data; writing of the report; and the decision to submit the report for publication, including whether they will have ultimate authority over any of these activities	_____NA______
	5d	Composition, roles, and responsibilities of the coordinating centre, steering committee, endpoint adjudication committee, data management team, and other individuals or groups overseeing the trial, if applicable (see Item 21a for data monitoring committee)	_____14________

For peer review only

Introduction

Background and rationale	6a	Description of research question and justification for undertaking the trial, including summary of relevant studies (published and unpublished) examining benefits and harms for each intervention	____5-6______
	6b	Explanation for choice of comparators	____5-6______
Objectives	7	Specific objectives or hypotheses	____6________
Trial design	8	Description of trial design including type of trial (eg, parallel group, crossover, factorial, single group), allocation ratio, and framework (eg, superiority, equivalence, noninferiority, exploratory)	____6________

Methods: Participants, interventions, and outcomes

Study setting	9	Description of study settings (eg, community clinic, academic hospital) and list of countries where data will be collected. Reference to where list of study sites can be obtained	_____7________
Eligibility criteria	10	Inclusion and exclusion criteria for participants. If applicable, eligibility criteria for study centres and individuals who will perform the interventions (eg, surgeons, psychotherapists)	____8-9_______
Interventions	11a	Interventions for each group with sufficient detail to allow replication, including how and when they will be administered	___9-10_______
	11b	Criteria for discontinuing or modifying allocated interventions for a given trial participant (eg, drug dose change in response to harms, participant request, or improving/worsening disease)	_____10_______
	11c	Strategies to improve adherence to intervention protocols, and any procedures for monitoring adherence (eg, drug tablet return, laboratory tests)	_____10_______
	11d	Relevant concomitant care and interventions that are permitted or prohibited during the trial	____10_______
Outcomes	12	Primary, secondary, and other outcomes, including the specific measurement variable (eg, systolic blood pressure), analysis metric (eg, change from baseline, final value, time to event), method of aggregation (eg, median, proportion), and time point for each outcome. Explanation of the clinical relevance of chosen efficacy and harm outcomes is strongly recommended	____11________
Participant timeline	13	Time schedule of enrolment, interventions (including any run-ins and washouts), assessments, and visits for participants. A schematic diagram is highly recommended (see Figure)	____Figure 1____

For peer review only - http://bmjopen.bmj.com/site/about/guidelines.xhtml

Sample size	14	Estimated number of participants needed to achieve study objectives and how it was determined, including clinical and statistical assumptions supporting any sample size calculations	____13_______
Recruitment	15	Strategies for achieving adequate participant enrolment to reach target sample size	____7-8______
**Methods: Assignment of interventions (for controlled trials)**			
Allocation:			
Sequence generation	16a	Method of generating the allocation sequence (eg, computer-generated random numbers), and list of any factors for stratification. To reduce predictability of a random sequence, details of any planned restriction (eg, blocking) should be provided in a separate document that is unavailable to those who enrol participants or assign interventions	_____9_____
Allocation concealment mechanism	16b	Mechanism of implementing the allocation sequence (eg, central telephone; sequentially numbered, opaque, sealed envelopes), describing any steps to conceal the sequence until interventions are assigned	____9______
Implementation	16c	Who will generate the allocation sequence, who will enrol participants, and who will assign participants to interventions	____9______
Blinding (masking)	17a	Who will be blinded after assignment to interventions (eg, trial participants, care providers, outcome assessors, data analysts), and how	___13-14_____
	17b	If blinded, circumstances under which unblinding is permissible, and procedure for revealing a participant's allocated intervention during the trial	___NA_______
**Methods: Data collection, management, and analysis**			
Data collection methods	18a	Plans for assessment and collection of outcome, baseline, and other trial data, including any related processes to promote data quality (eg, duplicate measurements, training of assessors) and a description of study instruments (eg, questionnaires, laboratory tests) along with their reliability and validity, if known. Reference to where data collection forms can be found, if not in the protocol	_____11-12____
	18b	Plans to promote participant retention and complete follow-up, including list of any outcome data to be collected for participants who discontinue or deviate from intervention protocols	____10______

Data management	19	Plans for data entry, coding, security, and storage, including any related processes to promote data quality (eg, double data entry; range checks for data values). Reference to where details of data management procedures can be found, if not in the protocol	______18_______
Statistical methods	20a	Statistical methods for analysing primary and secondary outcomes. Reference to where other details of the statistical analysis plan can be found, if not in the protocol	____15-17_____
	20b	Methods for any additional analyses (eg, subgroup and adjusted analyses)	____15-17_____
	20c	Definition of analysis population relating to protocol non-adherence (eg, as randomised analysis), and any statistical methods to handle missing data (eg, multiple imputation)	_____16_______
**Methods: Monitoring**			
Data monitoring	21a	Composition of data monitoring committee (DMC); summary of its role and reporting structure; statement of whether it is independent from the sponsor and competing interests; and reference to where further details about its charter can be found, if not in the protocol. Alternatively, an explanation of why a DMC is not needed	____14_________
	21b	Description of any interim analyses and stopping guidelines, including who will have access to these interim results and make the final decision to terminate the trial	____14-15______
Harms	22	Plans for collecting, assessing, reporting, and managing solicited and spontaneously reported adverse events and other unintended effects of trial interventions or trial conduct	_____13________
Auditing	23	Frequency and procedures for auditing trial conduct, if any, and whether the process will be independent from investigators and the sponsor	____18_________
**Ethics and dissemination**			
Research ethics approval	24	Plans for seeking research ethics committee/institutional review board (REC/IRB) approval	____19________
Protocol amendments	25	Plans for communicating important protocol modifications (eg, changes to eligibility criteria, outcomes, analyses) to relevant parties (eg, investigators, REC/IRBs, trial participants, trial registries, journals, regulators)	______7________

For peer review only - http://bmjopen.bmj.com/site/about/guidelines.xhtml

Consent or assent	26a	Who will obtain informed consent or assent from potential trial participants or authorised surrogates, and how (see Item 32)	_____8_______
	26b	Additional consent provisions for collection and use of participant data and biological specimens in ancillary studies, if applicable	_____NA_______
Confidentiality	27	How personal information about potential and enrolled participants will be collected, shared, and maintained in order to protect confidentiality before, during, and after the trial	____18______
Declaration of interests	28	Financial and other competing interests for principal investigators for the overall trial and each study site	____20_______
Access to data	29	Statement of who will have access to the final trial dataset, and disclosure of contractual agreements that limit such access for investigators	____18________
Ancillary and post-trial care	30	Provisions, if any, for ancillary and post-trial care, and for compensation to those who suffer harm from trial participation	_______NA______
Dissemination policy	31a	Plans for investigators and sponsor to communicate trial results to participants, healthcare professionals, the public, and other relevant groups (eg, via publication, reporting in results databases, or other data sharing arrangements), including any publication restrictions	____19_______
	31b	Authorship eligibility guidelines and any intended use of professional writers	_____19_______
	31c	Plans, if any, for granting public access to the full protocol, participant-level dataset, and statistical code	_____NA______
**Appendices**			
Informed consent materials	32	Model consent form and other related documentation given to participants and authorised surrogates	_____Supp______
Biological specimens	33	Plans for collection, laboratory evaluation, and storage of biological specimens for genetic or molecular analysis in the current trial and for future use in ancillary studies, if applicable	_______NA______

*It is strongly recommended that this checklist be read in conjunction with the SPIRIT 2013 Explanation & Elaboration for important clarification on the items. Amendments to the protocol should be tracked and dated. The SPIRIT checklist is copyrighted by the SPIRIT Group under the Creative Commons "Attribution-NonCommercial-NoDerivs 3.0 Unported" license.
