# Supplementary material for: Outpatient paracentesis for the management of ovarian hyperstimulation syndrome: study protocol for the STOP-OHSS randomised controlled trial
Source: BMJ Open. 2024 Jan 22;14(1):e076434. doi: 10.1136/bmjopen-2023-076434 (PMC10806818; doi:10.1136/bmjopen-2023-076434)
Supplement: Reviewer comments [file bmjopen-2023-076434.reviewer_comments.pdf]

PEER REVIEW HISTORY

BMJ Open publishes all reviews undertaken for accepted manuscripts. Reviewers are asked to complete a checklist review form (**http://bmjopen.bmj.com/site/about/resources/checklist.pdf**) and are provided with free text boxes to elaborate on their assessment. These free text comments are reproduced below.

ARTICLE DETAILS

**TITLE (PROVISIONAL)**	Outpatient paracentesis for the management of Ovarian HyperStimulation Syndrome: Study protocol for the STOP-OHSS randomised controlled trial
**AUTHORS**	White, David; Pye, Clare; Ridsdale, Katie; Dimairo, Munyaradzi; Mooney, Cara; Wright, Jesssica; Young, Tracey; Cheong, Ying; Drakeley, Andrew; Mathur, Raj; O'Cathain, Alicia; Desoysa, Lauren; Sizer, Anya; Lumley, Elizabeth; Chatters, Robin; Metwally, Mostafa

This article was not externally reviewed at BMJ Open. Protocols that have been independently assessed prior to submission to BMJ Open are usually fast-tracked to publication on the grounds that further substantial changes will not be possible. This independent assessment will usually be external, independent review for both a substantial grant award from a non-commercial or government funder and ethics approval.
